# Discovery of highly immunogenic spleen-resident FCGR3^+^CD103^+^ cDC1s differentiated by IL-33-primed ST2^+^ basophils

**DOI:** 10.1038/s41423-023-01035-8

**Published:** 2023-05-29

**Authors:** Myeong-Ho Kang, JungHyub Hong, Jinjoo Lee, Min-Suk Cha, Sangho Lee, Hye-Young Kim, Sang-Jun Ha, Yong Taik Lim, Yong-Soo Bae

**Affiliations:** 1 Department of Biological Sciences, Sungkyunkwan University, 2066 Seobu-ro, Jangan-gu, Suwon-si, Gyeonggi-do 16419 Republic of Korea; 2 Center for Immune Research on Non-Lymphoid Organs, Sungkyunkwan University, 2066 Seobu-ro, Jangan-gu, Suwon-si, Gyounggi-do 16419 Republic of Korea; 3 Laboratory of Mucosal Immunology, Department of Biomedical Sciences, Seoul National University College of Medicine, 103 Daehak-ro, Jongno-gu, Seoul, 03080 Republic of Korea; 4 Institute of Allergy and Clinical Immunology, Seoul National University Medical Research Center, Seoul, Republic of Korea; 5 Department of Biochemistry, College of Life Science and Biotechnology, Yonsei University, Seoul, 03722 Republic of Korea; 6 Department of Nano Engineering and School of Chemical Engineering, Sungkyunkwan University, 2066 Seobu-ro, Jangan-gu, Suwon-si, Gyeonggi-do 16419 Republic of Korea

**Keywords:** Recombinant interleukin-33 (IL-33), Highly immunogenic, Spleen residency, FCGR3^+^CD103^+^ cDC1s, ST2^+^ basophils, Antitumor immunity, Conventional dendritic cells, Immunotherapy

## Abstract

Recombinant interleukin-33 (IL-33) inhibits tumor growth, but the detailed immunological mechanism is still unknown. IL-33-mediated tumor suppression did not occur in Batf3^−/−^ mice, indicating that conventional type 1 dendritic cells (cDC1s) play a key role in IL-33-mediated antitumor immunity. A population of CD103^+^ cDC1s, which were barely detectable in the spleens of normal mice, increased significantly in the spleens of IL-33-treated mice. The newly emerged splenic CD103^+^ cDC1s were distinct from conventional splenic cDC1s based on their spleen residency, robust effector T-cell priming ability, and surface expression of FCGR3. DCs and DC precursors did not express Suppressor of Tumorigenicity 2 (ST2). However, recombinant IL-33 induced spleen-resident FCGR3^+^CD103^+^ cDC1s, which were found to be differentiated from DC precursors by bystander ST2^+^ immune cells. Through immune cell fractionation and depletion assays, we found that IL-33-primed ST2^+^ basophils play a crucial role in the development of FCGR3^+^CD103^+^ cDC1s by secreting IL-33-driven extrinsic factors. Recombinant GM-CSF also induced the population of CD103^+^ cDC1s, but the population neither expressed FCGR3 nor induced any discernable antitumor immunity. The population of FCGR3^+^CD103^+^ cDC1s was also generated in vitro culture of Flt3L-mediated bone marrow-derived DCs (FL-BMDCs) when IL-33 was added in a pre-DC stage of culture. FL-BMDCs generated in the presence of IL-33 (FL-33-DCs) offered more potent tumor immunotherapy than control Flt3L-BMDCs (FL-DCs). Human monocyte-derived DCs were also more immunogenic when exposed to IL-33-induced factors. Our findings suggest that recombinant IL-33 or an IL-33-mediated DC vaccine could be an attractive protocol for better tumor immunotherapy.

## Introduction

Interleukin-33 (IL-33) is a nuclear cytokine that is a member of the IL-1 family [1, 2]. IL-33 is also a well-known alarmin released by damaged or necrotic cells and is associated with type II immunity [3, 4]. Released IL-33 can be activated by an allergen-derived protease [3–5]. IL-33 binds to its primary receptor, ST2 [1, 6], and coreceptor, IL-1RAcP [7, 8]. In asthma and allergic inflammation, the IL-33/ST2 axis is known to be associated with the stimulation of type II immune cells, including basophils [9, 10], T helper 2 cells [6, 11], eosinophils [12], mast cells [13], and group-2 innate lymphoid cells (ILC2s) [14–16]. In a number of human and animal model studies of infections, inflammatory diseases, and cancers, IL-33 is a crucial immune modulator that shapes type 1, type 2 and regulatory immune responses [4], playing a critical role in stimulating CD8^+^ T cells [17, 18], regulatory T cells (Tregs) [19], macrophages [20, 21], dendritic cells (DCs) [22, 23], and NK cells [11, 24].

In tumors, the role of IL-33 is still controversial [25, 26]. In the tumor environment, the IL-33/ST2 axis was reported to promote Treg expansion and to alter the immune system in tumor-promoting ways [27–30]. In gastric cancer, IL-33-mediated mast cell activation promotes tumor growth by inducing tumor-associated macrophage mobilization [31]. IL-33-induced FcεRIa^+^ macrophages activate TGF-β signaling, which promotes tumor cell invasion [32]. IL-33 was also reported to directly induce the proliferation or migration of tumor-associated cells [33–35].

On the other hand, several studies have shown that IL-33 mediates antitumor immunity. Antitumor responses and antigen-specific T cells were efficiently induced when using IL-33 as an adjuvant [36]. IL-33-activated eosinophils inhibited tumor growth via degranulation and cytolytic cytokine secretion in syngeneic and metastatic tumor models [37–40]. Transgenic or tumoral expression of IL-33 also promotes antitumor responses by activating NK cells, CD8^+^ T cells, and CD4^+^ T cells [18, 24, 41–43].

Exogenous IL-33 restored tumor-residing dendritic cell (DC) activation and maturation via direct ST2-mediated signaling and induced antitumor T-cell responses in a syngeneic tumor model and pulmonary adenocarcinoma [44, 45]. Another report demonstrated that IL-33 treatment promotes antitumor responses by enhancing cell survival and the proliferation of DC-primed antitumor Tc9 (IL-9^+^ CD8^+^ T) cells [46]. In addition, tumor-derived IL-33 was shown to increase the number of CD103^+^ DCs in the tumor microenvironment and to reactivate the tumor-resident CD8^+^ T cells required for antitumor responses [41]. However, the detailed immunological mechanisms of recombinant IL-33-derived DC-mediated antitumor immunity remain unclear.

In this study, we found that recombinant IL-33 induces a de novo population of highly immunogenic organ-resident FCGR3^+^CD103^+^ cDC1s in the spleen of treated mice, and IL-33-primed ST2^+^ basophils play a crucial role in the development of this cDC1 population. Extrinsic factors involved in the development of FCGR3^+^CD103^+^ cDC1s and FCGR3^+^CD103^+^ cDC1-mediated antitumor immunity were investigated.

## Materials and methods

Detailed sources of materials and methods are available in the supplemental information.

### Mice

Wild-type (WT) C57BL/6 mice were purchased from DBL (Eumseong, Korea). The ST2^−/−^ (C.129P2-*Il1rl1*^*tm1Anjm*^) [6] mice were a gift from Dr. Andrew N. McKenzie (Medical Research Council Laboratory of Molecular Biology, Cambridge, United Kingdom). *Batf3*^−/−^ (B6.129S(C)-*Batf3*^*tm1Kmm*^/J), CD45.1 (B6.SJL-*Ptprc*^*a*^ *Pepc*^*b*^/BoyJ), and OT-I [C57BL/6-Tg (TcraTcrb)1100Mjb/J] mice were purchased from Jackson Laboratory. All mice were inbred on a C57BL/6 background and were maintained in a specific pathogen-free facility at Sungkyunkwan University according to the Institute/University Animal Care and Use guidelines.

### Flt3L-generated bone marrow–derived dendritic cells (FL-BMDCs or FL-DCs)

BM cells from the mice were cultured as described previously [47, 48] with minor modifications. In brief, the BM cells were suspended at a density of 2 × 10^6^/ml in complete RPMI containing 10% fetal bovine serum (FBS) and 100 U/ml penicillin/streptomycin (hereafter, cRPMI) supplemented with 100 ng/ml recombinant human Flt3L, 10 mM HEPES, and 55 μM β-mercaptoethanol. The cells were plated at 1 ml/well on 12-well plates and were incubated at 37 °C with 5% CO_2_ for 10 days. To generate FL-33-DCs or FL-GM-DCs, we added 5 ng/ml recombinant murine IL-33 (mIL-33) or GM-CSF (mGM-CSF) on Day 5 of FL-DC generation and incubated the cells for an additional 5 days. On the last day of culture, the cells were used for subsequent experiments.

### Administration of cytokines

Cytokines were administered to the mice using a method previously described [49] with minor modifications. In brief, 1 μg of mIL-33 or mGM-CSF was intraperitoneally injected into tumor-free (TF) mice once a day for 8 consecutive days. One day after the final injection, the spleens were collected and suspended as single cells that were used for the subsequent experiments.

### Ex vivo splenocyte culture

Splenocytes from WT and ST2-KO mice were suspended at a density of 2.5 × 10^6^ cells in cRPMI supplemented with 100 ng/ml recombinant human Flt3L, 10 mM HEPES, and 55 μM β-mercaptoethanol. The cells were plated on 12-well plates at 1 ml/well and were incubated for 2 days in the presence or absence of 5 ng/ml IL-33. Then, the cells were used for further analysis.

### Flow cytometric analysis and cell sorting (mouse)

Cells were analyzed on a FACSCanto II (BD Bioscience) and sorted on a FACSAria Fusion (BD Bioscience). The data analysis was performed using FlowJo software, version 10 (TreeStar). The detailed gating strategy for analysis and sorting is presented in the supplemental information.

### Intravascular staining

The methods for intravascular staining were described previously [50]. In brief, mice were inoculated intravenously with 3 μg of anti-CD11c-eF450 antibody in 300 μl of PBS. The mice were sacrificed at 3 min after the injection, and splenocytes were collected. For ex vivo staining of CD11c, an anti-CD11c-PEcy7 antibody was used.

### Mixed culture with cells isolated from WT and ST2-KO mice

BM cells or splenocytes were collected from CD45.1^+^ WT and CD45.2^+^ ST2-KO mice. The cells were mixed at a 1:1 ratio and cultured using the methods described previously in the absence or presence of mIL-33. WT or ST2-KO-derived cells were distinguished with anti-CD45.1-Pacific blue and anti-CD45.2-PerCPcy5.5 staining.

### Cell isolation for RNA-seq and data analysis

CD103^+^ cDC1s from FL-33-DCs or FL-GM-DCs and CD11c^**–**^ cells on Day 5 of WT or ST2-KO FL-DC culture were sorted using a BD FACSAria Fusion with a 70 μm nozzle. CD11c^**–**^ cells were treated with IL-33 for 6 h and then collected. Cell lysis, RNA purification, quality, and quantification were performed for bulk RNA-seq as described in the supplemental information. The detailed methods for RNA sequencing and data analysis are also available in the supplemental information. Raw data from the bulk RNA-seq analysis were deposited in the NCBI Gene Expression Omnibus (GEO) under accession numbers GSE226585 and GSE226586.

### Immune complexes

OVA protein-antibody immune complexes were generated as previously described with minor modifications [51]. In brief, mouse IgG1 anti-OVA (BioLegend) and OVA protein were incubated for 30 min at room temperature. XCR1^+^ cDC1s were isolated from FL-DCs and FL-GM-DCs. Among the CD103^+^ cDC1s from FL-33-DCs, FCGR3^-^ or FCGR3^+^ cells were additionally separated. These cells were treated with OVA-IC at 37 °C in 5% CO_2_ for 18 h in the cell culture medium with final concentrations of 130 μg/ml OVA and 280 μg/ml anti-OVA IgG1. Subsequently, OVA-IC-treated DCs (5 × 10^4^) were cocultured with 2.5 × 10^5^ OT-I CD8^+^ T cells for 4 days.

### ST2-KO DC precursors cocultured with WT BM cell fractions

CD11c^–^ and CD11c^+^ cells were isolated from WT splenocytes or on Day 5 of FL-DCs using FACSAria Fusion (BD Biosciences). Cells (1 × 10^6^) were cocultured with ST2-KO splenocytes for 2 days or ST2-KO FL-DCs on Day 5 for an additional 5 days. CD11c^–^ cells on Day 5 of FL-DC culture were divided into FcεRIα^–^ CD127^–^ non-ILCs, FcεRIα^–^ CD127^+^ ILCs, FcεRIα^+^ CD49b^–^ cells, and FcεRIα^+^ CD49b^+^ basophils. Non-ILCs (9 × 10^5^ cells) or other cell fractions (2 × 10^4^ cells) were cocultured with ST2-KO FL-DCs on Day 5 for an additional 5 days. WT splenic basophils were isolated from CD11c^–^ cells or depleted from CD11c^–^ cells by FACS sorting. Basophil-containing or basophil-depleted CD11c^–^ cells (1  × 10^6^) or FACS-sorted basophils (2 × 10^4^ cells) were cocultured with ST2-KO splenocytes for 2 days. Coculture experiments were performed in the absence or presence of 5 ng/ml IL-33.

### Cytokine blocking or ILC2 depletion in FL-33-DCs

FL-DCs on Day 5 were treated with rmIL-33 (5 ng/ml) together with anti-GM-CSF (10 μg/ml), anti-IL-13 (2.5 μg/ml), anti-IL-9 (10 μg/ml), anti-IL-5 (10 μg/ml), or anti-CD90.2 (10 μg/ml) and then were cultured for an additional 5 days. Then, the cells were collected and used for subsequent experiments.

### DC-based tumor immunotherapy

The tumor immunotherapy models used C57BL/6 WT mice, as described previously [52] with minor modifications. Detailed methods are available in the supplemental information.

### Human Mo-DC generation with IL-33-derived culture supernatants

Human monocytes and Lin^-^ cells were isolated from PBMCs of healthy volunteers. Detailed panels are shown in the supplemental information. Lin^–^ cells were cultured for 3 days in cRPMI with or without 10 ng/ml recombinant human IL-33 at a density of 5 × 10^5^/ml. Then, the culture supernatants were collected. We used monocyte culture methods as described previously [53] with minor modifications. In brief, monocytes were suspended at 5 × 10^5^/ml in cRPMI supplemented with recombinant human GM-CSF and IL-4 at 100 ng/ml and 10 ng/ml, respectively. After 3 days, the medium was replaced with fresh medium with all supplements, and the collected supernatants were administered at 50%. On Day 7, the cells were collected and used for the subsequent experiment.

### Coculture of hMo-DCs with allogenic CD8^+^ T cells

For allogenic CD8^+^ T-cell responses by hMo-DCs, Mo-DCs (5 × 10^4^) were cocultured with 1.5 × 10^5^ CTV-labeled allogenic CD8^+^ T cells for 4 days as described previously [54–56] with minor modifications. Before analysis, the cells were stimulated with a cell activation cocktail. Then, the cells were stained with anti-CD3-PerCPcy5.5, anti-IFN-γ-PEcy7, and anti-CD8-APCcy7 antibodies according to the protocol of the Fixation/Permeabilization Kit (BD Biosciences) and analyzed.

### Statistics

Statistical significance was determined using Prism 9.1 software (GraphPad). Unpaired *t* test with Welch’s correction, one-way ANOVA with the Dunnett T3 posttest, or two-way ANOVA with Tukey posttest was used to evaluate the significance of differences between 2 or more groups. All data are the mean ± SD.

## Results

### Recombinant IL-33 increases the population of CD103^+^ cDC1s in the spleen of IL-33-treated mice, which induces strong antitumor immunity

**Fig. 1:**
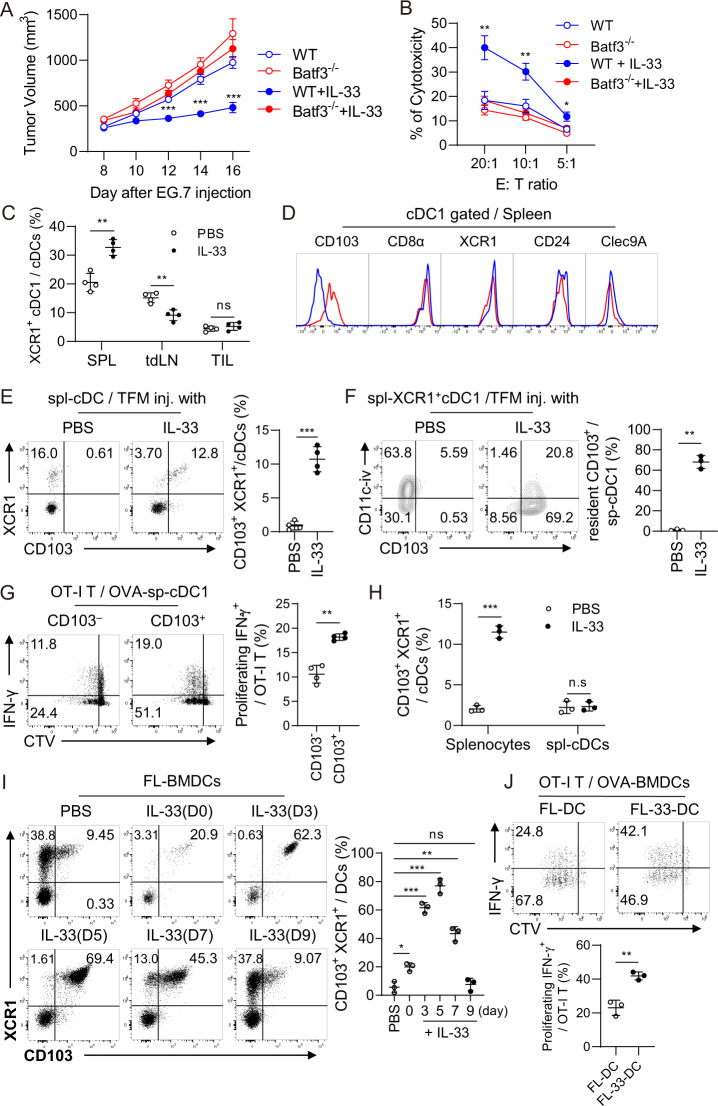
Recombinant IL-33 inhibits tumor growth by inducing spleen-resident CD103^+^ cDC1s and strong antitumor immunity. **A** Tumor growth was monitored in WT and Batf3^−/−^ EG7 TB mice treated with or without IL-33. IL-33 (1 µg) was *i.p*. inoculated daily for 6 days beginning on Day 8 after tumor injection. *n* = 4 per group. **B** CTL activity in the TILs of WT and Batf3^−/−^ EG7 TB mice with or without IL-33 injection was assessed. *n* = 4 per group. **C** The cDC1 ratio among cDCs in the spleens, tumor-draining lymph nodes (tdLNs), and TILs of PBS- or IL-33-treated TB mice. Representative FACS data are shown in Fig. S1C. *n* = 4 per group. **D** Expression of cDC1 markers in splenic cDC1s in IL-33-treated tumor-free mice (TFM). **E** Total splenic cDCs of WT and IL-33-treated TFM were further assessed for CD103^+^ cDC1s. *n* = 4 per group. **F** Intravascular staining of IL-33-treated mice with anti-CD11c Ab, followed by assessment of the tissue residency of splenic cDC1s. *n* = 3 per group. **G** CD103^+^ and CD103^–^ cDC1s isolated from the spleens of IL-33-treated mice were pulsed with OVA protein for 18 h and then cocultured with CTV-labeled OT-I T cells for 4 days. IFN-γ^+^ effector T-cell proliferation was assessed. *n* = 4 per group. **H** Total splenocytes and FACS-sorted splenic cDCs were cultured for 2 days in the presence of IL-33, and then CD103^+^ cDC1s were assessed. (Representative FACS data are shown in Fig. S1E) *n* = 3 per group. **I** During Flt3L-BMDC (FL-BMDC) generation, IL-33 (5 ng/ml) was added on the day indicated. Cells were harvested on Day 10, and CD103^+^ cDC1s were assessed. *n* = 3 per group. **J** FL-BMDCs generated in the presence (FL-33-DC) or absence of IL-33 (FL-DC) on Day 5 were harvested on Day 9, pulsed with OVA protein for 18 h, and then cocultured with CTV-labeled OT-I T cells for 4 days. IFN-γ^+^ effector T-cell proliferation was assessed. *n* = 3 per group. Two-way ANOVA with Tukey’s posttest (**A**, **B**), unpaired two-tailed Student’s *t* test with Welch’s correction (**C**, **E**–**H**, and **J**), and one-way ANOVA with Dunnett’s T3 posttest (**I**) were used to measure significance. ^*^P < 0.05, ^**^P < 0.01, ^***^P < 0.001; error bars indicate the mean ± SD

IL-33-mediated tumor suppression did not occur in Batf3^−/−^ mice (Fig. 1A). Tumor-specific cytotoxic T-cell (CTL) responses in the tumor were significantly enhanced in IL-33-OL-33 wild-type (WT) tumor-bearing (TB) mice but not in IL-33-treated Batf3^−/−^ TB mice (Fig. 1B). These data suggest that Batf3-dependent cDC1s play a critical role in the enhancement of antitumor immunity in IL-33-treated TB mice. In IL-33-treated TB mice, the cDC1 population increased significantly only in the spleen but decreased or remained unchanged in the tumor mass and tumor-draining lymph nodes (tdLNs) (Figs. 1C and S1A). The population of effector memory CD8^+^ T cells (Fig. S1B) and the proliferation of antigen-specific effector CD8^+^ T cells (Fig. S1C) were also significantly enhanced in the spleens of IL-33-treated TB WT mice but not in Batf3^−/−^ mice.

We next examined the surface phenotype changes of the splenic cDC1s from IL-33-treated tumor-free (TF) mice with typical cDC1 markers and found that CD103 expression was markedly enhanced, whereas the other cDC1 markers were minimally affected by recombinant IL-33 (Fig. 1D). CD103 expression was reconfirmed by spectral cytometry analysis (Fig. S1D). The population of CD103^+^ cDC1s was significantly enhanced in the spleens of IL-33-treated TF mice (Fig. 1E). Unlike in TB mice (Fig. 1C), the total cDC1 population in TF mice did not change in the spleen, even when IL-33 was administered (Fig. 1E). Interestingly, the majority of the IL-33-induced CD103^+^ cDC1s showed spleen residency, while normal splenic cDC1s were primarily migratory DCs (Fig. 1F). CD103^+^ cDC1s were more potent than CD103^-^ cDC1s in priming effector T cells (Fig. 1G). When total splenocytes were cultured in the presence of IL-33, the frequency of CD103^+^ cDC1s increased significantly compared with the untreated control; however, when purified splenic cDCs were cultured under the same conditions, CD103^+^ cDC1s were not properly induced (Fig. 1H, S1E). These results indicate that recombinant IL-33 might be involved in developing CD103^+^ cDC1s rather than driving additional CD103 expression from the established CD103^-^ cDC1s in the spleen. We investigated CD103 expression while adding IL-33 at different time points during the generation of Flt3-BMDCs (FL-DCs). The CD103^+^ cDC1 population increased significantly when IL-33 was added up to Day 5 and then decreased thereafter (Fig. 1I). This result supports our hypothesis that the population of CD103^+^ cDC1s is newly developed from DC precursors in the presence of IL-33 rather than from established splenic cDCs. IL-33-treated FL-DCs (FL-33-DCs) primed CD8^+^ T cells more potently than untreated FL-DCs (Fig. 1J). Taking these results together, we suggest that the enhanced antitumor immunity and tumor growth inhibition seen in IL-33-treated TB mice are due to a newly developed immunogenic population of CD103^+^ cDC1s in the spleen.

### IL-33-mediated CD103^+^ cDC1s are more potent than GM-CSF-mediated CD103^+^ cDC1s in priming antitumor immunity

**Fig. 2:**
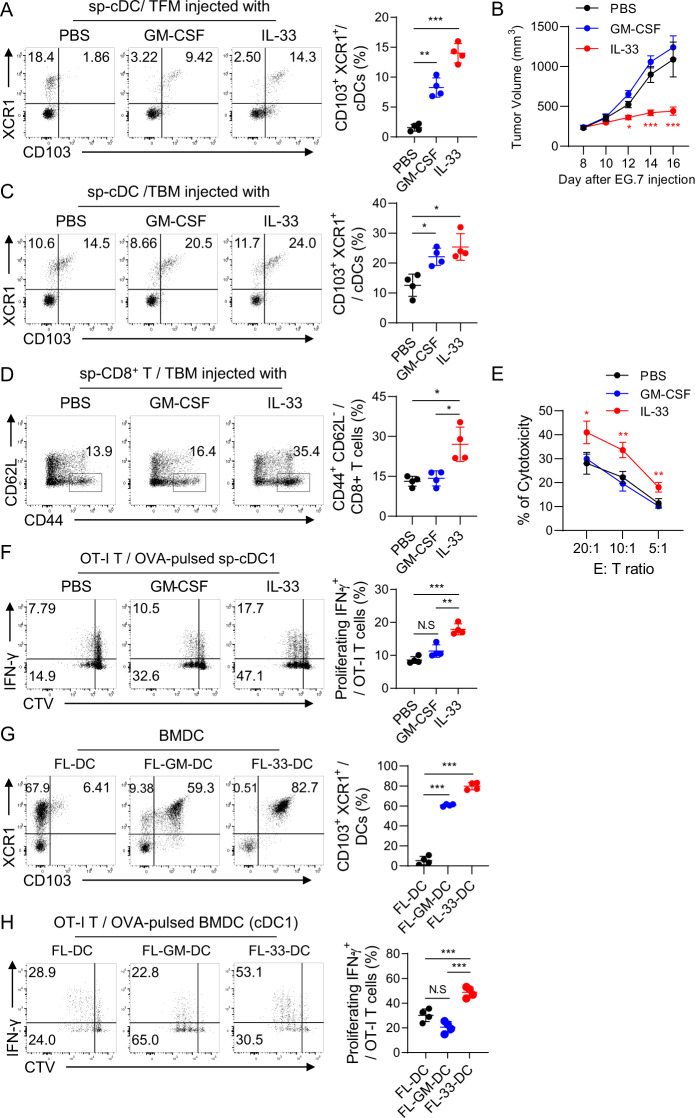
IL-33-mediated CD103^+^ cDC1s induce antitumor immunity more potently than GM-CSF-mediated CD103^+^ cDC1s. **A** CD103^+^ cDC1s were assessed on Day 9 in the spleens of tumor-free WT mice that were treated daily for 8 days with 1 µg/mouse GM-CSF or IL-33. *n* = 4 per group. **B** EG7 tumor growth was monitored in PBS-, GM-CSF-, and IL-33-treated mice. GM-CSF or IL-33 was *i.p*. inoculated daily for 6 days beginning on Day 8 after tumor injection. *n* = 4 per group. **C** CD103^+^ cDC1s were assessed in the spleens of PBS-, GM-CSF-, and IL-33-treated TB mice. *n* = 4 per group. **D** Activated CD8^+^ T cells were assessed in the spleens of PBS-, GM-CSF-, and IL-33-treated TB mice. *n* = 4 per group. **E** CTL activity of the TILs in PBS-, GM-CSF-, and IL-33-treated TB mice. *n* = 4 per group. **F** cDC1s isolated from the spleens of PBS-, GM-CSF-, and IL-33-treated mice were pulsed with OVA protein and then cocultured with CTV-labeled OT-I T cells for 4 days. The proliferating IFN-γ^+^ CD8^+^ T-cell population was assessed. *n* = 3 per group. **G** During FL-BMDC generation, GM-CSF or IL-33 was administered on Day 5, and then the cells were collected on Day 10. CD103^+^ cDC1s were assessed in the control (FL-DCs), GM-CSF (FL-GM-DCs), and IL-33-treated FL-BMDCs (FL-33-DCs). *n* = 4 per group. **H** Each group of BMDCs was pulsed with OVA protein for 18 h. cDC1s were isolated and cocultured with CTV-labeled OT-I T cells for 4 days. The proliferating IFN-γ^+^ CD8^+^ T-cell population was assessed. *n* = 4 per group. Unpaired one-way ANOVA with Dunnett’s T3 posttest (**A**, **C**, **D**, **F**, **G**, **H**) and two-way ANOVA with Tukey’s posttest (**B**, **E**) were used to measure significance. ^*^P < 0.05, ^**^P < 0.01, ^***^P < 0.001; error bars indicate the mean ± SD

As reported previously [48, 57], GM-CSF also induced the generation of CD103^+^ DC1s in the spleens of TF mice (Fig. 2A). However, unlike recombinant IL-33, recombinant GM-CSF did not inhibit tumor growth in the EG7 T-cell lymphoma model **(**Fig. 2 B) and TC-1 tumor model (Fig. S2A) in TB mice; instead, it promoted tumor growth in the B16F10 tumor model (Fig. S2B). The GM-CSF-treated TB mice also showed an increased population of CD103^+^ cDC1s, similar to the IL-33-treated TB mice, in the spleen (Fig. 2C). Although the total cDC population was significantly reduced in the spleens of both the GM-CSF- and IL-33-treated TB mice (Fig. S2C), the frequency of cDC1s among the cDCs in the spleen was higher in the IL-33-treated mice than in the GM-CSF-treated mice, and the cDC2 frequency was reversed (Fig. S2D). We next examined T-cell immunity in the IL-33- and GM-CSF-treated TB mice and found that the frequency of effector CD8^+^ cells in the spleen and CTL activity in tumor-infiltrating lymphocytes (TILs) increased significantly in the IL-33-treated mice but not the GM-CSF-treated mice (Fig. 2D-E). As previously reported [27–30], the Treg population also increased significantly in the spleens of IL-33-treated mice but not GM-CSF-treated mice (Fig. S3A). These results suggest that the strong antitumor immunity derived from the IL-33-induced CD103^+^ cDC1 population is sufficient to overcome IL-33-induced Treg-mediated inhibition. Recently, tumor-specific Tc9 cells were reported in mice treated with IL-33-treated GM-CSF-induced BMDCs [46]. However, we could not detect a population of Tc9 cells in the spleens of our IL-33-treated TB mice (Fig. S3B).

Splenic cDC1s isolated from IL-33-treated mice were more potent than those from GM-CSF-treated mice in priming antigen-specific effector CD8^+^ T cells (Fig. 2F). We generated FL-33-DCs and FL-GM-DCs by adding IL-33 and GM-CSF, respectively, on Day 5 during Flt3L-BMDC culture, as demonstrated in Fig. 1I. CD103^+^ cDC1s were the major population in both FL-33-DCs and FL-GM-DCs (Fig. 2G), as shown in the spleens of mice in Fig. 2A. When they were cocultured with OT-1 T cells, the FL-33-DCs were much more potent than the FL-GM-DCs in antigen-specific T-cell priming (Fig. 2H). These results suggest that recombinant IL-33-induced CD103^+^ cDC1s, the major population of cDC1s, are more potent than GM-CSF-induced CD103^+^ cDC1s in inducing the antitumor immunity of IFN-γ^+^ CD8^+^ T cells in mice. In the analysis of surface phenotype (MHC-1 and CD40) (Fig. S4A), IL-12 expression (Fig. S4B) and antigen uptake capacity (Fig. S4C), CD103^+^ cDC1s of FL-33-DCs were noticeably more immunogenic than were FL-GM-DCs or CD103- control FL-DCs. However, cell viability did not clearly differ among the cDC1s tested (Fig. S4D). Interestingly, over 60% of the IL-33-induced CD103^+^ cDC1s showed spleen residency, whereas the majority of the GM-CSF-derived cDC1s were migratory DCs (Fig. S4E). These results indicate that IL-33-induced CD103^+^ cDC1s differ from GM-CSF-induced cDC1s and are more potent than GM-CSF-induced cDC1s in T-cell priming and inducing antitumor immunity.

### Immunogenic CD103^+^ cDC1 development depends on extrinsic factors secreted from IL-33-primed ST2^+^ immune cells

**Fig. 3:**
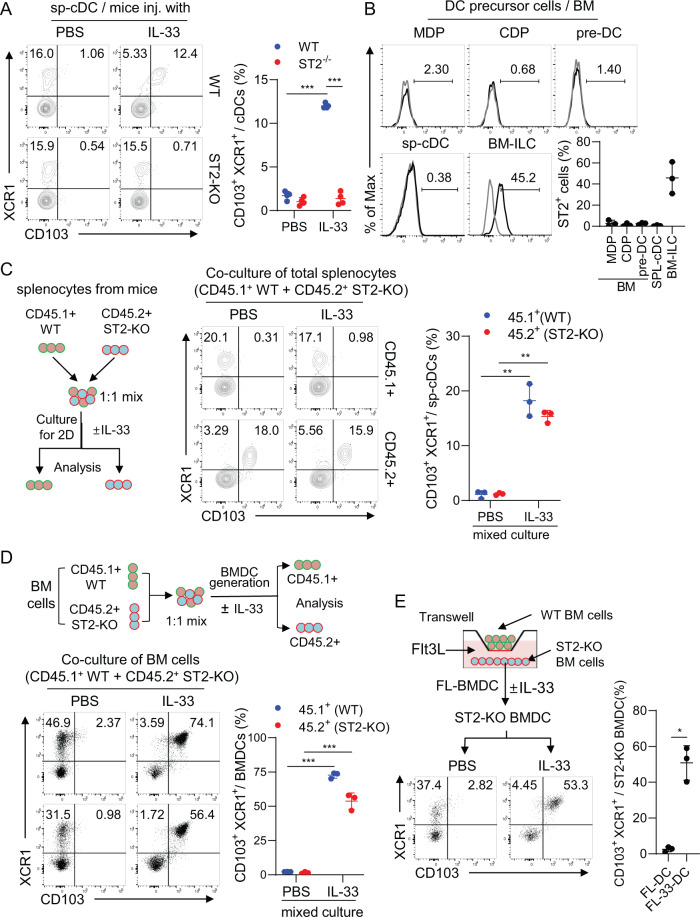
IL-33-mediated immunogenic CD103 + cDC1 development is attributed to extrinsic factors secreted by ST2+ bystander immune cells. **A** CD103^+^ cDC1s were assessed in the spleens of WT and ST2^−/−^ mice after injection with IL-33. *n* = 4 per group. **B** ST2 expression level in DC precursors from bone marrow (BM) and splenic cDCs, together with ILCs in BM as a positive control. *n* = 3 per group. **C** As illustrated, total splenocytes of WT (CD45.1^+^) and ST2^−/−^ (CD45.2^+^) mice were mixed at a 1:1 ratio and cultured in the presence of IL-33 for 2 days. CD103^+^ cDC1s were assessed in CD45.1^+^ cells and CD45.2^+^ cells. *n* = 3 per group. **D** As illustrated, WT (CD45.1^+^) and ST2^−/−^ (CD45.2^+^) BM cells were mixed at a 1:1 ratio and cultured in the presence of Flt3L. IL-33 was added on Day 5 of the culture, and the cells were collected on Day 10. CD103^+^ cDC1s were assessed in CD45.1^+^ and CD45.2^+^ cells. *n* = 3 per group. **E** WT Flt3L-BMDCs and ST2^−/−^ Flt3L-BMDCs on Day 5 of culture were placed in the upper and lower chambers of a transwell, respectively, and then cultured for an additional 5 days in the presence of IL-33. CD103^+^ cDC1s in the lower chamber were assessed. *n* = 3 per group. Unpaired two-way ANOVA with Tukey’s posttest (**A**, **C**, **D**) and unpaired two-tailed Student’s *t* test with Welch’s correction (**E**) were used to measure significance. ^*^P < 0.05, ^***^P < 0.001; error bars indicate the mean ± SD

ST2 is well-established as a receptor for IL-33 [1]. As expected, no CD103^+^ cDC1 population was detectable in the spleens of IL-33-treated ST2-deficient mice (ST2^−/−^ or ST2-KO) (Fig. 3A). The population was not induced in IL-33-treated ST2-KO splenocytes (Fig. S5A). The population of CD103^+^ cDC1s was also undetectable in FL-33-DCs when generated from ST2-KO mice (Fig. S5B). We examined ST2 expression in DC precursors and purified cDCs. Neither the cDC precursors in the BM nor splenic cDCs expressed ST2, unlike the BM ILCs used as a positive control (Fig. 3B). These data suggest that IL-33-induced CD103^+^ cDC1 development depends on extrinsic factors from other ST2^+^ immune cells rather than the IL-33/ST2 intrinsic signaling pathway of cDCs.

When ST2-KO mouse splenocytes were cocultured with WT splenocytes in the presence of IL-33, the population of CD103^+^ cDC1s developed efficiently from the ST2-KO splenocytes (Fig. 3C). As shown in mice (Fig. 3B), ST2 expression was not observed in the DC precursors at any developmental stage of FL-DCs (Fig. S5C). However, when WT and ST2-KO mouse BM cells were cocultured for FL-DC generation in the presence of IL-33, the population of CD103^+^ cDC1s was efficiently induced, even from the ST2-KO mouse BM cells (Fig. 3D). These results indicate that CD103^+^ cDC1 development in the spleens of IL-33-treated mice and during FL-33-DC generation depends on extrinsic factors derived from ST2^+^ bystander immune cells. When CD45.1^+^ WT and CD45.2^+^ ST2-KO BM cells were cocultured in a transwell system, CD103^+^ cDC1s were also equivalently induced in FL-33-DCs generated from the ST2-KO BM cells (Fig. 3E). In addition, a CD103^+^ cDC1 population was efficiently induced in ST2-KO FL-DCs when they were generated with culture supernatants from WT FL-33-DCs (Fig. S5D). These data suggest that soluble factors secreted by ST2^+^ bystander immune cells are involved in the development of IL-33-induced CD103^+^ cDC1s.

### IL-33-induced highly immunogenic CD103^+^ cDC1s are distinct from GM-CSF-induced CD103^+^ cDC1s in their gene expression patterns, particularly in the expression of FCGR3

**Fig. 4:**
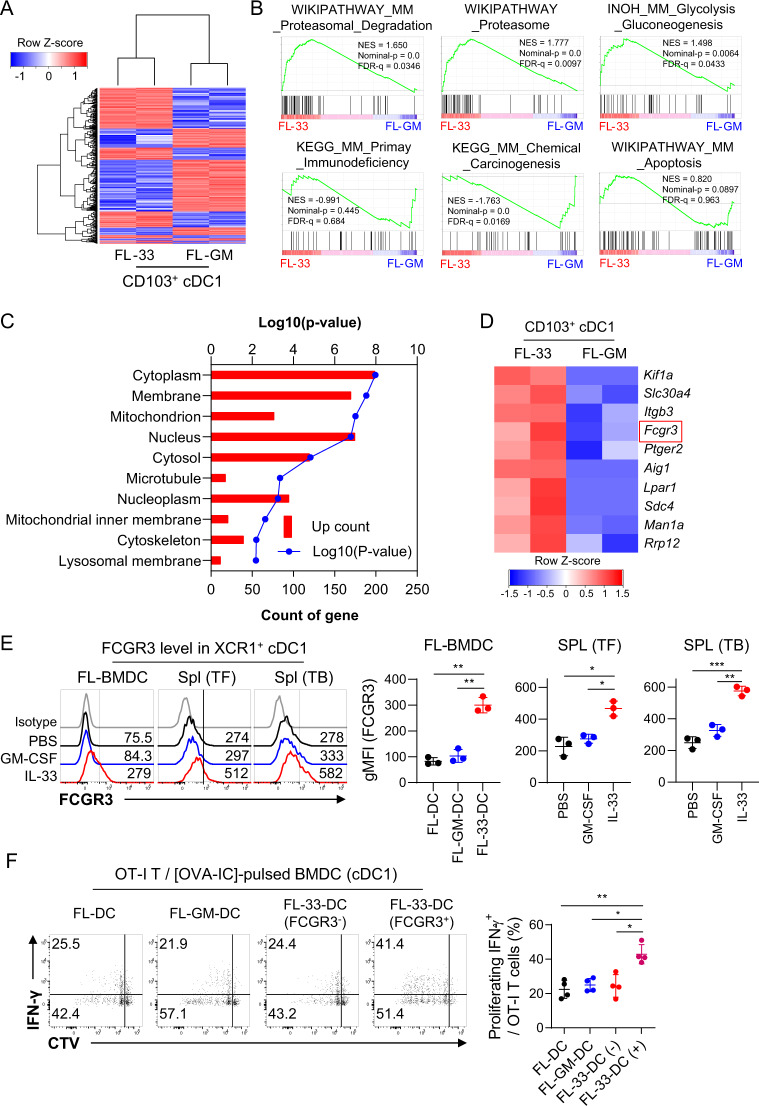
IL-33-induced immunogenic CD103 + cDC1s are distinct from GM-CSF-induced CD103 + cDC1s in the expression of FCGR3. **A** Heatmap of gene expression changes between CD103^+^ cDC1s from FL-33-DCs and FL-GM-DCs. Among 1076 differentially expressed genes, 524 were upregulated, and 552 were downregulated in FL-33-DCs. **B** Gene set enrichment analysis of FL-33-cDC1s vs. FL-GM-cDC1s. **C** Gene ontology of cellular component genes that were upregulated in FL-33-cDC1s vs. FL-GM-cDC1s (FC > 2 and *p* < 0.2). **D** Heatmap of the top 10 genes from the “Membrane” annotation. **E** FCGR3 expression in the cDC1s of PBS-, GM-CSF-, or IL-33-treated FL-BMDCs or spleens of tumor-free or tumor-bearing mice. *n* = 3 per group. **F** cDC1s isolated from FL-DCs and FL-GM-DCs and FCGR3^-^ or FCGR3^+^ cDC1s isolated from FL-33-DCs were treated with OVA-IC (mixture of OVA and anti-mouse OVA IgG1) for 18 h. OVA-IC-treated DCs were cocultured with OT-I T cells for 4 days. The proliferating IFN-γ^+^ CD8^+^ T-cell population was assessed. *n* = 4 per group. Unpaired one-way ANOVA with Dunnett’s T3 posttest (**E**, **F**) was used to measure significance. ^*^P < 0.05, ^**^P < 0.01; error bars indicate the mean ± SD

To assess genotypic differences between IL-33-induced CD103^+^ cDC1s and GM-CSF-induced CD103^+^ cDC1s, we performed RNA sequencing of CD103^+^ cDC1s isolated from FL-33-DCs and FL-GM-DCs. Although the expression patterns for general cDC markers and costimulatory molecules did not differ significantly between the CD103^+^ cDC1s of FL-33-DCs and FL-GM-DCs (Fig. S6A, B), IL-33-induced CD103^+^ cDC1s were distinct from GM-CSF-derived CD103^+^ cDC1s, with 1076 differentially expressed genes ( | log_2_ fold change | ≥ 1, *p* value < 0.2) (Fig. 4A). In a gene set enrichment analysis (GSEA), the gene sets of proteasome [58] and glycolysis/gluconeogenesis [59, 60] related to the activation or cross-priming of DCs were positively enriched in the IL-33-induced CD103^+^ cDC1s compared with the GM-CSF-induced CD103^+^ cDC1s, whereas the gene set of primary immunodeficiency was negatively enriched in the IL-33-induced CD103^+^ cDC1s (Fig. 4B). An in-depth study revealed an increase in the expression of *Psmb3, Psmd6*, and *Psmg1* associated with the proteasome and *Hk3* and *Pfkfb2* associated with glycolysis/gluconeogenesis in both OVA antigen-pulsed and unpulsed IL-33-derived CD103^+^ cDC1s (Fig. S7A). Taken together, these data, support our findings that IL-33-induced CD103^+^ cDC1s have more potent immunogenicity than GM-CSF-induced cDC1s. To identify specific surface marker(s) for IL-33-induced CD103^+^ cDC1s, we analyzed the top enriched terms for cellular components in the gene ontology enrichment of 524 upregulated genes and found that a “Membrane” annotation related to the cell surface appeared at a high frequency in the DAVID Bioinformatics Resources analysis (Fig. 4C). Among the top 10 upregulated genes investigated in the “Membrane” annotation, we selected *Fcgr3* as a candidate IL-33-induced cDC1 marker (Fig. 4D) because in the DAVID Bioinformatics Resources, FCGR3 has immunogenic characteristics, such as phagocytosis, recognition, positive regulation of tumor necrosis factor production, and antigen processing/presentation of exogenous peptide antigen via MHC class I (https://david.ncifcrf.gov/annotationReport.jsp?annot=86,91,92,78,27,35,43,52,50,73,89&currentList=0). FCGR3 was also expressed on the surface of cDC1s of in vitro-generated FL-33-DCs and in vivo splenic cDC1s of IL-33-injected mice, regardless of tumor-bearing condition (Fig. 4E**)**, but was undetectable in any DC subsets of FL-DCs or FL-GM-DCs **(**Fig. S7B). FCGR3, CD61 (encoded by *Itgb3*), and CD38 were also expressed on the surface of cDC1s only in FL-33-DCs (Fig. S8). Antigen pulsing did not greatly affect the expression patterns of these DEGs that were upregulated in FL-33-DCs (Fig. S7A and Fig. S8). FCGR3 is known as a member of the activating Fcγ receptor family, whose expression on DCs can respond to the immune complex [61]. To evaluate the T-cell priming capacity of FCGR3^+^ cDC1s in FL-33-DCs, FCGR3^+^ and FCGR3^-^ cDC1 cell fractions were treated with OVA antigen-antibody immune complex (IC) and cocultured with OT-I T cells. FCGR3^+^ cDC1s were much more potent than the FCGR3^-^ cDC1s of FL-33-DCs or FL-GM-DCs in antigen-specific T-cell priming capacity (Fig. 4F). Our findings suggest that IL-33-induced CD103^+^ cDC1s exhibit clear differences from GM-CSF-induced CD103^+^ cDC1s in terms of their gene expression profiles and T-cell priming capacity. Moreover, FCGR3 can be used as a discernable marker for IL-33-induced highly immunogenic CD103^+^ cDC1s.

### IL-33-primed ST2^+^ basophils play a crucial role in the development of highly immunogenic FCGR3^+^CD103^+^ cDC1s

**Fig. 5:**
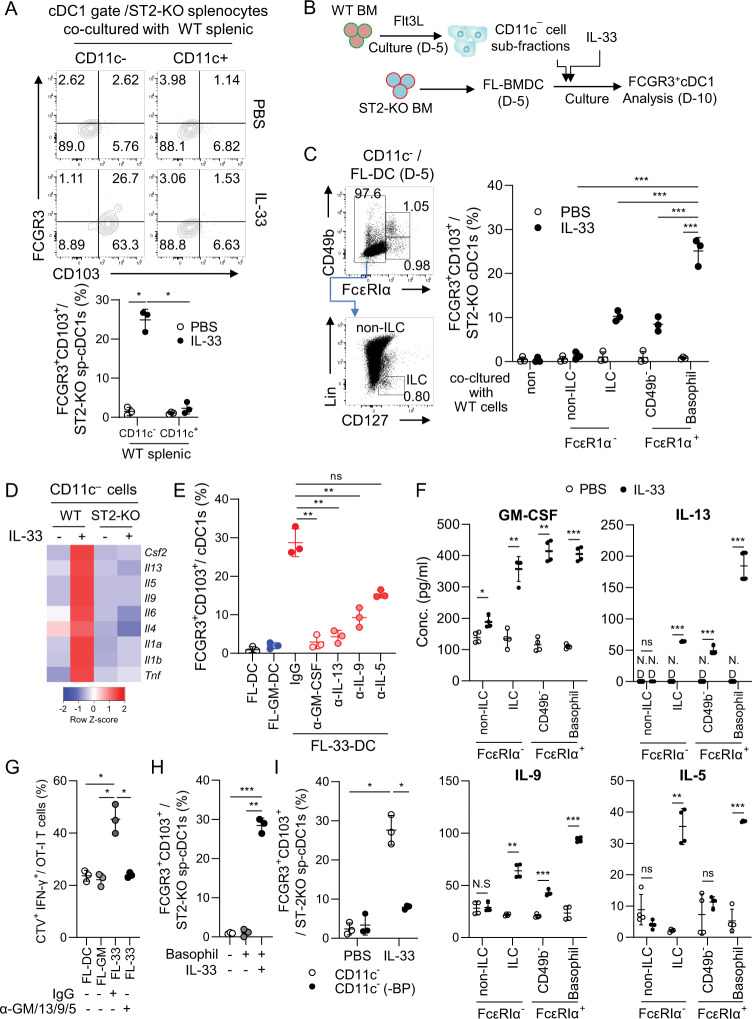
ST2^+^ basophils play a key role in the development of immunogenic FCGR3^+^CD103 + cDC1s via cytokines secreted upon activation by recombinant IL-33. **A** ST2-KO splenocytes were cocultured with CD11c^–^ or CD11c^+^ cells from WT splenocytes for 2 days in the presence or absence of IL-33. FCGR3 + CD103 + cDC1s were assessed in each culture. *n* = 3 per group. **B** Schematic illustration of the experiments. **C** CD11c^–^ cells on Day 5 of FL-DC culture were divided into FcεRIα^–^ and FcεRIα^+^ cells. The FcεRIα^+^ cells were further fractionated into CD49b^–^ cells and CD49b^+^ basophils, and the FcεRIα^–^ cells were also fractionated into CD127^–^ non-ILCs and CD127^+^ ILCs (left). These cell fractions (cell number normalized) were mixed with ST2-KO FL-DCs on Day 5 and then were cocultured in the presence of IL-33 for an additional 5 days. Then, CD103^+^ FCGR3^+^ cDC1s were assessed. (Representative FACS data are shown in Fig. S9B) *n* = 3 per group. **D** CD11c^–^ cells from FL-DCs on Day 5 of culture were isolated and treated with IL-33 for 6 h. The cells were harvested, and RNA sequencing was performed. The heatmap shows cytokine genes that were differentially expressed by IL-33 between CD11c^–^ cells from WT and ST2-KO FL-DCs. **E** FL-33-DCs were generated in the presence of the neutralizing antibody against each cytokine (added on Day 5), and then the frequency of CD103^+^ FCGR3^+^ cDC1s was assessed on Day 10. (Representative FACS data are shown in Fig. S10A) *n* = 3 per group. **F** Non-ILCs and ILCs among FcεRIα^-^ cells and CD49b^–^ cells and CD49b^+^ basophils among FcεRIα^-^ cells were isolated from the culture of FL-DCs on Day 5. Cell fractions were treated with IL-33 for 36 h, and the level of each cytokine in the culture supernatant was examined by ELISA. *n* = 4 per group. **G** FL-DCs, FL-GM-DCs, and FL-33-DCs generated in the presence or absence of cytokine-neutralizing antibody mixtures were pulsed with OVA protein for 18 h. cDC1s were isolated and cocultured with CTV-labeled OT-I T cells for 4 days. The proliferating IFN-γ^+^CD8^+^ T-cell population was assessed. (Representative FACS data are shown in Fig. S10C) *n* = 3 per group. **H** ST2-KO splenocytes were cocultured with basophils from WT splenocytes for 2 days in the presence or absence of IL-33. FCGR3^+^CD103^+^ cDC1s were assessed. (Representative FACS data are shown in Fig. S11A) *n* = 3 per group. **I** ST2-KO splenocytes were cocultured with total or basophil-depleted (ΔBP, by FACS sorting) CD11c^–^ cells from WT splenocytes for 2 days in the presence or absence of IL-33. FCGR3^+^CD103^+^ cDC1s were assessed. (Representative FACS data are shown in Fig. S11B) *n* = 3 per group. Two-way ANOVA with Tukey’s posttest (**A**, **C**, **I**), one-way ANOVA with Dunnett’s T3 posttest (**E**, **G**, **H**), and unpaired two-tailed Student’s *t* test with Welch’s correction (**F**) were used to measure significance. ^*^P < 0.05, ^**^P < 0.01, ^***^P < 0.001; error bars indicate the mean ± SD

Using FCGR3 as a phenotypic marker for highly immunogenic CD103^+^ cDC1s, we investigated the bystander immune cells and extrinsic soluble factors required for the development of IL-33-induced highly immunogenic FCGR3^+^CD103^+^ cDC1s. We cocultured ST2-KO splenocytes with cell fractions of WT splenocytes. When the CD11c^-^ fraction of WT splenocytes was used for the coculture, the population of FCGR3^+^CD103^+^ cDC1s was significantly induced in IL-33-treated ST2-KO splenocytes but was not induced at all when the CD11c^+^ fraction was used (Fig. 5A). Similar results were found in transwell-based BMDC generation. The population of FCGR3^+^CD103^+^ cDC1s was efficiently generated from ST2-KO FL-33-DC cultures only when the CD11c^–^ fraction of WT FL-DC cultures on Day 5 was placed in the upper chamber of the Transwell culture (Fig. S9A). These data suggest that IL-33-derived extrinsic factors necessary for the development of FCGR3^+^CD103^+^ cDC1s originate from the fraction of CD11c^–^ cells. To identify the bystander immune cells in that CD11c^–^ fraction, we generated FL-33-DCs from ST2-KO BM cells in the presence of CD11c^–^ subfractions taken from WT FL-DCs on Day 5, as illustrated in Fig. 5B. We divided the CD11c^–^ cells into FcεRIα^–^ and FcεRIα^+^, the FcεRIα^-^ cells into CD127^+^ ILCs and CD127^-^ non-ILCs [16], and the FcεRIα^+^ cells into CD49^+^ basophils and the CD49b^–^ portion (Fig. 5C left). Among those CD11c^–^ subfractions taken from WT FL-DC cultures on Day 5, the FcεRIα^+^ CD49b^+^ basophils were the most potent, and the FcεRIα^–^CD127^+^ ILC2 and FcεRIα^+^ CD49^–^ mast cell fractions were partially involved in the generation of FCGR3^+^CD103^+^ cDC1s from ST2-KO FL-33-DC culture (Fig. 5C right, Fig. S9B). On the other hand, IL-33-related cytokine genes, such as *Csf2, Il13, Il9*, and *Il5*, increased significantly in IL-33-primed WT CD11c^–^ cells but not in ST2-KO CD11c^–^ cells in a transcriptomic analysis (Fig. 5D). When IL-33-related cytokines were blocked, FCGR3^+^CD103^+^ cDC1 development in FL-33-DC cultures was significantly impaired, mainly by blocking GM-CSF and IL-13 and partially by blocking IL-9 and IL-5 (Fig. 5E, Fig. S10A). To investigate the time difference in the expression of CD103 and FCGR3 during FL-33-DC culture, we performed a time kinetics study of FL-33-DC culture. In this study, it was found that CD103 expression precedes the expression of FCGR3 in the development of cDC1s during FL-33-DC cultures (Fig. S10B). In addition, treatment of the FL-33-DC culture with anti-GM-CSF Ab inhibited the expression of both FCGR3 and CD103, while treatment with anti-IL-13 Ab inhibited the expression of FCGR3 but had no effect on CD103 expression (Fig. S10B). This finding indicates that GM-CSF is involved in the expression of CD103, while IL-13 plays a crucial role in the additional expression of FCGR3 in CD103^+^ cDC1s during FL-33-DC cultures. Among the CD11c^–^ cell fractions, basophils had the most prominent expression of these cytokines, especially IL-13 (Fig. 5F). The T-cell priming capacity of FL-33-DCs was completely abolished by blocking those cytokines during the generation of FL-33-DCs (Fig. 5G, Fig. S10C). In the culture of DC precursors on the ST2-KO feeder cells, FCGR3^+^CD103^+^ cDC1s were induced as efficiently as in FL-33-DC culture from all DC precursors when cultured with IL-33-treated WT basophils, but the effect was not observed with IL-33 or basophils alone (Fig. S11). When the IL-33-treated basophil-derived cytokines were additionally blocked, the development of FCGR3^+^CD103^+^ cDC1s from DC precursors was significantly impaired, mainly by blocking GM-CSF and IL-13 and partially by blocking IL-9 and IL-5 (Fig. S11), as shown in FL-33-DCs (Fig. S10A). In ex vivo culture experiments, the population of FCGR3^+^CD103^+^ cDC1s was also significantly induced in ST2-KO splenocytes when cultured with IL-33-treated WT basophils (Fig. 5H, Fig. S12A). On the other hand, when ST2-KO splenocytes were cocultured with basophil-depleted CD11c^–^ cells of WT splenocytes in the presence of IL-33, the frequency of FCGR3^+^CD103^+^ cDC1s was significantly reduced (Fig. 5I, Fig. S12B). Taking these results together, we conclude that ST2^+^ basophils play a crucial role in the development of IL-33-induced immunogenic FCGR3^+^CD103^+^ cDC1s by secreting IL-33-derived cytokines (including GM-CSF, IL-13, IL-9, and IL-5) as extrinsic soluble factors.

It was recently reported that IL-33 activates tumoral ILC2s that double the abundance of intratumoral CD103^+^ DCs, priming and recruiting CD8^+^ T cells into orthotopic pancreatic tumors to restrict tumor growth in mice [62]. Considering the results shown in Fig. 5C and Fig. 5F, we wondered whether ILC2s would be involved at least in part in the development of FCGR3^+^CD103^+^ cDC1s in FL-33-DCs. The ILC2 frequency was less than 0.3% of the total live cells during the entire culture of FL-DCs (Fig. S13A) and was expanded 2 ~ 3 times by IL-33 in FL-33-DC cultures (Fig. S13B). However, ILC2 depletion in the FL-33-DC cultures by adding anti-CD90 antibody (Fig. S13C) did not greatly affect the development of CD103^+^ FCGR3^+^ cDC1s (Fig. S13D) or the CD8^+^ T-cell priming capacity of FL-33-DCs (Fig. S13E). These results suggest that ILC2s might not greatly affect the IL-33-mediated development of CD103^+^FCGR3^+^ cDC1s.

### FL-33-DCs were more potent than untreated FL-DCs or FL-GM-DCs in DC-based tumor immunotherapy

**Fig. 6:**
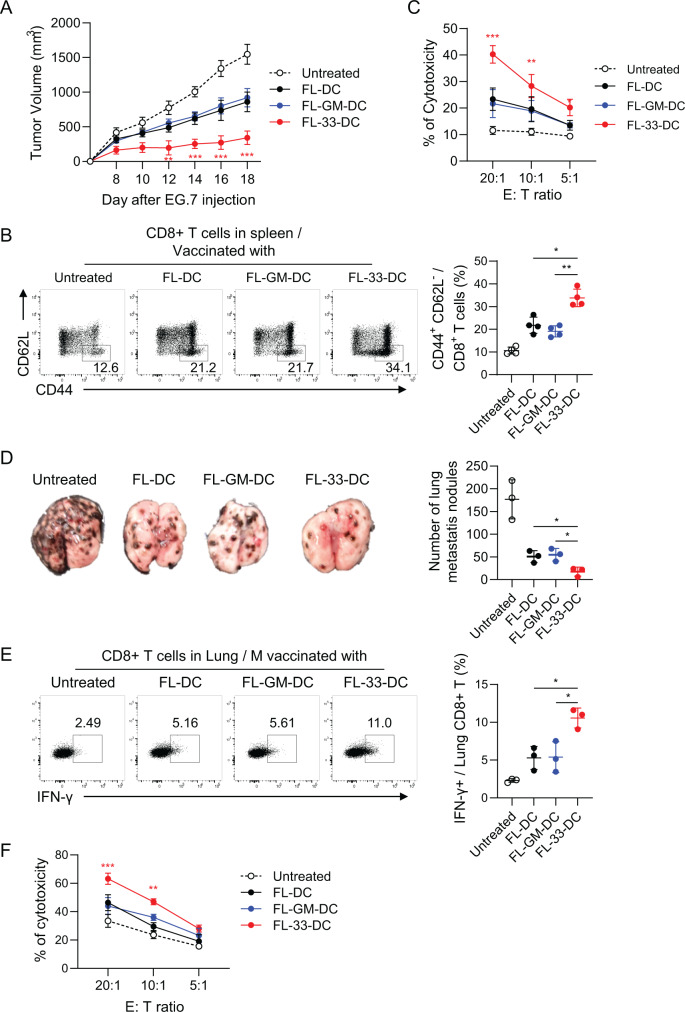
FL-33-DCs were much more potent than FL-DCs or FL-GM-DCs in tumor immunotherapy. **A** Tumor growth in EG7 TB mice treated with each BMDC vaccine. *n* = 4 per group. **B** Effector CD8 + T cells in the spleens of vaccinated mice. *n* = 4 per group. **C** CTL activity in the TILs of vaccinated mice. *n* = 4 per group. **D** Mice *i.v*. treated with B16F10 tumor cells were treated with each DC vaccine. Representative lung metastatic tumors (left) and the number of tumor nodules were examined. *n* = 3 per group. **E** IFN-γ+ effector CD8 + T cells in the lungs of vaccinated mice. *n* = 3 per group. **F** CTL activity in the lungs of vaccinated mice. *n* = 3 per group. Unpaired two-way ANOVA with Tukey’s posttest (**A**, **C**, **F**) and one-way ANOVA with Dunnett’s T3 posttest (**B**, **D**, **E**) were used to measure significance. ^*^P < 0.05, ^**^P < 0.01, ^***^P < 0.001; error bars indicate the mean ± SD

In DC-based tumor immunotherapy, the FL-33-DC vaccine inhibited tumor growth more potently than the other DC vaccine candidates in TB mice in the EG7, TC-1, and LLC tumor models (Fig. 6A, Fig. S14). CCR7 expression (Fig. S15A) and chemotactic migration capacity (Fig. S15B) were increased significantly in FL-33-DCs but were downregulated in FL-GM-DCs. Furthermore, the frequency of DC migration to the tdLNs was also higher in FL-33-DCs than in FL-DCs or FL-GM-DCs following DC vaccine inoculation (Fig. S15C). Effector CD8^+^ T cells **(**Fig. 6B) and Ag-specific CTL responses (Fig. 6C) were significantly enhanced in the spleens of mice vaccinated with FL-33-DCs compared with those vaccinated with other BMDCs. The mice vaccinated with FL-33-DCs showed a significant reduction in the number of tumor nodules compared with the other DC-vaccinated groups (Fig. 6D). IFN-γ^+^ effector CD8^+^ T cells and CTL responses in the lung were also significantly enhanced in mice vaccinated with FL-33-DCs compared with the other vaccinated mice (Fig. 6E, F). These data suggest that the FL-33-DC vaccine was more effective in inducing antitumor immunity and tumor immunotherapy than standard FL-DCs.

### Human monocyte-derived DCs (hMo-DCs) showed enhanced immunogenic phenotypes when cultures were treated with supernatants from IL-33-primed PBMC cultures

**Fig. 7:**
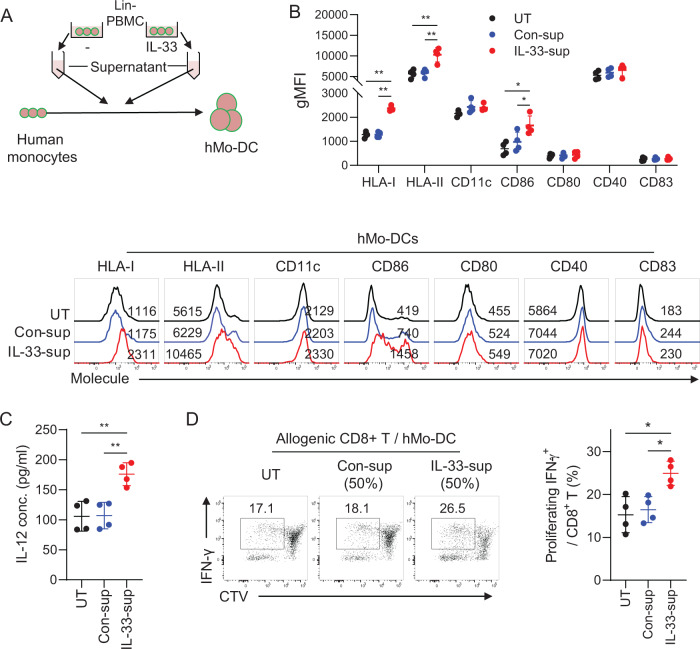
Enhanced immunogenic phenotype of human Mo-DCs generated in the presence of IL-33-activated basophil factors. **A** Schematic figure of human Mo-DC generation in the presence of culture supernatant derived from IL-33-treated Lin^-^ human PBMCs. **B** Surface phenotype of the Mo-DCs generated in (**A**). *n* = 4 per group. **C** IL-12 levels in the culture supernatants from control and supernatant-treated Mo-DCs with poly I:C. *n* = 4 per group. **D** IFN-γ + T cells in CD8 + T cells proliferated by coculturing with human Mo-DCs. *n* = 4 per group. Unpaired two-way ANOVA with Tukey’s posttest (**B**) and one-way ANOVA with Dunnett’s T3 posttest (**C**, **D**) were used to measure significance. ^*^P < 0.05, ^**^P < 0.01, ^***^P < 0.001; error bars indicate the mean ± SD

To determine whether the IL-33-mediated enhancement of DC immunogenicity could be employed in generating a human DC vaccine, we applied supernatants from IL-33-treated Lin^–^ PBMC (CD3/CD14/CD16/CD19/CD56/CD66b-depleted) cultures to generate hMo-DCs, as illustrated in Fig. 7A. Neither monocytes nor Mo-DCs in any culture stage expressed ST2, but the Lin^–^ cells did express ST2 (Fig. S16). IL-33-primed Lin^–^ cell supernatants significantly increased the expression of MHC I, MHC II, and the costimulatory molecule CD86 (Fig. 7B). In addition, hMo-DCs treated with supernatants from IL-33-primed PBMCs secreted more IL-12 than the untreated control (Fig. 7C). hMo-DCs treated with IL-33-primed PBMC supernatants also primed and activated allogenic CD8^+^ T cells more potently than those treated with control supernatants (Fig. 7D). These data suggest that IL-33-induced extrinsic factors secreted by ST2^+^ bystander immune cells such as basophils increase the immunogenicity of hMo-DCs.

## Discussion

This work was initiated with a finding that the cDC1 population increased significantly only in the spleens of IL-33-treated TB mice in which tumor growth was profoundly suppressed. However, the population of cDC1s in the tdLNs decreased in the same IL-33-treated TB mice **(**Fig. 1C**)**. This reduction in tdLN cDC1s might be attributed to the cytokines induced by IL-33, such as GM-CSF and IL-13, which are known to inhibit the expression of CXCR4, an essential chemokine receptor for DC migration into LNs [63–65]. Therefore, we postulated that migratory cDC1s might have exited the tdLNs due to the absence of CXCR4 expression in the IL-33-treated mice.

In both TB and TF mice, the major population of splenic cDC1s in IL-33-treated mice was CD103^+^ cDC1s that were barely detected in the spleens of normal mice. Splenic CD103^+^ cDC1s showed clear spleen residency and were more potent than CD103^–^ cDC1s in priming effector CD8^+^ T cells. Those CD103^+^ cDC1s were differentiated from DC precursors rather than by driving additional CD103 expression from fully developed CD103^–^ cDC1s (Fig. 1).

In Batf3^−/−^ mice lacking cDC1s, the population of CD8^+^ T cells partially increased in the presence of IL-33 **(**Fig. S1C**)**. However, even in Batf3^−/−^ mice lacking cDC1s, it has been reported that IL-33 can promote CD8^+^ T-cell responses directly or through CD11b^+^ DCs [43, 44] or via stimulation of other APCs, such as basophils [66, 67]. Nevertheless, the increased population of CD8^+^ T cells observed in IL-33-treated Batf3^−/−^ mice was not effective enough to suppress tumor growth in Batf3^−/−^ TB mice, as previously reported [41, 62]. This finding suggests that Batf3-dependent cDC1s are essential for IL-33-mediated antitumor immunity.

As previously reported [48, 57], GM-CSF also induced CD103^+^ cDC1s in the spleen (Fig. 2A). However, GM-CSF injection did not inhibit tumor growth; rather, it promoted tumor growth in the B16F10 tumor model **(**Fig. S2B**)**. High doses of GM-CSF were reported to directly suppress the immune response through myeloid suppressor cells or to promote an immunosuppressive tumor microenvironment [68, 69]. In our study, neither GM-CSF-induced splenic CD103^+^ cDC1s nor FL-GM-DCs generated in vitro showed immunogenic characteristics similar to those of IL-33-induced CD103^+^ cDC1s (Fig. 2 and Fig. S4). Unlike IL-33-induced CD103^+^ cDC1s, GM-CSF-induced cDC1s did not exhibit spleen residency (Fig. S4). As a result, GM-CSF-induced CD103^+^ cDC1s did not promote CD8^+^ T-cell priming capacity, so tumor growth was not inhibited or was even promoted, unlike with IL-33.

It was reported that GM-CSF-derived BMDCs express ST2, and IL-33 directly stimulates GM-CSF-derived BMDCs through the IL-33/ST2 axis [23, 46]. However, we did not find ST2 expression in any cDCs or cDC precursors in mice or in any stages of DC precursors during in vitro FL-BMDC generation (Fig. 3), probably due to the different protocols for the generation of BMDCs. Even in the absence of ST2 expression in DC precursors, a CD103^+^ cDC1 population was clearly observed in mice upon IL-33 inoculation but not in ST2-KO mice (Fig. 3A). However, the CD103^+^ cDC1 population was reestablished in ST2-KO splenocytes when cocultured with WT splenocytes in the presence of IL-33 (Fig. 3C). We assumed that ST2^+^ bystander immune cells might be involved in CD103^+^ cDC1 development in the spleens of IL-33-treated mice.

Moreover, in the mixed culture of BM cells to generate FL-33-DCs from WT and ST2-KO mice, we observed a lower frequency of ST2-KO-derived cDC1s compared to that of WT-derived cDC1s **(**Fig. 3D). Previous studies have reported that the IL-33/ST2 axis acts as a positive regulator of hematopoietic stem/progenitor cell proliferation (HSPCs) [70] and that cDC1-biased HSPCs also exist [71]. Based on these reports, we hypothesize that the decreased frequency of cDC1s in ST2-KO-derived FL-33-DCs might be attributed to the impairment in cDC1-biased HSPCs under ST2-KO conditions. However, this mechanism remains unclear and requires further investigation.

To identify a distinguishable surface marker for further bystander immune cell study of the IL-33-derived CD103^+^ cDC1s, we investigated remarkable DEGs from the RNA transcriptomics data between FL-33-DCs and FL-GM-DCs. Initially, we identified 8 upregulated DEGs in the FL-33-DCs based on FDR < 0.05, but we could not find any membrane-annotated genes from these DEGs. Subsequently, we changed the cutoff threshold to *p* < 0.05 to increase the number of candidates and found 52 candidates of membrane-annotated genes among the upregulated DEGs. However, none of the DEG candidates selected in FL-33-DCs with *p* < 0.05 showed similar differential expression patterns in splenic CD103^+^ cDC1s of IL-33-treated mice, as assessed by qRT‒PCR and FACS analysis in FL-33-DCs. Therefore, we raised the *p* value threshold up to *p* < 0.2 as an alternative approach to broaden the range of DEG candidates. We repeated qRT‒PCR and FACS analysis on the top-down potential DEG candidates. Ultimately, we identified FCGR3 as a reliable marker for IL-33-induced CD103^+^ cDC1s, with high expression levels observed in both FL-33-DCs and splenic CD103^+^ cDC1s of IL-33-treated mice (Fig. 4 and S8).

FCGR3 (FcγRIII) is a member of the Fcγ receptor (FcgR) family, which is dominantly expressed in monocytes, macrophages, neutrophils, and NK cells. FCGR3 is involved in antibody-dependent cellular phagocytosis, antibody-dependent cellular cytotoxicity, immune cell activation, antigen presentation, and macrophage polarization [72–74]. Considering these reports, FCGR3 expression in IL-33-induced cDC1s supports our finding that IL-33-induced CD103^+^ cDC1s are highly immunogenic. In addition, OVA-IC-treated FCGR3^+^ portions in the CD103^+^ cDC1s from FL-33-DCs showed more immunogenicity through antigen-specific CD8^+^ T-cell priming capacity. The role of FCGR3 in the immunogenicity of IL-33-induced FCGR3^+^CD103^+^ cDC1s without the Ag-Ab immune complex remains to be investigated.

Using FCGR3 as an additional marker, we investigated ST2^+^ bystander immune cells that are involved in the development of IL-33-induced CD103^+^ cDC1s and found that ST2^+^ basophils and their secreted cytokines play a crucial role in the development of FCGR3^+^CD103^+^ cDC1s (Fig. 5). The ST2^+^ ILC2 and mast cell fractions were also found to be partially involved in the generation of FCGR3^+^CD103^+^ cDC1s (Fig. 5C), but the depletion of ILC2s during the generation of FL-33-DCs did not affect the frequency of FCGR3^+^CD103^+^ cDC1s (Fig. S13D). In addition, FCGR3^+^CD103^+^ cDC1s can be differentiated from DC precursor culture in the presence of both basophils and IL-33 (Fig. S11), and basophil-depleted splenocytes impaired the differentiation of FCGR3^+^CD103^+^ cDC1s (Fig. S12), suggesting that IL-33-primed ST2^+^ basophils would be enough to generate a population of FCGR3^+^CD103^+^ cDC1s.

Among the extrinsic factors that we discovered (Fig. 5F), GM-CSF was essential for the expression of CD103, and IL-13/IL-9/IL-5 was involved in the development of immunogenic potential and FCGR3 expression (Fig. S10A). However, we could not generate FCGR3^+^CD103^+^ cDC1 cells by culturing DC precursors with mixtures of these 4 cytokines, suggesting that some other cytokines are also required for the IL-33-mediated development of FCGR3^+^CD103^+^ cDC1 cells, which remains to be investigated.

It was recently reported that tumor-derived IL-33 increases FcεRIα^+^ ST2^+^ macrophages and TGF-β secretion, which promote IL-33 expression in the tumor, followed by invasive tumor progression [32]. However, we did not detect TGF-β^+^ FcεRIα^+^ ST2^+^ macrophages or IL-33 expression from the tumor in our IL-33-treated TB mice (data not shown), probably due to the different source of IL-33, that is, exogenous IL-33, in this study.

FL-33-DCs offered more effective tumor immunotherapy than FL-DCs (Fig. 6). Although the ideal is to generate immunogenic human cDC1s from CD34^+^ hematopoietic progenitor cells using IL-33-derived factors, the methods of cDC1 generation and purification for use as a DC vaccine are still not simple [75]. For this reason, we applied IL-33-derived factors to immunogenic hMo-DC generation and found that hMo-DCs generated in the presence of supernatants from IL-33-primed PBMCs also showed an improved immunogenic phenotype (Fig. 7). We attempted to isolate basophils from human PBMCs but failed due to the limited blood samples. Instead, we used (T/B/monocyte)-depleted Lin^-^ PBMCs for the experiments. It was reported that hMo-DCs derived from cryopreserved PBMCs did not affect DC function [56], so Mo-DCs can still be used as next-generation DC vaccines, and we anticipated that it would be possible to use this method for next-generation DC vaccines. FCGR3 expression was also examined in hMoDCs, but hMoDCs were all FCGR3^+^ regardless of the treatment with IL-33-derived factors. Discernable markers for hMo-DCs improved by IL-33-derived factors remain to be investigated.

In conclusion, recombinant IL-33 induces highly immunogenic, spleen-resident FCGR3^+^CD103^+^ cDC1s via bystander activation of ST2^+^ basophils. The population of FCGR3^+^CD103^+^ cDC1s effectively induced antitumor immunity in IL-33-treated mice, leading to the inhibition of tumor progression. An IL-33-derived DC vaccine-induced antitumor immunity more potently than conventional vaccines. These results suggest that recombinant IL-33 or IL-33-mediated DC vaccines could be an attractive protocol to improve tumor immunotherapy.

## Supplementary information

Supplemental information

Original Image (Fig. S16)

List of differentially expressed genes
