# Supplementary material for: Discovery of highly immunogenic spleen-resident FCGR3+CD103+ cDC1s differentiated by IL-33-primed ST2+ basophils
Source: Cell Mol Immunol. 2023 May 29;20(7):820–34. doi: 10.1038/s41423-023-01035-8 (PMC10310784; doi:10.1038/s41423-023-01035-8)
Supplement: Supplementary file 1 — Supplemental information [file 41423_2023_1035_MOESM1_ESM.docx]

**Supplemental Figures**

**
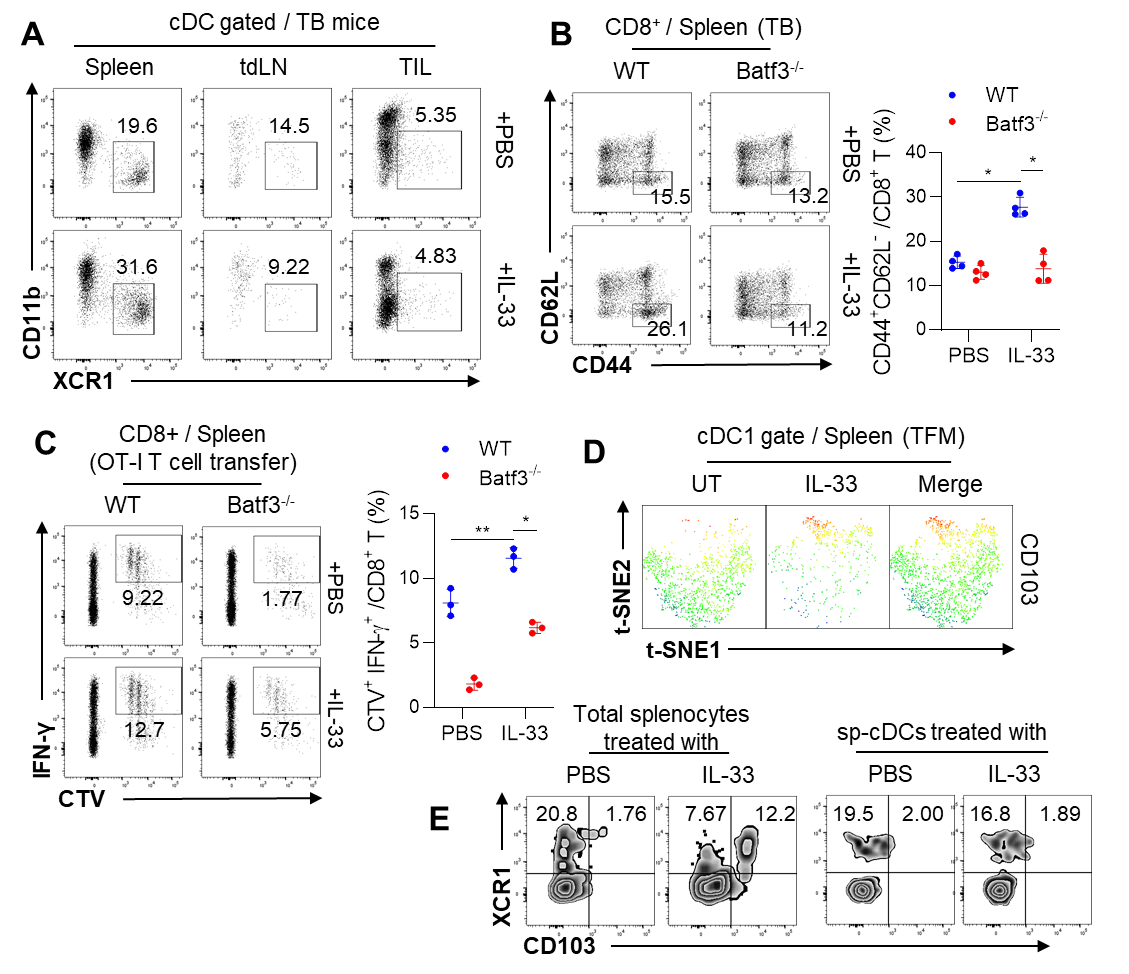
**

**Fig. S1. IL-33-mediated anti-tumor immunity was dependent on Batf3-mediated cDC1s and the expression of CD103 in the cDC1s.**

(A) cDC1 population assessed in the spleen, tumor draining lymph node (tdLN), and TIL of IL-33/PBS- injected tumor bearing mice. (B) CD44^+^CD62L^-^ effector memory CD8^+^ T cells were assessed in the spleen of WT or Batf3^-/-^ EG.7 tumor-bearing mice when administered with IL-33 or not. n=4 per group. (C) CTV-labeled OT-I T cells were adoptively transferred into OVA-immunized WT or Batf3^-/-^ mice. CTV^+^ IFN-γ^+^ OT-1 T cells were assessed in the spleens of tested mice. n=3 per group. (D) t-SNE plot of spectral cytometry analysis of CD103 in the splenic cDC1s from WT and IL-33-injected mice. (E) Total splenocytes (left) and FACS-sorted splenic cDCs (right) were cultured for 2 days in the presence of IL-33, and then CD103^+^ cDC1s were assessed by FACS. Shown are representative FACS data for the statistical data in Figure 1H. Two-way ANOVA with Turkey for post-test (B, C) were used to measure significance. *P < 0.05, **P < 0.01; error bars indicate mean ± SD.


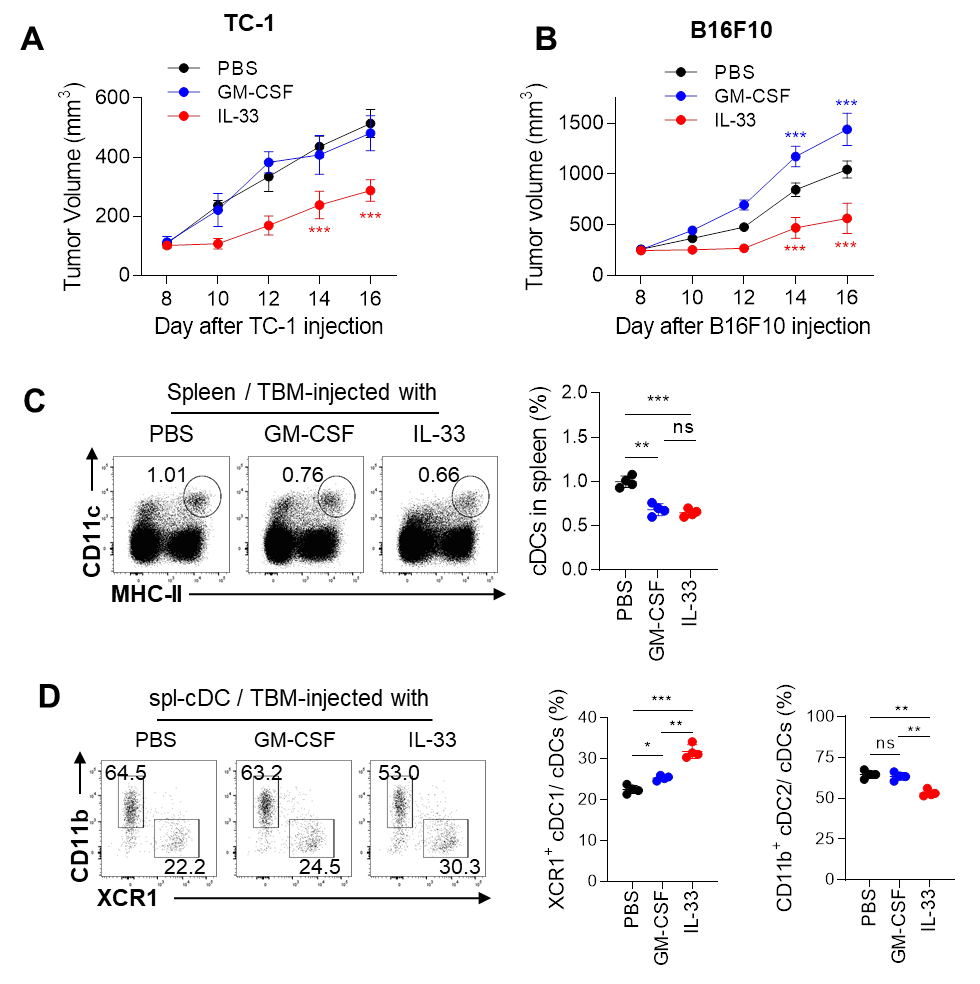


**Fig. S2. Tumor suppression and cDC and sub population changes by of exogenous GM-CSF and IL-33.**

(A-B) TC-1 (A) and B16F10 (B) tumor-bearing mice were injected i.p with 1µg of GM-CSF or IL-33 for 6 days daily after 8 days of tumor inoculation. And tumor growth was monitored, n=3 per group. (C) Total cDC population in the spleen of EG.7 TB mice i.p injected with GM-CSF and IL-33. n = 4 per group. (D) XCR1+ cDC1 and CD11b+ cDC2 populations among the splenic cDCs and splenocytes in the EG.7 TB mice i.p injected with GM-CSF and IL-33. n = 4 per group. Two-way ANOVA with Tukey for post-test (A, B) and one-way ANOVA with Dunnett T3 for post-test (C, D) were used to measure significance. *P < 0.05, **P < 0.01, ***P < 0.001; error bars indicate mean ± SD.


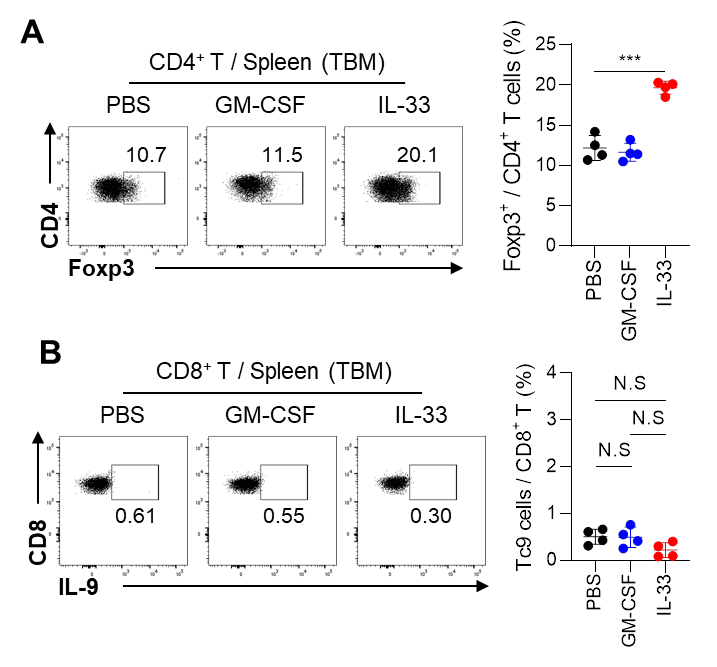
**Fig. S3. Treg and Tc9 cells in the spleens of IL-33 injected mice.** GM-CSF or IL-33 (1 µg/mouse) was i.p administrated for 6 days daily after 8 days of E.G7-tumor inoculation. (**A**) Treg population was assessed in the spleen of cytokine-injected TB mice. n=4 per group. (**B**) IL-9^+^ Tc9 population among the CD8^+^ T cells was assessed in the spleen of cytokine-injected TB mice. n=4 per group. Unpaired one-way ANOVA with Dunnett T3 for post-test were used to measure significance. ***P < 0.001; error bars indicate mean ± SD.


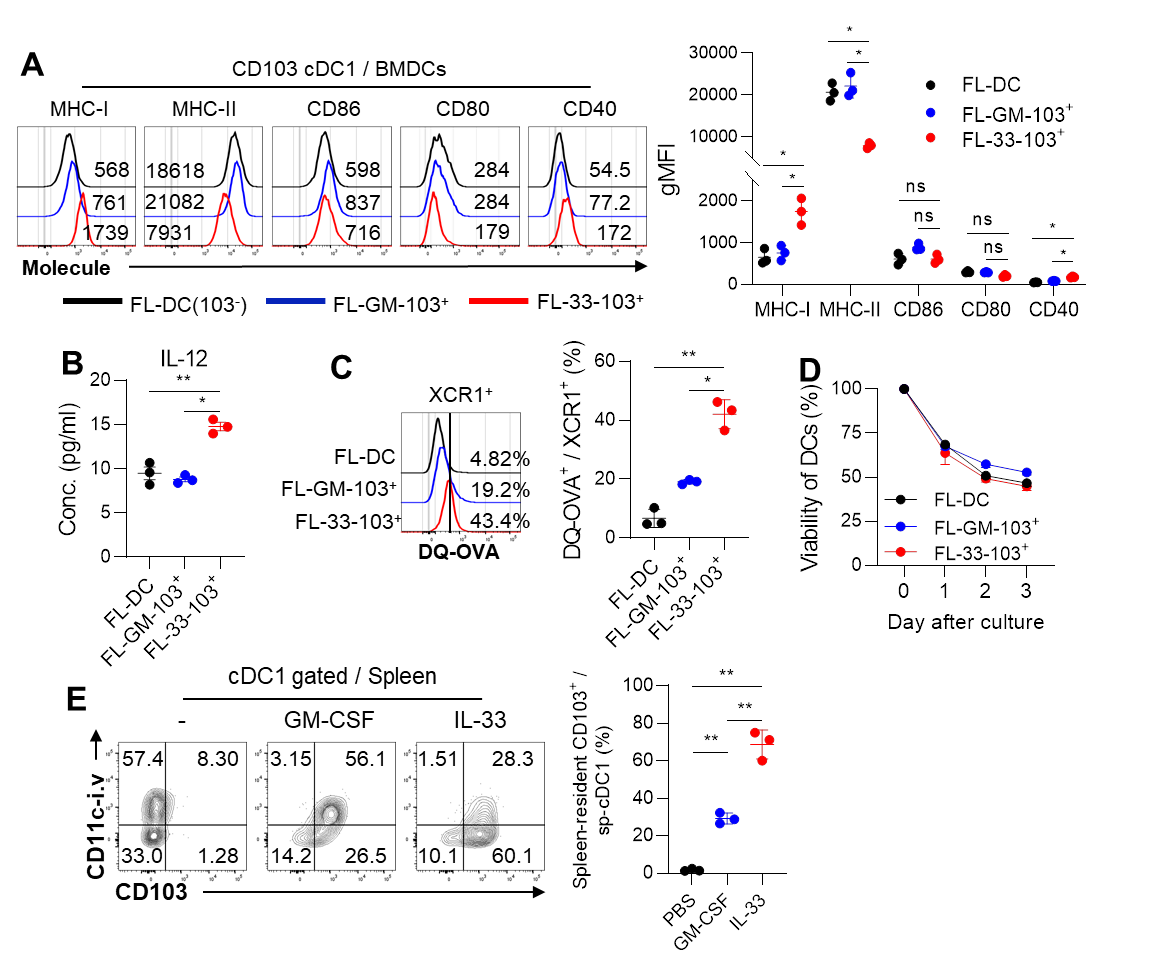
**Fig. S4. Characterization of the CD103^+^ cDC1s generated by exogenous IL-33 in comparison with GM-CSF-derived CD103^+^ cDC1s.** (**A**) MHC and co-stimulatory molecules expressed on the CD103^+^ cDC1s were assessed, and the gMFI of each molecule was demonstrated. n=3 per group. (**B**) cDC1s isolated from FL-DCs, FL-GM-DCs or FL-33-DCs were treated with LPS for 18h. Then, the level of IL-12 in the culture supernatants was analyzed by ELISA. n=3 per group. (**C**) Antigen uptake capacity of each BMDC was assessed using DQ-OVA. n=3 per group. (**D**) Each BMDC on day 9 was pulsed with OVA protein for 18h, and then co-cultured with OT-I T cells for 3 days. The viability of BMDCs was assessed daily on each co-culture. n=4 per group. (**E**) Intravascular staining of GM-CSF and IL-33 injected mice with anti-CD11c Ab, and splenic cDC1s were further assessed for their tissue residency. n=3 per group. Two-way ANOVA with Tukey (A, D) and one-way ANOVA with Dunnett T3 (B, C, E) were used to measure significance. *P < 0.05, **P < 0.01; error bars indicate mean ± SD.


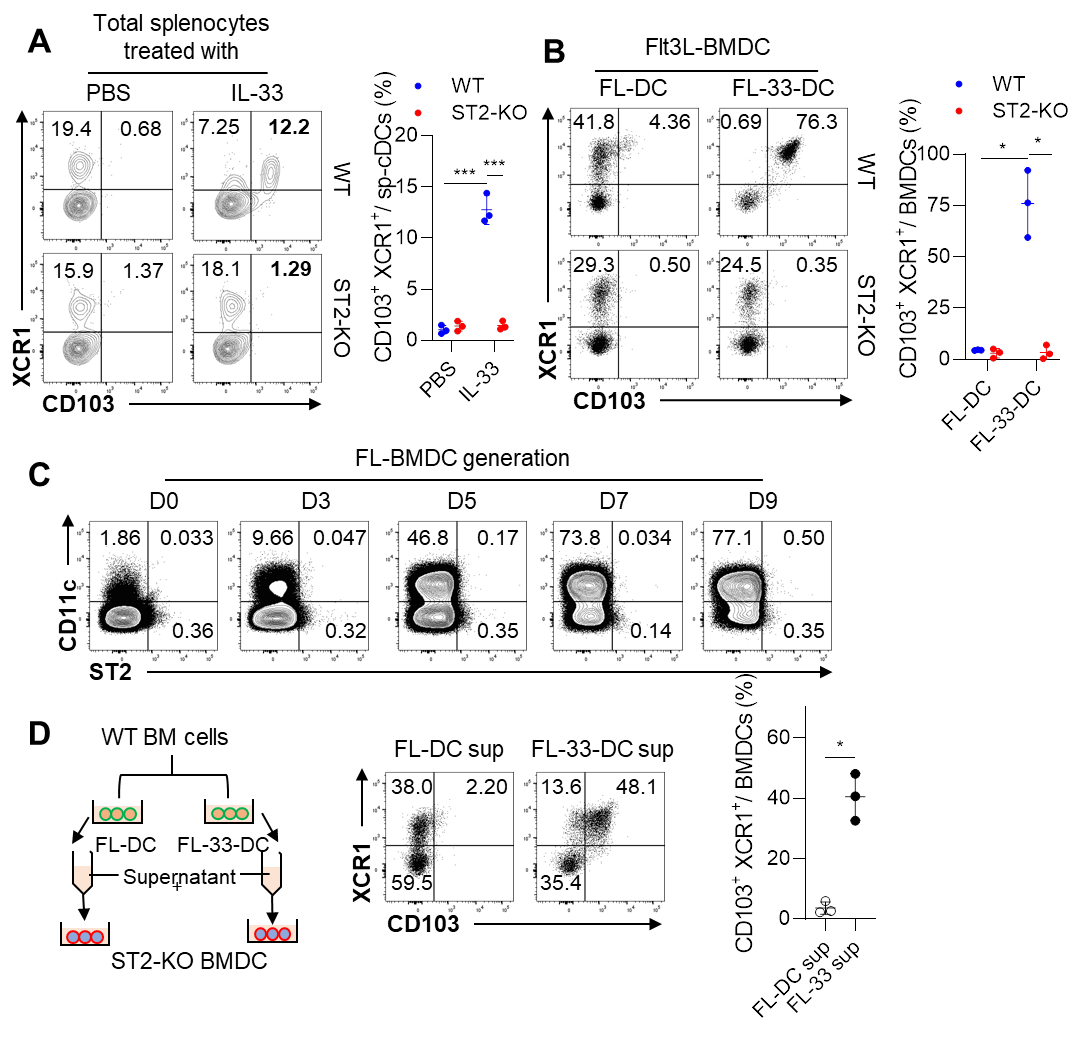
**Fig. S5. CD103^+^ cDC1s population was assessed in the WT and ST2-cKO splenocytes or BM cell cultures in the presence and absence of IL-33.** (**A**) WT (CD45.1^+^) and ST2-KO (CD45.2^+^) mouse splenocytes were cultured respectively, in presence of IL-33 for 3 days. CD103^+^ cDC1s were assessed from each sample. n=3 per group. (**B**) CD103^+^ cDC1s were assessed in the FL-33-BMDC culture of the bone marrow (BM) cells from WT and ST2-KO mice. n= 4 per group. (**C**) ST2 expression was examined during FL-BMDC generation. (**D**) The culture supernatants of FL-DCs or FL-33-DCs were harvested on day 10. Then, 50% of the culture supernatant was added to the ST2-KO Flt3L-BMDC culture on day 5. Cells were collected on day 10 and CD103^+^ cDC1s were assessed. n=3 per group. Two-way ANOVA with Tukey for post-test (A, B) and unpaired two-tailed Student’s t-test with Welch’s correction (D) were used to measure significance. *P < 0.05, ***P < 0.001; error bars indicate mean ± SD.


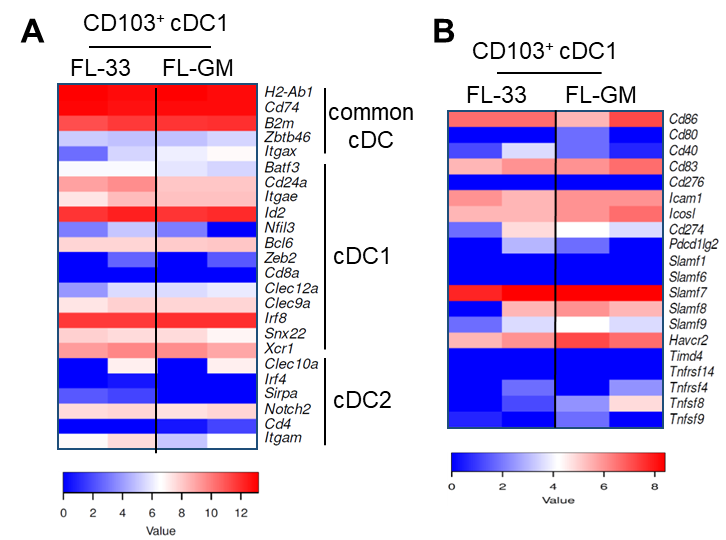


**Fig. S6. Gene expression profiles of DC-related signals in the CD103^+^ cDC1s from FL-33-DCs and FL-GM-DCs.** The heatmap shows log2 normalized value of the following genes from RNA-seq data. **(A**) Genes for MHC molecules and cDC-related genes. (**B**) Genes for co-stimulatory molecules and inhibitory molecules.

**
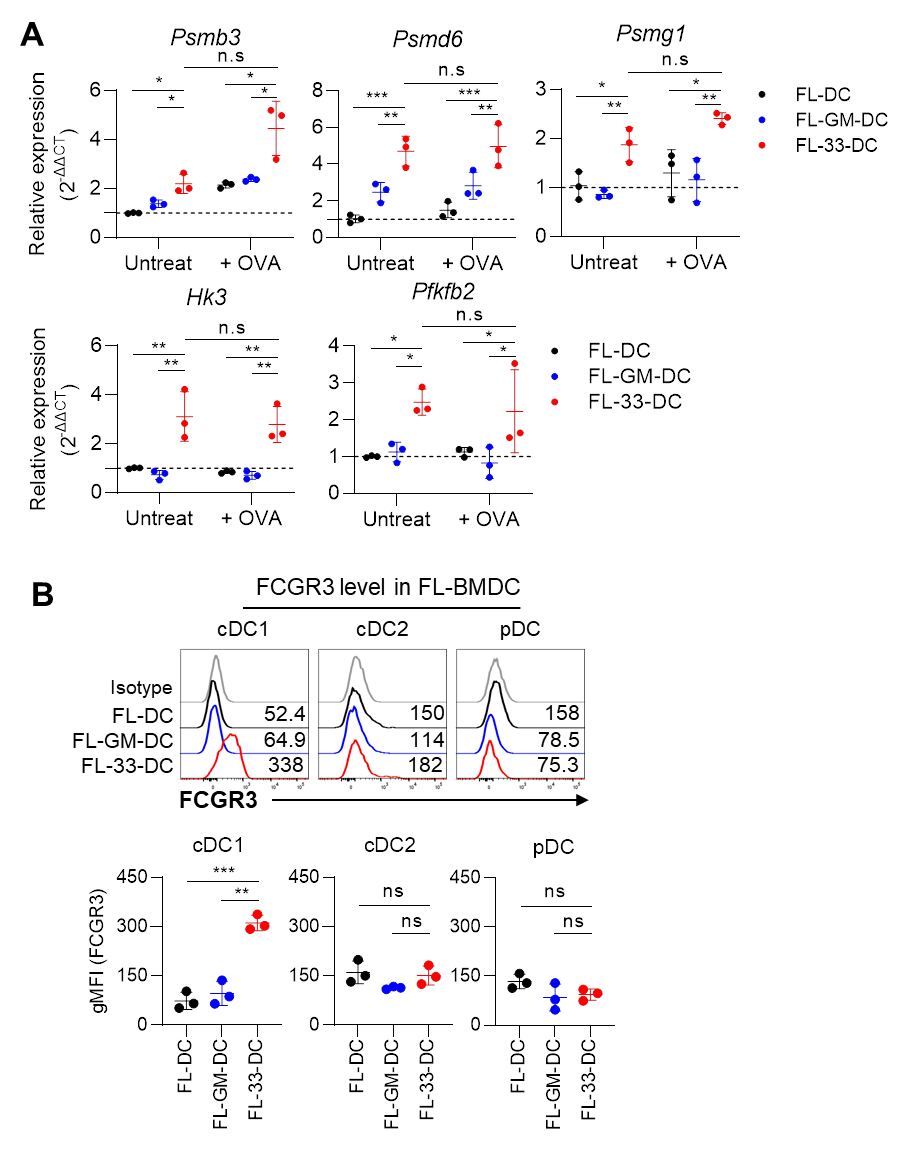
Fig. S7. (Related to Fig. 4). Expression level of several DEGs (upregulated in FL-33-DCs) in the un-pulsed or Ag-pulsed cDC1s of FL-DCs, FL-GM-DCs, and FL-33-DCs.** (A) The expression of DEGs associated with proteasome or glycolysis/gluconeogenesis was examined by qRT-PCR in the FL-DCs, FL-GM-DCs and FL-33-DCs with or without OVA-pulsing. n=3 per group. (B) FCGR3 expression was examined in the cDC1s, cDC2s, and pDCs of FL-DCs, FL-GM-DCs, and FL-33-DCs. n=3 per group. The cells were gated as cDC1: XCR1^+^ in cDCs, cDC2; XCR1^-^ CD11b^+^ in cDCs, and pDC: B220^+^ CD11c^+^. Two-way ANOVA with Tukey (A) and One-way ANOVA with Dunnett T3 (B) were used to measure significance. **P < 0.01, ***P < 0.001; error bars indicate mean ± SD.

**
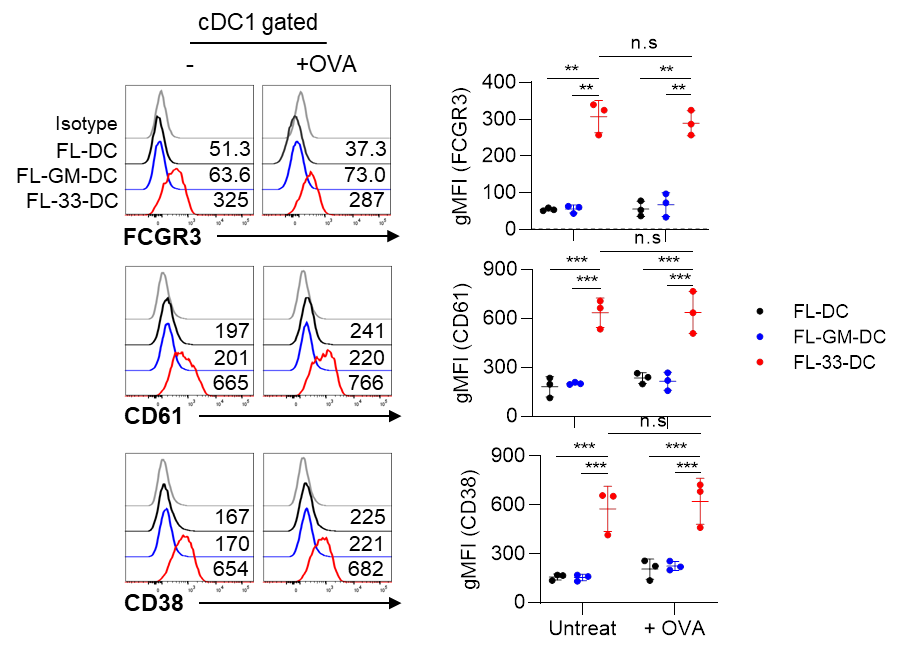
Fig. S8.** Expression of FCGR3, CD61, and CD38 was assessed in unpulsed or OVA-pulsed cDC1s of FL-DCs, FL-GM-DCs, and FL-33-DCs. The gMFIs are shown as histogram. n=3 per group. Two-way ANOVA with Tukey were used to measure significance. **P < 0.01, ***P < 0.001; error bars indicate mean ± SD.


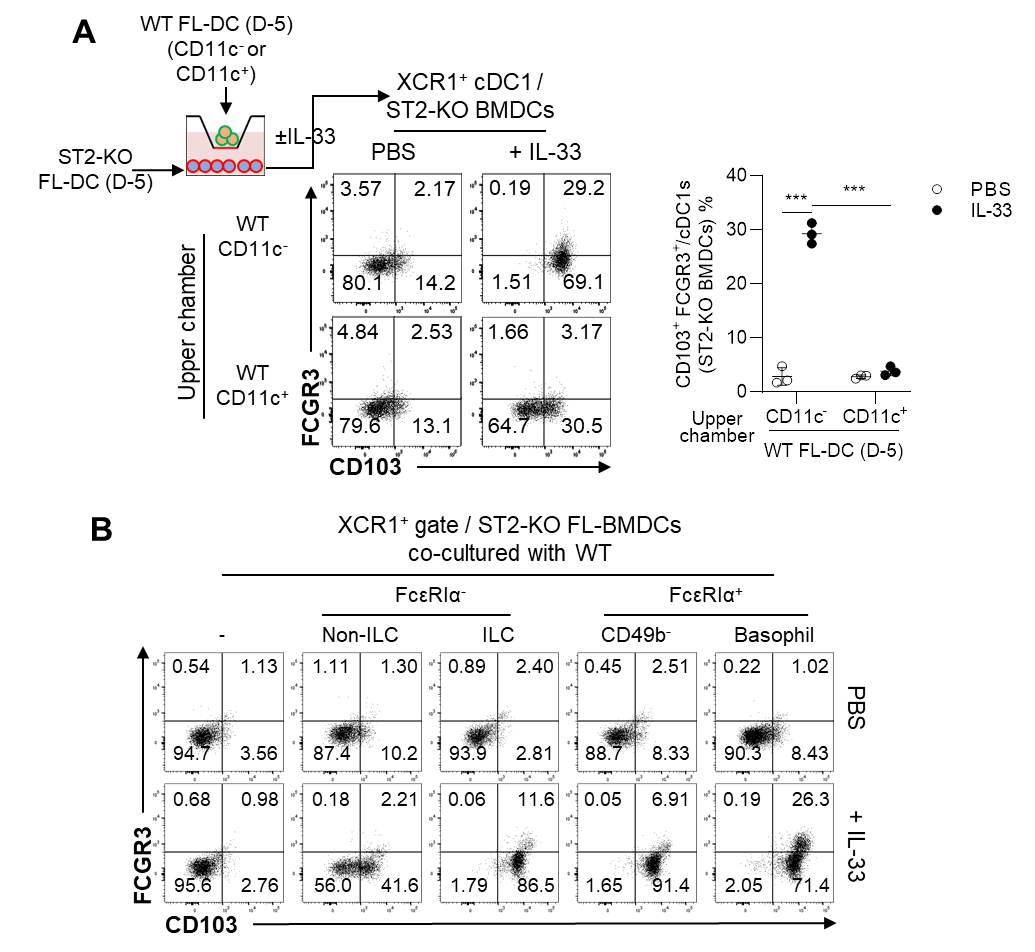
**Fig. S9. ST2^+^ basophils play a critical role in IL-33-mediated FCGR3^+^CD103^+^ cDC1s development.** (**A**) CD11c^–^ and CD11c^+^ cells isolated from WT FL-DCs on day 5 were added on the upper chamber, and ST2-KO FL-DCs on day 5 were cultured on the lower chamber of a transwell, and then cultured for additional 5 days in the presence of IL-33. FCGR3^+^CD103^+^ cDC1s were assessed in the lower chamber. n=3 per group. (**B**) WT CD11c^–^ FcεRIα^–^ cells and CD11c^–^ FcεRIα^+^ cells on day 5 of FL-DCs were isolated and co-cultured with ST2-KO FL-DCs (on day 5) for additional 5 days in the presence of IL-33. Then FCGR3^+^CD103^+^ cDC1s were assessed. Shown is a representative FACS data for the statistical data shown in Fig. 5C, Two-way ANOVA with Tukey for post-test (A) were used to measure significance. ***P < 0.001; error bars indicate mean ± SD.

**
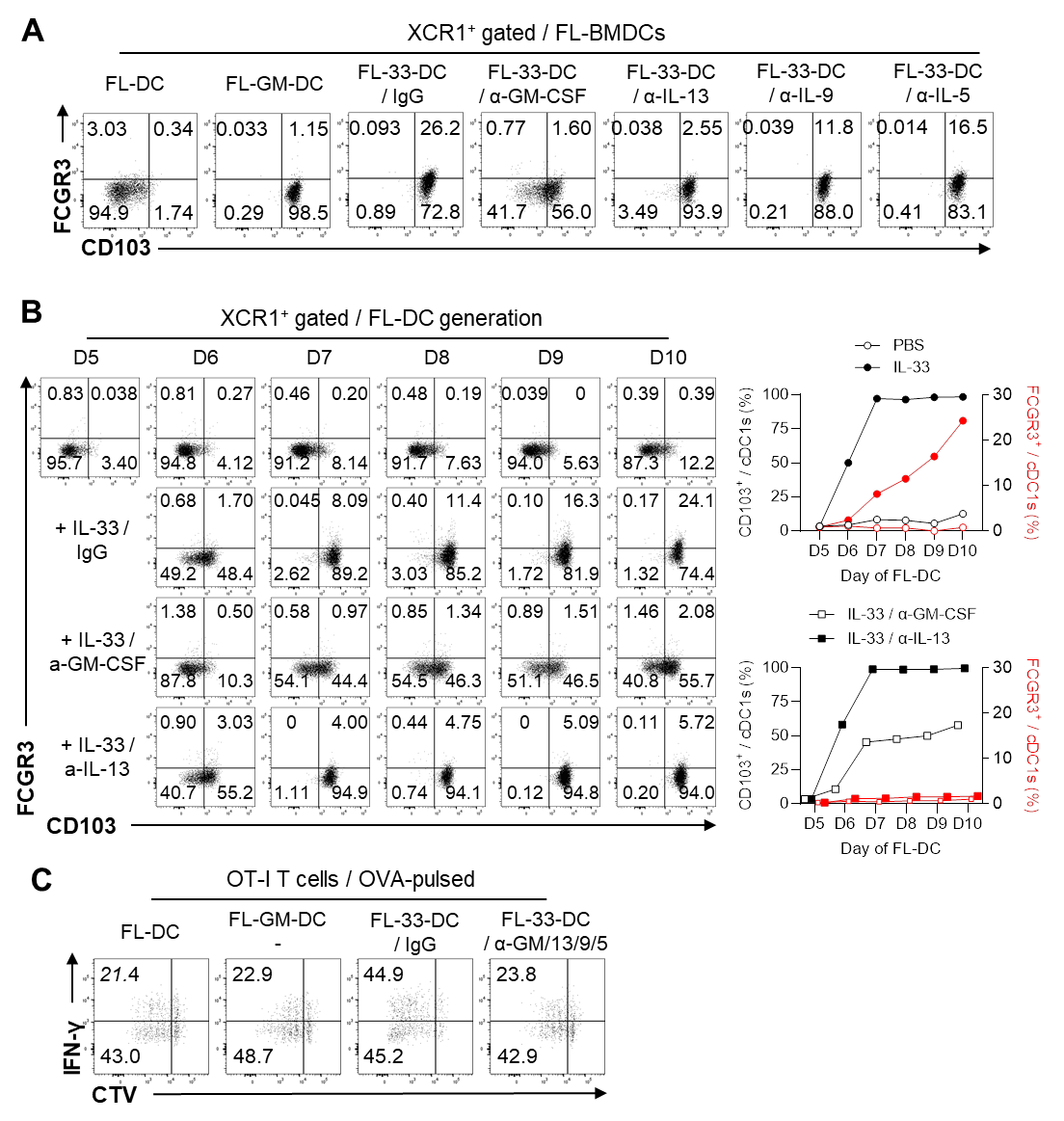
Fig. S10. IL-33-induced cytokines play an important role in IL-33-mediated FCGR3^+^CD103^+^ cDC1s development and immunogenecity.** (**A**) FL-33-DCs were generated in the presence of the neutralizing antibody against each cytokine (added on day 5), and then the frequency of FCGR3^+^CD103^+^ cDC1s were assessed in day 10. Shown is a representative FACS data for the statistical data shown in Fig. 5E. (B) FCGR3+CD103+ cDC1s were assessed in each day of FL-DC generation after IL-33 treatment with neutralizing antibody of GM-CSF and IL-13. (**C**) FL-DCs, FL-GM-DCs and FL-33-DCs generated in the presence or absence of cytokine-neutralizing antibody mixtures, were pulsed with OVA protein for 18h. cDC1s were isolated and co-cultured with CTV-labeled OT-I T cells for 4 days. Proliferating IFN-γ^+^ CD8^+^ T cell population was assessed. Shown is a representative FACS data for the statistical data shown in Fig. 5G.


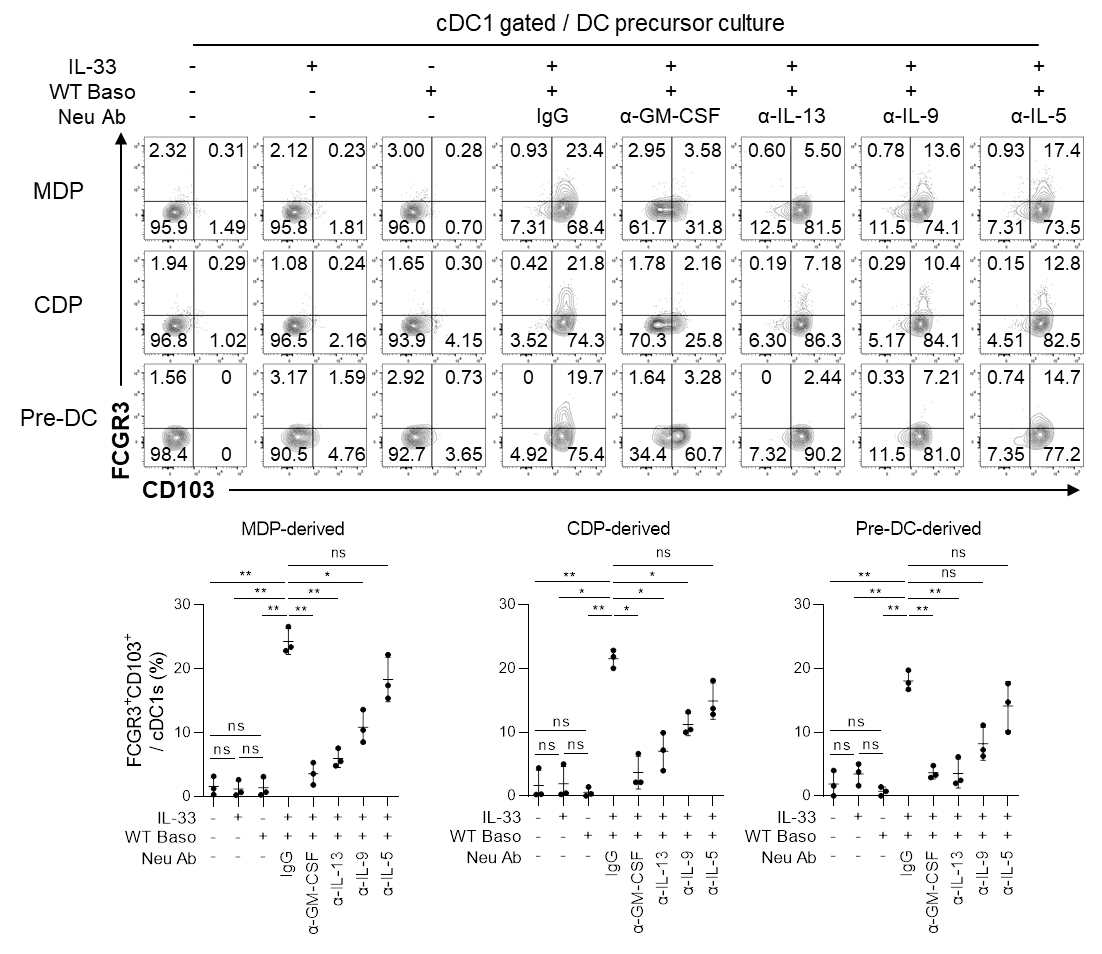


**Fig. S11. Cytokines secreted from IL-33-primed basophils play an important role in IL-33-mediated FCGR3^+^CD103^+^ cDC1s development from DC precursors (MDP, CDP, pre-DCs).** MDP, CDP, pre-DCs, and basophils isolated from BM cells of CD45.1 mice were cultured on ST2-KO (CD45.2) feeder cells, which were generated by Flt3L-cultured BM for 2 days. On day 1 of precursor and basophil culture, IL-33 and neutralizing antibodies were treated and cultured for an additional 3 days. Then, FCGR3+CD103+ cDC1s were assessed in the CD45.1-derived cells. n=3 per group. One-way ANOVA with Dunnett T3 for post-test used to measure significance. *P<0.05, **P < 0.01; error bars indicate mean ± SD.


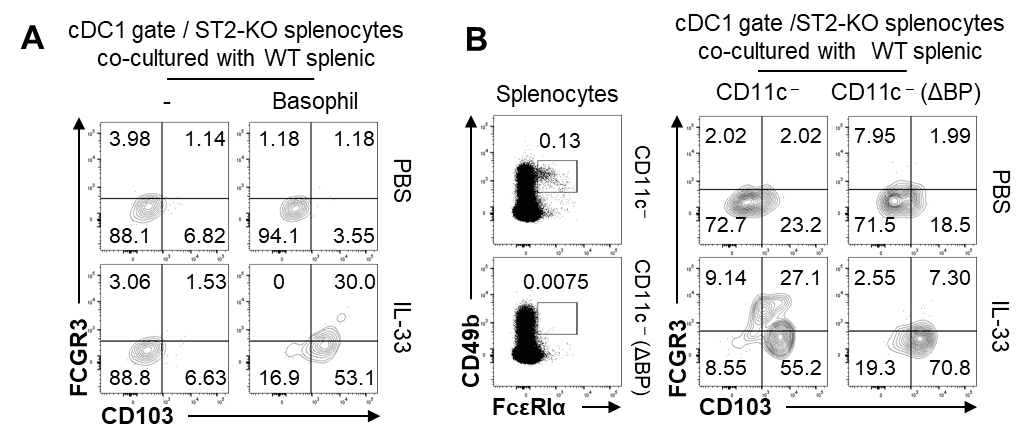


**Fig. S12. Cytokines mainly secreted from ST2^+^ basophils play an important role in IL-33-mediated FCGR3^+^CD103^+^ cDC1s development in the splenocytes.**

(**A**) ST2-KO splenocytes were co-cultured with the basophils of CD11c^–^ cells from the WT splenocytes for 2 days in the presence or absence of IL-33. FCGR3+CD103+ cDC1s were assessed by FACS, Shown is a representative FACS data for the statistical data shown in Fig. 5H. (**B**) Total (CD11c^-^) and basophil-depleted [CD11c^–^ (ΔBP), by sorting] WT CD11c ^–^ splenocytes (left). ST2-KO splenocytes were co-cultured with total or basophil-depleted CD11c- cells from WT splenocytes for 2 days in the presence or absence of IL-33. and FCGR3^+^CD103^+^ cDC1s were assessed by FACS. Shown is a representative FACS data for the statistical data shown in Fig. 5I.


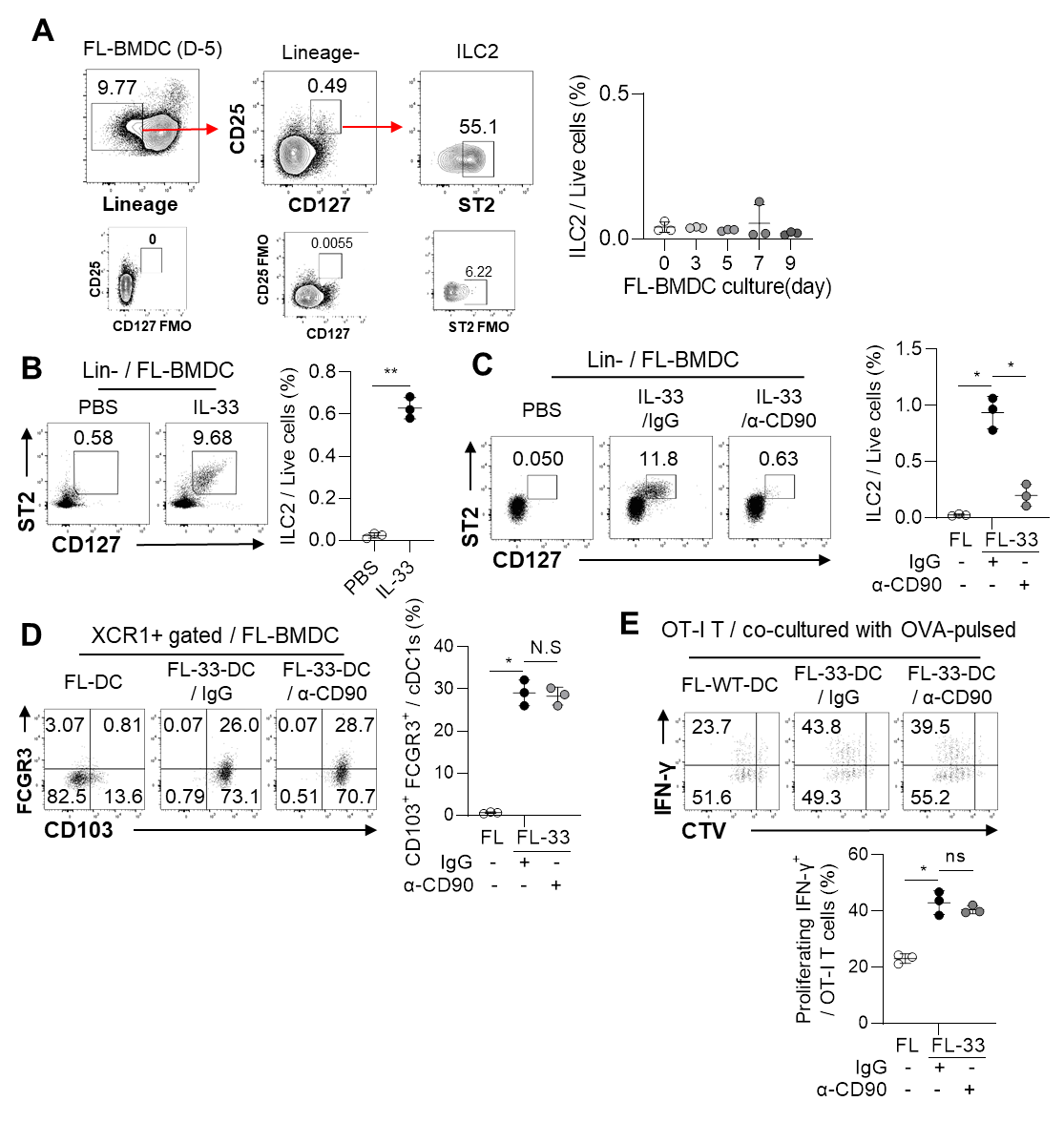


**Fig. S13. ILC2 expanded by IL-33 did little affect the differentiation and the function of CD103^+^ cDC1s from FL-33-DCs.** (A) Gating strategy for ILC2 on day 5 of FL-DCs (left). The population changes of ILC2 during the cultures of FL-BMDC (right). n=3 per group. (B) FL-BMDC cultures on day 5 were treated with IL-33 for 3 days. Then, ILC2 population as Lin^-^ CD127^+^ ST2^+^ was assessed among live cells. n=3 per group. (C) FL-BMDC cultures on day 5 were treated with IL-33 together with α-CD90 antibody to deplete ILC2 for 3 days. Then, ILC2 population as Lin^-^ CD127^+^ ST2^+^ was assessed among live cells. n=3 per group.

(D) The frequency of FCGR3^+^CD103^+^ cDC1s was assessed in the FL-33-DCs when the cultures were treated with isotype or α-CD90 antibody on day 5 for the depletion of ILC2. n=3 per group.

(E) isotype or α-CD90 antibody-treated FL-33-DCs were pulsed with OVA protein. Then, cDC1s were isolated and co-cultured with OT-I T cells for 4 days. Proliferating IFN-g^+^ CD8^+^ T cell population was assessed. n=3 per group. Unpaired two-tailed Student’s t-test with Welch’s correction (B) and one-way ANOVA with Dunnett T3 for post-test (C, D, E) used to measure significance. *P<0.05, **P < 0.01, ***P < 0.001; error bars indicate mean ± SD.

**Fig. S1**
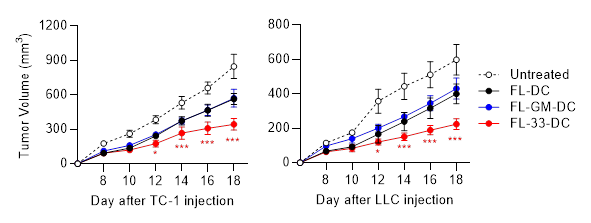
**4. Tumor immunotherapy using tumor lysate-pulsed DCs.** Tumor growth in TC-1 and LLC TB mice treated with each BMDC vaccine. Each BMDCs were pulsed with 100μg/ml of tumor cell lysate and then vaccinated into TB mice. n=4 per group. Two-way ANOVA with Tukey for post-test were used to measure significance. *P < 0.05, ***P < 0.001; error bars indicate mean ± SD.
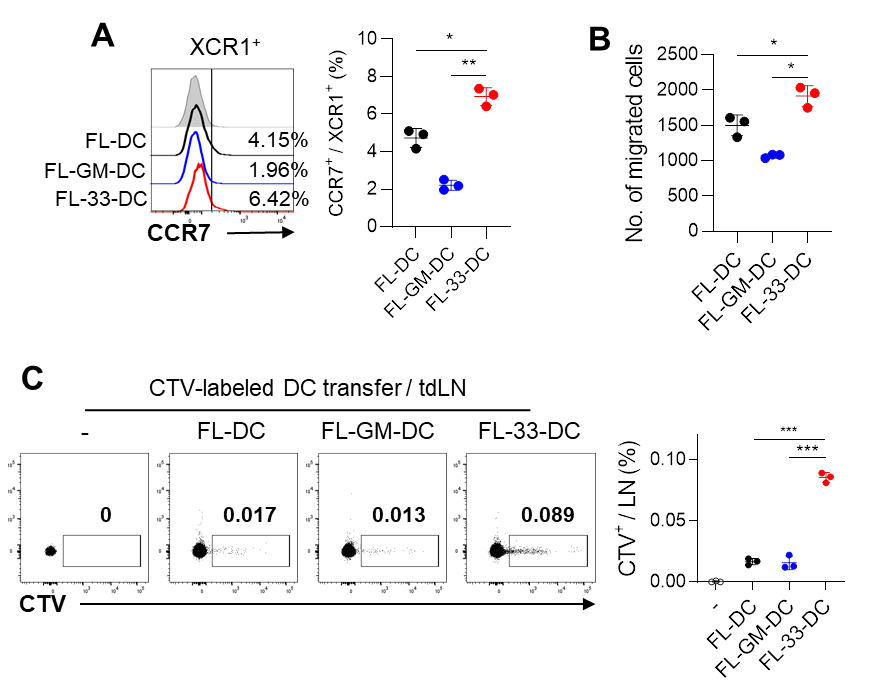
**Fig. S15. Migration capacity of FL-33-DCs in comparison with FL-GM-DCs.** (**A**) CCR7 expression level of FL-DC, FL-GM-DC, or FL-33-DCs. n=3 per group. (**B**) cDC1s (1 x 10^5^ cells) isolated from FL-DCs, FL-GM-DCs or FL-33-DCs were put on the upper chamber of transwell in the presence of CCL19 in the lower chamber and incubated for 3h. Then, the number of migrated cells was counted in the lower chamber. (**C**) cDC1s (1 x 10^6^ cells) isolated from FL-DCs, FL-GM-DCs or FL-33-DCs were labeled with CTV and then subcutaneously injected into EG.7 tumor-bearing mice. After 36h, the ratio of CTV^+^ cells in the tumor-draining lymph nodes. n=3 per group. Unpaired one-way ANOVA with Dunnett T3 was used to measure significance. *P < 0.05, **P < 0.01; error bars indicate mean ± SD.


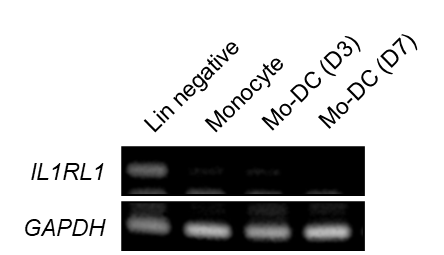


**Fig. S16. Expression of IL1RL1 (ST2) mRNA was not detected in the human monocytes and Mo-DCs during their generation.** Lin^–^ cells and monocytes were isolated from human PBMCs. Each cells were lysed by TRIzol and RNA was extracted. Then, mRNA levels of IL1RL1 was analyzed by RT-PCR

**Supplemental Methods**

**Isolation of cells from tissues**

All mice were sacrificed using CO_2_. Isolation of single cells from each tissue was performed as follows.

Bone marrow (BM): Mouse tibias and femurs were collected and flushed with phosphate-buffered saline (PBS) using a 3-ml syringe to isolate BM cells. Red blood cells were lysed with ACK lysis buffer for 5 minutes and washed twice. These cells were used for generation of Flt3L-derived BMDCs.

Spleens and lymph nodes: Spleens and lymph nodes from the mice were grained in RPMI medium, passed through a 70μm cell strainer, and centrifuged. Then, the supernatant was discarded. ACK lysing buffer was administered to lyse the red blood cells.

Tumor tissue: Dissected tumor tissues were and minced in RPMI medium at 4℃ and then physically grained. Then, the tissues were filtered through a 70-μm cell strainer. For tumor-infiltrating lymphocytes (TILs) isolation, 40%/80% Percoll density gradient centrifugation was performed at 325g for 23min. The interface layer was collected and washed twice with PBS. The cells were used for subsequent experiments.

Lungs: Lungs from DC-vaccinated B16F10-OVA TB mice were minced and dissociated in digestion buffer (86μg/ml of collagenase IV from *Clostridium histolyticum* in RPMI) for 45 minutes at 37℃. After filtration through a 70-μm cell strainer, residual red blood cells were lysed using ACK lysing buffer. The cells were washed twice and used for subsequent experiments.

**Mouse tumor model and administration of cytokines into TB mice**

E·G7 (OVA-expressing EL4; lymphoma), TC-1 (hybridoma), and B16F10 (melanoma) and LLC (lung carcinoma) cells were purchased from ATCC, and B16F10-OVA (OVA-expressing B16F10) cells were kindly provided by Dr. Lim from Sungkyunkwan University. These cell lines were cultured in cRPMI. Mice were subcutaneously inoculated with 5 x 10^5^ cells in the right flank region. The administration of cytokines for the tumor-bearing (TB) mice also followed a previously described method [[1](#_ENREF_1)]. Beginning 8 days after tumor cell inoculation, 1 μg of mIL-33 or mGM-CSF was intraperitoneally inoculated daily for 6 days. Tumor size was monitored every 2 days beginning 8 days after tumor injection and calculated as V = (short axis)^2^ x (long axis) x 1/2. 2 days after the final injection of cytokines, the spleens, draining lymph nodes, and tumor tissues were collected and suspended as single cells that were used for the subsequent experiments.

**CTL assay**

CD8^+^ T cells isolated from TILs of cytokine-administered or DC-vaccinated TB mice were co-cultured with Cell Trace Far Red (CTF)-labeled target cells (EG.7 cells) at different ratios for 4h. After Propidium Iodide staining, CTL activity was assessed by flow cytometry via the ratio of PI^+^ cells in the CTF-labeled cells.

**Adoptive transfer of OT-I T cells**

At 18h before T cell transfer, OVA protein was intravenously inoculated into control or IL-33-inoculated WT or Batf3^–/–^ mice. The CD8^+^ T cells from spleens of OT-I mice were isolated using a CD8a^+^ T Cell Isolation Kit (Miltenyi Biotec). CD8^+^ T cells were stained with Cell Trace Violet (CTV) at 37°C for 15 minutes and then washed. CTV-labeled 1 x 10^6^ CD8^+^ T cells in 200 μl were transferred intravenously into the OVA-inoculated mice. On 2.5 days after the transfer, flow cytometric analysis was performed in the splenocytes.

**Detailed flow cytometric analysis and sorting (mouse)**

Gating strategy and target molecules of each cell type was shown in the Supplemental Table 5.

All cells were suspended in FACS flow buffer (BD Bioscience) and stained in the dark with appropriate antibodies and fixable viability dye eFlour506 for 15 minutes at 4℃ as described previously [[2](#_ENREF_2)] with minor modification. Then, the cells were washed twice and used for analysis or sorting. For sorting, the suspended cells were filtered one more time in a round-bottomed polystyrene tube with a 40-μm cell strainer cap (Falcon).

For the cDC1 analysis and sorting, single cells were stained with anti-CD103-FITC or APC, anti-FCGR3-PE, anti-XCR1-PE or APC, B220-PerCPcy5.5 (for FL-DCs), anti-CD11c-PEcy7, anti-MHC-II-APCcy7, and anti-CD11b-Pacific blue. Anti-CD86-FITC, anti-CD80-PE, anti-FCGR3-PE anti-CD40-PerCPcy5.5, and anti-MHC-I-Pacific Blue were additionally stained for investigation of the surface molecules in the cDC1s from FL-DCs, FL-GM-DCs, and FL-33-DCs.

For the surface staining of CD8^+^ T cell, single cells were stained with anti-CD62L-FITC, anti-CD8-PEcy7, anti-CD44-APC, and anti-CD3-APCcy7. Single cells were already stimulated with cell activation cocktail (Biolegend) for 4h before intracellular staining of CD8^+^ T cells, and then anti-IL-9-PE, anti-CD8-PEcy7, anti-IFN-γ-APC, and anti-CD3-APCcy7 were stained according to protocol of Fixation/Permeablization Kit (BD biosciences).

For the intracellular staining of CD4^+^ T cell, single cells were stained as anti-CD4-PEcy7, anti-Foxp3-APC, and anti-CD3-APCcy7 according to protocol of Foxp3 / Transcription Factor Staining Buffer Set (eBiosciences).

The single cells in the BM were stained with anti-CD3/B220/Ly6G/CD11b (Lin)-FITC, anti-CD135-PE, anti-CD11c-PerCPcy5.5, anti-CD115-PEcy7, anti-ST2-APC, anti-MHC-II-APCcy7, and anti-CD117-Pacific blue for ST2 expression analysis in the DC precursors (MDP, CDP, pre-DC).

The single cells in the naïve BM cells or some days of FL-DCs were stained with anti-Lin-FITC, anti-CD127-PE, anti-CD25-PerCPcy5.5, and anti-ST2-APC for the population of type 2 innate lymphoid cells (ILC2s). ST2 expression was analyzed in the Lin^–^CD127^+^ total ILCs of the BM cells and ILC2 population was analyzed as Lin^–^CD127^+^CD25^+^ST2^+^.

For basophil/ILC analysis and sorting in the CD11c^–^ cells, single cells from total splenocytes or day 5 of FL-DCs were stained with anti-FcεRIα-FITC, anti-CD49b-PE, anti-CD3/B220/CD11b-PerCPcy5.5, anti-CD127-PEcy7, and anti-CD11c-eF450. In the splenocytes, basophils were isolated or depleted. On day 5 FL-DCs, total CD11c^–^ cells were isolated, or the cells were subdivided as following gate; ILCs, non-ILCs, CD49b^–^ portion, and basophils. The cells were analyzed with following gate and sorted cells were used for subsequent experiments.

**Spectrum flow cytometry**

Flow cytometry with Aurora (Cytek, USA) was used for analyzing single cells with 8 markers. Single cells were stained with zombie NIR fixable viability dye for 15 minutes at at 4℃. The cells were then stained with anti-CD24-Pacific Blue, anti-CD103-BV711, anti-MHC-II-BV785, anti-CD8a-BB515, anti-CD11c-AF532, anti-PE-XCR1, and anti-CLEC9A-APC in FACS buffer with Super Bright Complete Staining Buffer (Thermo Fisher Scientific) for 15 minutes at 4℃. After washing twice with FACS buffer, cells were detected via Aurora flow cytometry. Data was analyzed with t-distributed stochastic neighbor embedding (t-SNE) with FlowJo software version 10.

**cDC1 / OT-I T cell co-culture**

cDC1s and OT-I T cells were co-cultured as described previously with minor modification [[3](#_ENREF_3), [4](#_ENREF_4)]. In brief, CD8^+^ T cells were purified from splenocytes of OT-I mice using a mouse CD8^+^ T cell isolation kit II (Miltenyi Biotec) and labeled with CTV for 15 minutes at 37℃. *In vivo* cDC1s from spleens were pulsed with OVA protein for 18h. Then the DCs were co-cultured with 2.5 x 10^5^ CD8^+^ T cells at the ratio of 1:20 (DC: T) for 4 days. *In vitro*–generated FL-DCs were pulsed with OVA for 18h before cell collection. cDC1s were isolated, and then 2.5 x 10^5^ CD8^+^ T cells were co-cultured at the ratio of 1:5 (DC: T) for 4 days. DC viability in the CTV^–^ cells or cell proliferation and cytokine expression of CD8^+^ T cells were assessed by flow cytometry.

**RNA isolation**

The cells were lysed with 500μl of Trizol (Life Technologies). RNA purification was performed using a PicoPure RNA Isolation Kit (Applied Biosystems) per the manufacturer’s protocols. RNA quality was assessed by Agilent 2100 bioanalyzer using the RNA 6000 Nano Chip (Agilent Technologies, Am stelveen, The Netherlands), and RNA quantification was performed using ND 2000 Spectrophotometer (Thermo Inc., DE, USA) in ebiogen Inc (Seoul, Korea).

**RNA sequencing and analysis**

The library of RNA was constructed using a QuantSeq 3’ mRNA Seq Library Prep Kit (Lexogen, Inc. Inc., Austria) according to the manufacturer’s instructions. The library was amplified to add the complete adapter sequences required for cluster generation and purified. Then, high-throughput single-end 75-bp sequencing was performed on a NextSeq 500 (Illumina, Inc., USA). Alignment of the QuantSeq 3’ mRNA-Seq reads was performed using Bowtie2 [[5](#_ENREF_5)]. The alignment file was used to assemble transcripts, estimate their abundance, and detect the differential expression of genes. Differentially expressed genes were determined based on counts from unique and multiple alignments using coverage in Bedtools [[6](#_ENREF_6)]. The read count data were processed based on the quantile normalization method using EdgeR [[7](#_ENREF_7)]. These sequencing procedures from purified RNA were executed in ebiogen Inc (Seoul, Korea). The gene ontology analysis of genes up-regulated in the FL-33-DCs was based on searches done in DAVID (http://david.abcc.ncifcrf.gov/). For the GSEA (Gene Set Enrichment Analysis), GSEA v4.0.3 software was applied. Gene sets for analysis were obtained from the Gene Set Knowledgebase (http://ge-lab.org/gs/), a curated functional genomics database for murine transcriptomics. An enrichment plot was used to visualize the GSEA results. The enrichment score and false discovery rate values were applied after gene set permutations were performed 1000 times for the analysis.

**Antigen uptake/processing assay**

XCR1^+^ cDC1s (2 x 10^5^ cells) were isolated from FL-DCs, FL-GM-DCs, and FL-33-DCs. 1 μg/ml of DQ-OVA containing RPMI was administered and incubated for 3h at 37℃. Then, cold RPMI was added, the cells were washed twice with PBS, and DQ-OVA expression was analyzed.

**Transwell culture**

On day 5 of FL-DC generation, ST2-KO cells were plated in the lower part of 24-well transwell chambers. WT cells were plated in the upper chamber, and 5 ng/ml mIL-33 was administered for an additional 5 days. Then, the cells from the lower chamber were analyzed.

**Measurement of cytokines**

XCR1^+^ cDC1s (1 x 10^6^ cells) cells from FL-DCs, FL-GM-DCs, and FL-33-DCs were cultured on 24-well plates with 100μg/ml of LPS for 18h. Then, the culture supernatants were collected. Cytokine levels were measured using ELISA MAX™ Deluxe Set Mouse IL-12 (p70) (Biolegend).

CD127^–^ non-ILCs or CD127^+^ ILCs from FcεRIα^–^ cells and CD49b^+^ basophils or CD49b^–^ portions from FcεRIα^+^ cells taken at 2 x 10^4^ cells on day 5 of FL-DCs were cultured on 96-well plates with or without IL-33 for 36h. Then, the culture supernatants were collected. Cytokine levels were measured using mouse GM-CSF, IL-5, and IL-9 ELISA MAX Deluxe (Biolegend) and mouse IL-13 Qunatikine ELISA (R&D Systems).

**DC precursor culture with basophils, IL-33, and neutralizing antibodies**

MDPs, CDPs, and pre-DCs as DC precursors were cultured as described previously with minor modification [[8](#_ENREF_8)]. Flt3L-stimulated BM cultures were generated from ST2-KO mice (CD45.2^+^) by seeding of 2 x 10^6^ cells into 12-well plates in cRPMI supplemented with 100ng/ml recombinant human Flt3L, 10 mM HEPES, and 55 μM β-mercaptoethanol. After 2 days, 5 × 10^3^ sorted CD45.1^+^ DC precursors were added to indivisual wells with or without WT basophils. After a day of addition of DC precursors, IL-33 and neutralizing antibodies were treated and incubated for an additional 3 days. Then, cDC1s derived from CD45.1^+^ cells were assessed by flow cytometry.

**DC-based tumor immunotherapy (detailed)**

EG.7 cells (5 x 10^5^ cells) were subcutaneously inoculated into mice. FL-DCs, FL-GM-DCs, and FL-33-DCs on day 9 were pulsed with OVA protein for 18h. Then, live XCR1^+^ cDC1s from OVA-pulsed FL-DCs, FL-GM-DCs, and FL-33-DCs (1 x 10^6^ cells) were sorted and subcutaneously injected into mice on days 3 and 10 after tumor cell injection. Tumor size was measured every 2 days beginning 8 days after tumor cell injection. The immune cell analysis was performed on day 18. For lung metastatic models, B16F10-OVA cells (1.5 x 10^5^ cells) were intravenously injected into mice. Live XCR1^+^ cDC1s (1 x 10^6^ cells) from OVA-pulsed FL-DCs, FL-GM-DCs, and FL-33-DCs were sorted and intravenously injected days 3 and 10 after tumor cell inoculation. On day 18 of tumor cell injection, the lungs were collected for nodule count and immune cell analysis.

***In vitro* Chemotaxis**

XCR1^+^ cDC1s (1 x 10^5^ cells) were isolated from FL-DCs, FL-GM-DCs, and FL-33-DCs and resuspended in 100μl of RPMI. Then, the cells were put in the upper chamber of a transwell (Corning Costar). 300 ng/ml recombinant murine CCL19 (Biolegend) in 500 μl of RPMI was added to the lower chamber of the transwell. Then, the transwells were incubated for 3h at 37°C. The cells were collected from the lower chamber and counted.

***In vivo* DC migration**

XCR1^+^ cDC1s (1 x 10^6^ cells) were isolated from FL-DCs, FL-GM-DCs, and FL-33-DCs and labeled with CTV. Then, the cells were subcutaneously injected into EG.7 tumor-bearing mice on day 10. After 36h, draining LNs were collected and the ratio of CTV^+^ cells were analyzed.

**Isolation of peripheral blood mononuclear cells (PBMCs) from human blood**

Blood from healthy volunteers was collected in heparin-coated vacutainers (BD Bioscience). PBMCs were isolated using Ficoll-Paque (1.077g/ml, GE Healthcare). In brief, blood was put on the Ficoll, and then centrifuged at 2000 rpm and 20 min. The interface layer was collected and residual red blood cells were lysed using ACK lysing buffer. The cells were washed twice and used for subsequent experiments.

**Measurement of human IL-12**

Poly I:C (30 μg/ml) was administered on day 6 and incubated for 24h, and then the culture supernatants were collected. The IL-12 levels were measured using a LEGEND MAX™ Human IL-12 (p70) ELISA Kit (Biolegend).

**Detailed flow cytometric analysis and sorting (human)**

Cells were analyzed on a FACSCanto II and sorted on a FACSAria Fusion. Single cells from human PBMCs were stained with anti-CD3/CD16/CD19/CD56/CD66b-PerCPcy5.5, anti-CD14-PEcy7, and anti-CD8-APCcy7. The cells were isolated as Supplemental Table 6. CD8^+^ T cells were stained with CTV and the CTV-labeled CD8^+^ T cells were used for allogenic CD8^+^ T/Mo-DC co-culture.

Anti-HLA-A,B,C(HLA-I)-FITC, Anti-HLA-DP,DQ,DR(HLA-II)-FITC, anti-CD40-FITC, anti-CD83-FITC, anti-CD80-PE, anti-CD86-PE, and anti-CD11c-APC were used for the surface phenotypes of the hMo-DCs.

**Supplemental Tables**

**Supplemental Table 1. Dyes and antibodies for flow cytometry (mouse samples)**

* AF = Alexa flour, eF = eFlour^TM^, BB = Brilliant^TM^ Blue, BV = Brilliant Violet^TM^

| Antibody | Clone | Source | Catalog# | Diluted ratio |
| --- | --- | --- | --- | --- |
| anti-MHC-I-Pacific blue | AF6-88.5 | Biolegend | 116514 | 1:200 |
| anti-MHC-II-APCcy7 | M5/114.15.2 | Biolegend | 107628 | 1:200 |
| anti-MHC-II-BV785 | M5/114.15.2 | Biolegend | 107645 | 1:200 |
| anti-CD11c-PerCPcy5.5 | N418 | Biolegend | 117326 | 1:100 |
| anti-CD11c-PEcy7 | N418 | eBiosciences | 25-0114 | 1:200 |
| anti-CD11c-eF450 | N418 | eBiosciences | 48-0114 | 1:200 |
| anti-CD11c-AF532 | N418 | eBiosciences | 58-0114 | 1:100 |
| anti-XCR1-PE | ZET | Biolegend | 148204 | 1:200 |
| anti-XCR1-APC | ZET | Biolegend | 148206 | 1:200 |
| anti-CD8a-BB515 | 53-6,7 | BD Biosciences | 564422 | 1:200 |
| anti-CD8a-PEcy7 | 53-6,7 | eBiosciences | 25-0081 | 1:200 |
| anti-CD103-FITC | 2E7 | Biolegend | 121420 | 1:200 |
| anti-CD103-APC | 2E7 | eBiosciences | 17-1031 | 1:200 |
| anti-CD103-BV711 | 2E7 | Biolegend | 121435 | 1:200 |
| anti-CLEC9A-APC | 7H11 | Biolegend | 143506 | 1:100 |
| anti-CD24-Pacific Blue | M1/69 | Biolegend | 101820 | 1:200 |
| anti-CD45.1-Pacific Blue | A20 | Biolegend | 110722 | 1:200 |
| anti-CD45.2-PerCPcy5.5 | 104 | eBioscience | 45-0454 | 1:100 |
| anti-FcγRIII(FCGR3)-PE | # 275003 | R&D Systems | FAB19601P | 1:20 |
| anti-CD61 FITC | 2C9.G2(HMβ3-1) | Biolegend | 104306 | 1:400 |
| anti-CD38 FITC | 90 | Biolegend | 102705 | 1:400 |
| Zombie NIR fixable viability dye | - | Biolegend | 423106 | 1:1000 |
| Fixable Viability Dye- eFluor™ 506 | - | eBiosciences | 65-0866 | 1:400 |
| anti-CD44-FITC | IM7 | Biolegend | 103006 | 1:200 |
| anti-CD62L-eF450 | MEL-14 | eBiosciences | 48-0621 | 1:200 |
| anti-CD4-PEcy7 | GK1.5 | eBiosciences | 25-0041 | 1:200 |
| anti-FcεRIα-FITC | MAR-1 | Biolegend | 134305 | 1:200 |
| anti-CD49b-PE | DX5 | Biolegend | 108908 | 1:200 |
| anti-CD127-PE | eBioSB/199 (SB/199) | eBiosciences | 12-1273 | 1:100 |
| anti-CD127-PEcy7 | eBioSB/199 (SB/199) | eBiosciences | 25-1273 | 1:100 |
| anti-CD25-PerCPcy5.5 | PC61.5 | eBiosciences | 45-0251 | 1:200 |
| anti-ST2-APC | RMST2-2 | eBiosciences | 17-9335 | 1:100 |
| anti-CD135-PE | A2F10 | Biolegend | 135306 | 1:100 |
| anti-CD115-PEcy7 | AFS98 | eBiosciences | 25-1152 | 1:200 |
| anti-CD117-eF450 | 2B8 | eBiosciences | 48-1171 | 1:200 |
| anti-CD3-FITC | 17A2 | Biolegend | 100204 | 1:200 |
| anti-CD3-APCcy7 | 17A2 | Biolegend | 100222 | 1:200 |
| anti-B220-FITC | RA3-6B2 | Biolegend | 103206 | 1:200 |
| anti-B220-PerCPcy5.5 | RA3-6B2 | Biolegend | 103234 | 1:200 |
| anti-Ly6G-FITC | 1A8 | Biolegend | 127606 | 1:200 |
| anti-CD11b-FITC | M1/70 | Biolegend | 101206 | 1:200 |
| anti-CD11b-PerCPcy5.5 | M1/70 | Biolegend | 101228 | 1:200 |
| anti-CD11b-Pacific Blue | M1/70 | Biolegend | 101224 | 1:200 |
| anti-CD86-FITC | GL-1 | Biolegend | 105006 | 1:200 |
| anti-CD80-PE | 16-10A1 | BD Biosciences | 553769 | 1:200 |
| anti-CD40-PerCPcy5.5 | 3/23 | Biolegend | 124624 | 1:100 |
| anti-H-2K^b^-Pacific blue | AF6-88.5 | Biolegend | 116514 | 1:200 |
| anti-Foxp3-APC | FJK-16s | eBiosciences | 17-5773 | 1:100 |
| anti-IL-9-PE | RM9A4 | Biolegend | 514104 | 1:100 |
| anti-IFN-γ-APC | XMG1.2 | Biolegend | 505810 | 1:100 |
| Propidium Iodide | - | BD Biosciences | 556463 | 1:50 |
| Cell Trace Violet | - | Invitrogen | C34557 | 1:1000 (5μM) |
| Cell Trace Far Red | - | Invitrogen | C34564 | 1:1000 (5μM) |

**Supplemental Table 2. Dyes and antibodies for flow cytometry (human samples)**

| Antibody | Clone | Source | Catalog# | Diluted ratio |
| --- | --- | --- | --- | --- |
| anti-HLA-A,B,C-FITC | G46-2.6 | BD Biosciences | 555552 | 1:20 |
| anti-HLA-DR,DP,DQ-FITC | Tu39 | BD Biosciences | 555558 | 1:20 |
| anti-CD11c-APC | B-ly6 | BD Biosciences | 559877 | 1:20 |
| anti-CD86-PE | 2331  (FUN-1) | BD Biosciences | 555658 | 1:20 |
| anti-CD80-PE | 2D10.4 | BD Biosciences | 566992 | 1:20 |
| anti-CD40-FITC | 5C3 | BD Biosciences | 555588 | 1:20 |
| anti-CD83-FITC | HB15e | BD Biosciences | 556910 | 1:20 |
| anti-CD3-PerCPcy5.5 | SP34-2 | BD Biosciences | 552852 | 1:20 |
| anti-CD16-PerCPcy5.5 | 3G8 | Biolegend | 302028 | 1:50 |
| anti-CD19-PerCPcy5.5 | HIB19 | Biolegend | 302230 | 1:50 |
| anti-CD56-PerCPcy5.5 | MEM-188 | Biolegend | 304626 | 1:50 |
| anti-CD66b-PerCPcy5.5 | G10F5 | Biolegend | 305108 | 1:50 |
| anti-CD14-PEcy7 | 61D3 | eBiosciences | 25-0147 | 1:50 |
| anti-CD8-APCcy7 | RPA-T8 | Biolegend | 301016 | 1:50 |
| anti-IFN-γ-PEcy7 | 4S.B3 | Biolegend | 502528 | 1:50 |
| Fixable Viability Dye- eFluor™ 506 | - | eBiosciences | 65-0866 | 1:400 |
| Cell Trace Violet | - | Invitrogen | C34557 | 1:1000 (5μM) |

**Supplemental Table 3. Chemicals, cell lines, antibodies and recombinant proteins used in this study**

| Reagent | Source | Catalog Number |
| --- | --- | --- |
| Recombinant mouse IL-33 | Biolegend | 580506/580508 |
| Recombinant human IL-33 | In housing | - |
| Recombinant human Flt3L | Creagene | JW-H004 |
| Recombinant mouse GM-CSF | Creagene | JW-M001 |
| Recombinant human GM-CSF | Creagene | JW-H010 |
| Recombinant human IL-4 | Creagene | JW-H002 |
| Recombinant mouse CCL19 | Biolegend | 587802 |
| DQ™ Ovalbumin | Invitrogen | D12053 |
| Ovalbumin | Sigma-Aldrich | A5503 |
| EG.7 | ATCC | CRL-2113 |
| B16F10 | ATCC | CRL-6475 |
| B16F10-OVA | Provided by Yong-Taik Lim | - |
| 4T1 | ATCC | CRL-2493 |
| LLC | ATCC | CRL-1642 |
| Collagenase IV from *Clostridium histolyticum* | Sigma-Aldrich | C5138 |
| RPMI 1640 Medium | Gibco | 11875119 |
| Fetal Bovine Serum (FBS) | Gibco | 12483020 |
| Penicillin/Streptomycin | Gibco | 15140122 |
| Hydroxyethyl piperazine Ethane Sulfonic acidd (HEPES) | Gibco | 15630080 |
| β-mercaptoethanol | Sigma-Aldrich | M3148 |
| ACK lysing buffer | Gibco | A1049201 |
| Percoll | Sigma-Aldrich | P1644 |
| TRIzol™ Reagent | Invitrogen | 15596018 |
| PicoPure™ RNA Isolation Kit | Applied Biosystems | KIT0204 |
| anti-mouse GM-CSF (Clone: MP1-22E9) | BioXcell | BE0259 |
| anti-mouse IL-13 (Clone: eBio1316H) | Invitrogen | 16-7135 |
| anti-mouse IL-9 (Clone: 9C1) | BioXcell | BE0181 |
| anti-mouse IL-5 (Clone: TRFK5) | BioXcell | BE0198 |
| anti-mouse CD90.2 (Clone: 30H12) | BioXcell | BE0066 |

**Supplemental Table 4. Primers for (q)RT-PCR (mouse and human)**

| Mouse *Psmb3* Forward | 5’-GCGGTTCGGTCCCTACTACA-3’ |
| --- | --- |
| Mouse *Psmb3* Reverse | 5’-ATGGGACAGCCAATGAGGTC-3’ |
| Mouse *Psmd6* Forward | 5’-CCTGATAGCAAGAACTGGCA-3’ |
| Mouse *Psmd6* Reverse | 5’-GGAACACGTTGAAAAGTGGC-3’ |
| Mouse *Psmg1* Forward | 5’-GGATCAGCAGTTCCAGTGGT-3’ |
| Mouse *Psmg1* Reverse | 5’-AGGAAAGGCTGCAGGTAGAC-3’ |
| Mouse *Hk3* Forward | 5’-GAGACCCTGGCACCATTTCA-3’ |
| Mouse *Hk3* Reverse | 5’-TCTCCTTGAAGCCCCCTGAT-3’ |
| Mouse *Pfkfb2* Forward | 5’-ACAAGGTCACTTACCAGCCC-3’ |
| Mouse *Pfkfb2* Reverse | 5’-GACCAGGAATCTCTGGCCTA-3’ |
| Mouse *Gapdh* Forward | 5’-TGATGGGTGTGAACCACGAG-3’ |
| Mouse *Gapdh* Reverse | 5’-AGTGATGGCATGGACTGTGG-3’ |
| Human *IL1RL1* Forward | 5’-TCATCCAGAACGACGCCAAG-3’ |
| Human *IL1RL1* Reverse | 5’-ATGCTGGAGGGAGTCCTGAA-3’ |
| Human *GAPDH* Forward | 5’-GTCAAGGCTGAGAACGGGAA-3’ |
| Human *GAPDH* Reverse | 5’-AAATGAGCCCCAGCCTTCTC-3’ |

**Supplemental Table 5. Gating strategy and analyzed molecules of each cell type (mouse)**

| Cell type | Gating strategy  (Common: FVD^-^ Single cell gating) | Analyzed molecules after gating |
| --- | --- | --- |
| Total cDCs  (In vivo) | MHC-II^+^CD11c^high^ | XCR1, CD103, CD11b |
| cDC1s  (In vivo) | MHC-II^+^CD11c^high^ XCR1^+^ | CD103, FCGR3  CD11c-i.v (Intravascular staining)  CD24, CLEC9A, CD8α (Spectral cytometry) |
| Total cDCs  (FL-DCs) | B220^–^MHC-II^+^CD11c^+^ | XCR1, CD103 |
| cDC1s  (FL-DCs) | B220^–^MHC-II^+^CD11c^high^ XCR1^+^ | CD86, CD80, CD40, MHC-I, CD61, CD38  CD103, FCGR3, DQ-OVA, CCR7 |
| CD8^+^ T | CD3^+^ CD8^+^ | CD44^high^CD62L^–^ (Activated T cells)  IL-9, IFN-γ (Intracellular staining) |
| CD4^+^ T | CD3^+^ CD4^+^ | Foxp3 (Intracelluar staining) |
| MDP* | Lin^–^*MHC-II^–^CD11c^–^CD135^+^CD115^+^CD117^+^ | ST2 |
| CDP* | Lin^–^MHC-II^–^CD11c^–^CD135^+^CD115^+^CD117^–^ | ST2 |
| Pre-DC | Lin^–^MHC-II^–^CD11c^+^CD135^+^ | ST2 |
| ILC | CD11c^–^Lin^–^CD127^+^ | ST2 |
| ILC2 | CD11c^–^Lin^–^CD127^+^CD25^+^ | ST2 (naïve BM cells and FL-DCs) |
| Basophil | CD11c^–^ FcεRIα^+^CD49b^+^ | **-** |
| Non-ILC  (FL-DCs) | CD11c^–^ Lin^–^CD127^-^ | **-** |
| CD49b- portion  (FL-DCs) | CD11c^–^ FcεRIα^+^CD49b^-^ | **-** |

*MDP: Macrophage/DC progenitor, *CDP: common DC progenitor, *Lin^-^ CD3/B220/CD11b/Ly6G (Ly6G is used in only *in vivo* cells).

**Supplemental Table 6. Gating strategy of each cell type (human)**

| Cell type | Gating strategy  (Common: FVD^-^ Single cell gating) |
| --- | --- |
| Monocyte (Classical) | CD3/CD16/CD19/CD56/CD66b^–^CD14^+^ |
| Lin^-^ cells | CD3/CD16/CD19/CD56/CD66b^–^CD14^–^ |
| CD8^+^ T | CD3^+^CD8^+^ |

**Reference**

1. Dominguez, D., Ye, C.. et al. Exogenous IL-33 Restores Dendritic Cell Activation and Maturation in Established Cancer. *J Immunol*. **198**, 1365-1375 (2017).

2. Lin, J. H., Huffman, A. P.. et al. Type 1 conventional dendritic cells are systemically dysregulated early in pancreatic carcinogenesis. *J Exp Med*. **217**, (2020).

3. Kim, S., Shen, T.. et al. Basophils can directly present or cross-present antigen to CD8 lymphocytes and alter CD8 T cell differentiation into IL-10-producing phenotypes. *J Immunol*. **183**, 3033-3039 (2009).

4. Ahmed, M. S., Byeon, S. E.. et al. Dab2, a negative regulator of DC immunogenicity, is an attractive molecular target for DC-based immunotherapy. *Oncoimmunology*. **4**, e984550 (2015).

5. Langmead, B., Salzberg, S. L. Fast gapped-read alignment with Bowtie 2. *Nat Methods*. **9**, 357-359 (2012).

6. Quinlan, A. R., Hall, I. M. BEDTools: a flexible suite of utilities for comparing genomic features. *Bioinformatics*. **26**, 841-842 (2010).

7. Chen, Y., Lun, A. T.. et al. From reads to genes to pathways: differential expression analysis of RNA-Seq experiments using Rsubread and the edgeR quasi-likelihood pipeline. *F1000Res*. **5**, 1438 (2016).

8. Schlitzer, A., Sivakamasundari, V.. et al. Identification of cDC1- and cDC2-committed DC progenitors reveals early lineage priming at the common DC progenitor stage in the bone marrow. *Nat Immunol*. **16**, 718-728 (2015).
