# Supplementary figures and images for: Discovery of highly immunogenic spleen-resident FCGR3+CD103+ cDC1s differentiated by IL-33-primed ST2+ basophils
Source: Cell Mol Immunol. 2023 May 29;20(7):820–34. doi: 10.1038/s41423-023-01035-8 (PMC10310784; doi:10.1038/s41423-023-01035-8)

## Slide 1
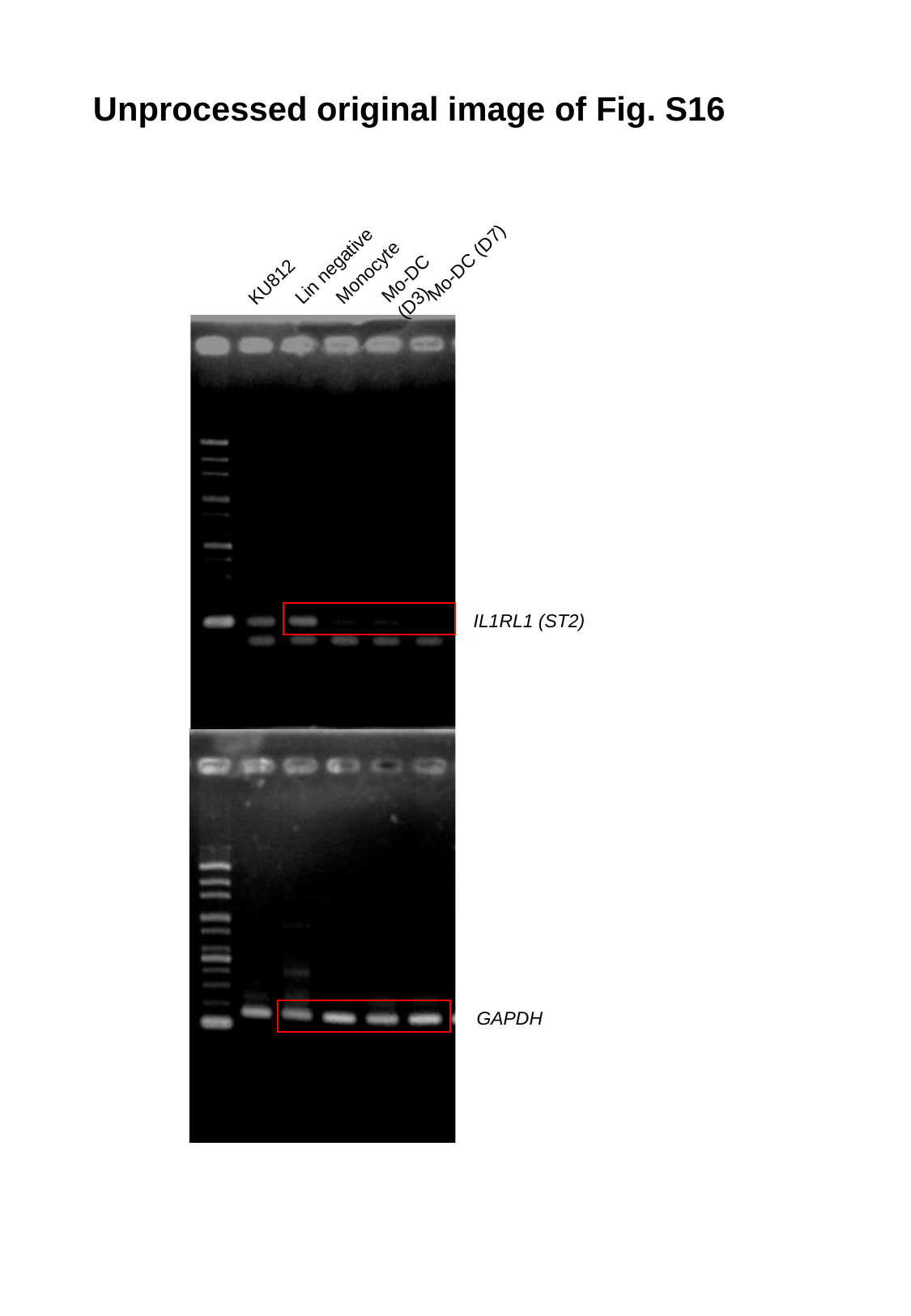

Unprocessed original image of Fig. S16
Mo-DC (D7)
Lin negative
Mo-DC (D3)
Monocyte
KU812
IL1RL1 (ST2)
GAPDH

Supplement: Supplementary file 2 — Original Image (Fig. S16) [file 41423_2023_1035_MOESM2_ESM.pptx]
