# Supplementary material for: Discovery of highly immunogenic spleen-resident FCGR3+CD103+ cDC1s differentiated by IL-33-primed ST2+ basophils
Source: Cell Mol Immunol. 2023 May 29;20(7):820–34. doi: 10.1038/s41423-023-01035-8 (PMC10310784; doi:10.1038/s41423-023-01035-8)
Supplement: Supplementary file 3 — List of differentially expressed genes [file 41423_2023_1035_MOESM3_ESM.docx]

**Supplemental Table 7. List of differentially expressed genes**

| **Gene symbol** | **Log_2_ Fold change**  **(FL-33 / FL-GM)** | **p-value** | **Description** |
| --- | --- | --- | --- |
| *Kif1a* | 4.247184134 | 0.0224391272451 | kinesin family member 1A |
| *Phactr1* | 4.217537329 | 0.0462659678534 | phosphatase and actin regulator 1 |
| *Frmd4b* | 4.074810693 | 0.0206290013435 | FERM domain containing 4B |
| *Slc30a4* | 3.696238459 | 0.0554947407262 | solute carrier family 30 (zinc transporter), member 4 |
| *Mgme1* | 3.692225262 | 0.0022880305584 | mitochondrial genome maintainance exonuclease 1 |
| *Socs1* | 3.66358595 | 0.0816136308614 | suppressor of cytokine signaling 1 |
| *Itgb3* | 3.65831915 | 0.0025586688238 | integrin beta 3 |
| *Fcgr3* | 3.600907763 | 0.1806655835968 | Fc receptor, IgG, low affinity III |
| *Sh3d21* | 3.527051391 | 0.0056789491362 | SH3 domain containing 21 |
| *Ptger2* | 3.430016424 | 0.1027887465577 | prostaglandin E receptor 2 (subtype EP2) |
| *Zc3h12c* | 3.418778207 | 0.0029554749595 | zinc finger CCCH type containing 12C |
| *Aig1* | 3.327625814 | 0.0002926271940 | androgen-induced 1 |
| *Wdr90* | 3.277180679 | 0.1406310683557 | WD repeat domain 90 |
| *Psrc1* | 3.276899724 | 0.1125622465566 | proline /serine-rich coiled-coil 1 |
| *Mapk13* | 3.229540218 | 0.0103286432021 | mitogen-activated protein kinase 13 |
| *Hddc2* | 3.183800416 | 0.1520041856096 | HD domain containing 2 |
| *Tada2a* | 3.182259265 | 0.0558189356853 | transcriptional adaptor 2A |
| *4931440P22Rik* | 3.152656247 | 0.0501890358727 | RIKEN cDNA 4931440P22 gene |
| *Pim3* | 3.140505801 | 0.1132170076254 | proviral integration site 3 |
| *Lpar1* | 3.079947643 | 0.1368291841114 | lysophosphatidic acid receptor 1 |
| *Gtse1* | 3.066764451 | 0.0076365659990 | G two S phase expressed protein 1 |
| *Prepl* | 3.052483953 | 0.0002025198218 | prolyl endopeptidase-like |
| *Rad18* | 3.037939484 | 0.0013109991124 | RAD18 E3 ubiquitin protein ligase |
| *Zfp930* | 3.013721337 | 0.1368303008397 | zinc finger protein 930 |
| *Sdc4* | 3.011075532 | 0.1621828548044 | syndecan 4 |
| *Man1a* | 2.910746273 | 0.0788080413924 | mannosidase 1, alpha |
| *Rrp12* | 2.879462755 | 0.1586490820234 | ribosomal RNA processing 12 homolog (S. cerevisiae) |
| *Rpgr* | 2.845208772 | 0.0000096638748 | retinitis pigmentosa GTPase regulator |
| *Pick1* | 2.819097379 | 0.1541927853215 | protein interacting with C kinase 1 |
| *Adcy6* | 2.775309274 | 0.0168716983181 | adenylate cyclase 6 |
| *Tube1* | 2.753831061 | 0.0739190319001 | epsilon-tubulin 1 |
| *4933408B17Rik* | 2.753831061 | 0.0739190319001 | RIKEN cDNA 4933408B17 gene |
| *Gm15787* | 2.749511718 | 0.0240147677316 | predicted gene 15787 |
| *Lgalsl* | 2.716148125 | 0.0432815339393 | lectin, galactoside binding-like |
| *Gch1* | 2.681767501 | 0.0539547081148 | GTP cyclohydrolase 1 |
| *Ntmt1* | 2.681334124 | 0.0136724117575 | N-terminal Xaa-Pro-Lys N-methyltransferase 1 |
| *Arhgap22* | 2.659164632 | 0.1329372941177 | Rho GTPase activating protein 22 |
| *Lctl* | 2.659069853 | 0.0127876223396 | lactase-like |
| *Pcid2* | 2.658270998 | 0.0093634953446 | PCI domain containing 2 |
| *Zfp799* | 2.657215265 | 0.0015885490322 | zinc finger protein 799 |
| *Runx2* | 2.647195848 | 0.0824617125097 | runt related transcription factor 2 |
| *Syngr3* | 2.634042228 | 0.1058359500618 | synaptogyrin 3 |
| *Atrnl1* | 2.622126311 | 0.1004520953306 | attractin like 1 |
| *Tbx21* | 2.615915417 | 0.0085038637869 | T-box 21 |
| *2810454H06Rik* | 2.612922388 | 0.0000620977618 | RIKEN cDNA 2810454H06 gene |
| *Wwc2* | 2.6044314 | 0.1837406826399 | WW, C2 and coiled-coil domain containing 2 |
| *Xxylt1* | 2.584590433 | 0.0503257445602 | xyloside xylosyltransferase 1 |
| *Il1b* | 2.578558126 | 0.0005672921717 | interleukin 1 beta |
| *Nup188* | 2.564339171 | 0.1166835366974 | nucleoporin 188 |
| *1600020E01Rik* | 2.540153618 | 0.0099583961582 | RIKEN cDNA 1600020E01 gene |
| *Mavs* | 2.536282303 | 0.1655740966486 | mitochondrial antiviral signaling protein |
| *Lrrc73* | 2.517710879 | 0.0354522355576 | leucine rich repeat containing 73 |
| *Slc27a4* | 2.507815552 | 0.0005087079818 | solute carrier family 27 (fatty acid transporter), member 4 |
| *Tfcp2* | 2.500791375 | 0.1224256221068 | transcription factor CP2 |
| *Tfpt* | 2.497339982 | 0.0435430887662 | TCF3 (E2A) fusion partner |
| *Trim45* | 2.465358492 | 0.0860450713201 | tripartite motif-containing 45 |
| *2610027K06Rik* | 2.431439972 | 0.1587333471050 | RIKEN cDNA 2610027K06 gene |
| *Atpaf2* | 2.418622341 | 0.0147527703097 | ATP synthase mitochondrial F1 complex assembly factor 2 |
| *Caskin2* | 2.407457392 | 0.0550085696146 | CASK-interacting protein 2 |
| *Tefm* | 2.395139192 | 0.0006862114043 | transcription elongation factor, mitochondrial |
| *1600002H07Rik* | 2.393220004 | 0.1849354334950 | RIKEN cDNA 1600002H07 gene |
| *Tgds* | 2.382714909 | 0.0366915019922 | TDP-glucose 4,6-dehydratase |
| *1300002E11Rik* | 2.375668774 | 0.0000964165760 | RIKEN cDNA 1300002E11 gene |
| *Nsl1* | 2.374200187 | 0.0344863838647 | NSL1, MIS12 kinetochore complex component |
| *Aifm2* | 2.364055114 | 0.0028977029221 | apoptosis-inducing factor, mitochondrion-associated 2 |
| *Zfp366* | 2.360893719 | 0.1139194894657 | zinc finger protein 366 |
| *Dgkh* | 2.348718035 | 0.0044611682196 | diacylglycerol kinase, eta |
| *Slc9a5* | 2.345647105 | 0.1567597398215 | solute carrier family 9 (sodium /hydrogen exchanger), member 5 |
| *Chsy3* | 2.324109639 | 0.1135221734769 | chondroitin sulfate synthase 3 |
| *Tmem132a* | 2.312717831 | 0.0574817973081 | transmembrane protein 132A |
| *Slco4a1* | 2.30956576 | 0.0708473573261 | solute carrier organic anion transporter family, member 4a1 |
| *Socs3* | 2.304228359 | 0.1391582041752 | suppressor of cytokine signaling 3 |
| *Gkap1* | 2.30273891 | 0.0338035554059 | G kinase anchoring protein 1 |
| *Ascc2* | 2.294632453 | 0.0794887978516 | activating signal cointegrator 1 complex subunit 2 |
| *Txndc11* | 2.284648159 | 0.0063571665298 | thioredoxin domain containing 11 |
| *Hcfc2* | 2.274168575 | 0.0065228313623 | host cell factor C2 |
| *A930019D19Rik* | 2.266025652 | 0.0659742016546 | RIKEN cDNA A930019D19 gene |
| *Ccdc162* | 2.264472189 | 0.1531125820212 | coiled-coil domain containing 162 |
| *Map9* | 2.252453363 | 0.0575351384583 | microtubule-associated protein 9 |
| *Pomgnt1* | 2.245299179 | 0.0268118481799 | protein O-linked mannose beta 1,2-N-acetylglucosaminyltransferase |
| *Sdr39u1* | 2.243912875 | 0.0390088912909 | short chain dehydrogenase /reductase family 39U, member 1 |
| *Hk3* | 2.239799679 | 0.0393928056633 | hexokinase 3 |
| *Naprt* | 2.237519384 | 0.1120828523859 | nicotinate phosphoribosyltransferase |
| *Pigw* | 2.229056411 | 0.1337850349244 | phosphatidylinositol glycan anchor biosynthesis, class W |
| *Gen1* | 2.218099066 | 0.0481832894130 | GEN1, Holliday junction 5' flap endonuclease |
| *Plgrkt* | 2.204554098 | 0.0473258073717 | plasminogen receptor, C-terminal lysine transmembrane protein |
| *Sprtn* | 2.202255327 | 0.0584159383245 | SprT-like N-terminal domain |
| *2900005J15Rik* | 2.201867108 | 0.0033250373983 | RIKEN cDNA 2900005J15 gene |
| *1700109H08Rik* | 2.193165089 | 0.0322640913225 | RIKEN cDNA 1700109H08 gene |
| *Zfp459* | 2.188748741 | 0.0956052958764 | zinc finger protein 459 |
| *Zfp3* | 2.174643481 | 0.0280622561320 | zinc finger protein 3 |
| *Ppp1r14a* | 2.172512283 | 0.0301536181207 | protein phosphatase 1, regulatory (inhibitor) subunit 14A |
| *Poc1a* | 2.159337096 | 0.0058323726024 | POC1 centriolar protein A |
| *Slc6a8* | 2.157916822 | 0.1216196747042 | solute carrier family 6 (neurotransmitter transporter, creatine), member 8 |
| *Muc6* | 2.148594818 | 0.0855634956673 | mucin 6, gastric |
| *Ddx31* | 2.14837525 | 0.0394487892667 | DEAD /H (Asp-Glu-Ala-Asp /His) box polypeptide 31 |
| *Ube2j2* | 2.144402176 | 0.1524814766094 | ubiquitin-conjugating enzyme E2J 2 |
| *Klf7* | 2.13471347 | 0.0791364031171 | Kruppel-like factor 7 (ubiquitous) |
| *Sirt4* | 2.13321502 | 0.0154049579671 | sirtuin 4 |
| *Scfd2* | 2.128425201 | 0.0670626252297 | Sec1 family domain containing 2 |
| *Ippk* | 2.120455198 | 0.1248312548929 | inositol 1,3,4,5,6-pentakisphosphate 2-kinase |
| *Aoah* | 2.118304527 | 0.1344687322899 | acyloxyacyl hydrolase |
| *Gemin4* | 2.110648231 | 0.0015306187009 | gem (nuclear organelle) associated protein 4 |
| *Megf8* | 2.110421136 | 0.0004020324753 | multiple EGF-like-domains 8 |
| *Ms4a6d* | 2.102462838 | 0.1651550156728 | membrane-spanning 4-domains, subfamily A, member 6D |
| *Tubb3* | 2.092751166 | 0.1201627491489 | tubulin, beta 3 class III |
| *Tmbim1* | 2.086596694 | 0.1867157539632 | transmembrane BAX inhibitor motif containing 1 |
| *Ier3* | 2.0838044 | 0.0354680853599 | immediate early response 3 |
| *Platr17* | 2.082346212 | 0.0173568290533 | pluripotency associated transcript 17 |
| *Hsd17b7* | 2.082077145 | 0.0218123609439 | hydroxysteroid (17-beta) dehydrogenase 7 |
| *Vta1* | 2.081535998 | 0.0799612084021 | vesicle (multivesicular body) trafficking 1 |
| *Slc16a14* | 2.078011771 | 0.0654627069730 | solute carrier family 16 (monocarboxylic acid transporters), member 14 |
| *Zfp810* | 2.075502181 | 0.1270779518182 | zinc finger protein 810 |
| *Mtrf1* | 2.075098133 | 0.0121170734685 | mitochondrial translational release factor 1 |
| *Snn* | 2.06004718 | 0.0558138702082 | stannin |
| *1110001J03Rik* | 2.041789389 | 0.0107287939049 | RIKEN cDNA 1110001J03 gene |
| *Setd6* | 2.036867304 | 0.0764382365358 | SET domain containing 6 |
| *Arhgap44* | 2.034367671 | 0.0514992405783 | Rho GTPase activating protein 44 |
| *Sirpa* | 2.034100103 | 0.0682699337469 | signal-regulatory protein alpha |
| *Rad9b* | 2.032598379 | 0.0419266120000 | RAD9 checkpoint clamp component B |
| *Nim1k* | 2.024502196 | 0.1185227950764 | NIM1 serine /threonine protein kinase |
| *Rnu11* | 2.024502196 | 0.1185227950764 | U11 small nuclear RNA |
| *Ccser1* | 2.010991134 | 0.0684241469846 | coiled-coil serine rich 1 |
| *Etfdh* | 2.007475225 | 0.1780349761964 | electron transferring flavoprotein, dehydrogenase |
| *Bcl2l11* | 2.002108228 | 0.1649777514579 | BCL2-like 11 (apoptosis facilitator) |
| *Gpr180* | 2.000926586 | 0.0343975562021 | G protein-coupled receptor 180 |
| *Htr7* | 1.991351984 | 0.0405055519256 | 5-hydroxytryptamine (serotonin) receptor 7 |
| *Yars* | 1.976217789 | 0.1829738192164 | tyrosyl-tRNA synthetase |
| *Ppp3cc* | 1.976098039 | 0.0357702288645 | protein phosphatase 3, catalytic subunit, gamma isoform |
| *Zfp493* | 1.976006355 | 0.0260449156956 | zinc finger protein 493 |
| *Tnfaip3* | 1.973789021 | 0.0524468095130 | tumor necrosis factor, alpha-induced protein 3 |
| *Efnb1* | 1.968286832 | 0.0828010654240 | ephrin B1 |
| *Ank1* | 1.960747268 | 0.0025667824195 | ankyrin 1, erythroid |
| *Fam3c* | 1.95996993 | 0.0174545672950 | family with sequence similarity 3, member C |
| *Matk* | 1.957756423 | 0.0079005617081 | megakaryocyte-associated tyrosine kinase |
| *Txnl4b* | 1.956765362 | 0.0121069837495 | thioredoxin-like 4B |
| *3110082I17Rik* | 1.953984796 | 0.1622742324642 | RIKEN cDNA 3110082I17 gene |
| *Snord65* | 1.945504558 | 0.0606574451004 | small nucleolar RNA, C /D box 65 |
| *Tmco3* | 1.938434532 | 0.1829115446598 | transmembrane and coiled-coil domains 3 |
| *Acsf3* | 1.935296617 | 0.0221041153016 | acyl-CoA synthetase family member 3 |
| *Exo1* | 1.930888651 | 0.0537578826509 | exonuclease 1 |
| *Ablim1* | 1.924308631 | 0.0929481945525 | actin-binding LIM protein 1 |
| *Mad2l2* | 1.923067257 | 0.1229575038933 | MAD2 mitotic arrest deficient-like 2 |
| *Fcho1* | 1.918709109 | 0.0489504417992 | FCH domain only 1 |
| *Fignl1* | 1.917627071 | 0.0425751785658 | fidgetin-like 1 |
| *Gm4349* | 1.903558158 | 0.0005074002094 | predicted gene 4349 |
| *2510009E07Rik* | 1.891689189 | 0.0907180625801 | RIKEN cDNA 2510009E07 gene |
| *Ppm1j* | 1.887815844 | 0.1348564803408 | protein phosphatase 1J |
| *Epg5* | 1.884827263 | 0.0681788908212 | ectopic P-granules autophagy protein 5 homolog (C. elegans) |
| *B230118H07Rik* | 1.881637416 | 0.0510257777185 | RIKEN cDNA B230118H07 gene |
| *Znhit3* | 1.88159538 | 0.1850792753868 | zinc finger, HIT type 3 |
| *Slc15a3* | 1.877581476 | 0.0941398858774 | solute carrier family 15, member 3 |
| *Ralgps1* | 1.874732602 | 0.1678763642185 | Ral GEF with PH domain and SH3 binding motif 1 |
| *Tcaim* | 1.871468497 | 0.0964651884479 | T cell activation inhibitor, mitochondrial |
| *Gtf2h2* | 1.870044798 | 0.1132332611286 | general transcription factor II H, polypeptide 2 |
| *Hist1h3e* | 1.866086637 | 0.0699989499158 | histone cluster 1, H3e |
| *Pgp* | 1.862083268 | 0.0654493599927 | phosphoglycolate phosphatase |
| *Slc2a9* | 1.861050015 | 0.1543757899251 | solute carrier family 2 (facilitated glucose transporter), member 9 |
| *Med18* | 1.857891445 | 0.1461036240435 | mediator complex subunit 18 |
| *Tjap1* | 1.855087255 | 0.0167378502897 | tight junction associated protein 1 |
| *Mtap* | 1.851115482 | 0.1217624627484 | methylthioadenosine phosphorylase |
| *A930024E05Rik* | 1.850631613 | 0.0850978062202 | RIKEN cDNA A930024E05 gene |
| *9930111J21Rik2* | 1.8460859 | 0.1941366445398 | RIKEN cDNA 9930111J21 gene 2 |
| *Cacna1a* | 1.845976847 | 0.1382540879939 | calcium channel, voltage-dependent, P /Q type, alpha 1A subunit |
| *Tspan4* | 1.841815041 | 0.0449902919609 | tetraspanin 4 |
| *Abcb6* | 1.832062126 | 0.0420360213213 | ATP-binding cassette, sub-family B (MDR /TAP), member 6 |
| *Nudt2* | 1.83144825 | 0.0699118691759 | nudix (nucleoside diphosphate linked moiety X)-type motif 2 |
| *1700066M21Rik* | 1.830769599 | 0.0871138898971 | RIKEN cDNA 1700066M21 gene |
| *Rbm14* | 1.823014639 | 0.1127200283999 | RNA binding motif protein 14 |
| *Casp6* | 1.822837438 | 0.0091609045245 | caspase 6 |
| *Snrpa1* | 1.822114992 | 0.0676494734449 | small nuclear ribonucleoprotein polypeptide A' |
| *Cyp20a1* | 1.821271038 | 0.1539486019906 | cytochrome P450, family 20, subfamily a, polypeptide 1 |
| *Odf2l* | 1.817712596 | 0.0557574465663 | outer dense fiber of sperm tails 2-like |
| *Ptpn1* | 1.81594058 | 0.0932787838087 | protein tyrosine phosphatase, non-receptor type 1 |
| *Emb* | 1.808330403 | 0.1527341656794 | embigin |
| *Jdp2* | 1.780922929 | 0.0640994293110 | Jun dimerization protein 2 |
| *Zfp707* | 1.779413397 | 0.1810475467388 | zinc finger protein 707 |
| *Tmem238* | 1.776534632 | 0.0804407648331 | transmembrane protein 238 |
| *4930592I03Rik* | 1.770067816 | 0.0471374811269 | RIKEN cDNA 4930592I03 gene |
| *Tmem38a* | 1.761506849 | 0.0038122869753 | transmembrane protein 38A |
| *Elp4* | 1.760956849 | 0.0607749681147 | elongator acetyltransferase complex subunit 4 |
| *Prdx4* | 1.757751433 | 0.0607484077787 | peroxiredoxin 4 |
| *Paxip1* | 1.744064615 | 0.1698011043388 | PAX interacting (with transcription-activation domain) protein 1 |
| *Gpatch3* | 1.739339725 | 0.0691906665411 | G patch domain containing 3 |
| *Mtx1* | 1.737133617 | 0.0550311961744 | metaxin 1 |
| *Mroh2a* | 1.736300394 | 0.1202157824617 | maestro heat-like repeat family member 2A |
| *Hfe* | 1.735907394 | 0.1611476608346 | hemochromatosis |
| *Ncstn* | 1.731547218 | 0.0751389987766 | nicastrin |
| *Slc11a2* | 1.728921345 | 0.1168497452736 | solute carrier family 11 (proton-coupled divalent metal ion transporters), member 2 |
| *Creld2* | 1.721198383 | 0.0672925081275 | cysteine-rich with EGF-like domains 2 |
| *Aldh18a1* | 1.717300897 | 0.1261605014952 | aldehyde dehydrogenase 18 family, member A1 |
| *Atp6v1g2* | 1.704620664 | 0.0545133850651 | ATPase, H+ transporting, lysosomal V1 subunit G2 |
| *Gm4737* | 1.703168024 | 0.1883439104934 | predicted gene 4737 |
| *Plcd3* | 1.693002713 | 0.0819463314023 | phospholipase C, delta 3 |
| *Gm14548* | 1.689723977 | 0.0567235762824 | predicted gene 14548 |
| *Tarsl2* | 1.688879757 | 0.0085914859578 | threonyl-tRNA synthetase-like 2 |
| *Gemin6* | 1.688213018 | 0.0009671467430 | gem (nuclear organelle) associated protein 6 |
| *Kctd7* | 1.685445437 | 0.0752688304927 | potassium channel tetramerisation domain containing 7 |
| *Snora31* | 1.682406527 | 0.0989347556616 | small nucleolar RNA, H /ACA box 31 |
| *Mtmr7* | 1.680563311 | 0.1415416421787 | myotubularin related protein 7 |
| *Uck2* | 1.677948371 | 0.1807724172506 | uridine-cytidine kinase 2 |
| *1810013L24Rik* | 1.676722624 | 0.0995142967181 | RIKEN cDNA 1810013L24 gene |
| *Fam98a* | 1.672362466 | 0.0152422138468 | family with sequence similarity 98, member A |
| *Plcg1* | 1.669355016 | 0.0485494652102 | phospholipase C, gamma 1 |
| *Fbxo22* | 1.662567813 | 0.0772728373726 | F-box protein 22 |
| *Hmga2-ps1* | 1.658204758 | 0.1627441392469 | high mobility group AT-hook 2, pseudogene 1 |
| *Dscc1* | 1.656252207 | 0.0741386281489 | DNA replication and sister chromatid cohesion 1 |
| *Fam109b* | 1.653524548 | 0.1361676940866 | family with sequence similarity 109, member B |
| *Qtrtd1* | 1.651930091 | 0.0936331549643 | queuine tRNA-ribosyltransferase domain containing 1 |
| *Galnt6* | 1.651835709 | 0.1285113419980 | UDP-N-acetyl-alpha-D-galactosamine:polypeptide N-acetylgalactosaminyltransferase 6 |
| *BC052040* | 1.651499598 | 0.1722745389740 | cDNA sequence BC052040 |
| *Elac2* | 1.638861645 | 0.0328309552714 | elaC ribonuclease Z 2 |
| *Coq7* | 1.634993863 | 0.0021830432830 | demethyl-Q 7 |
| *Strada* | 1.633246751 | 0.1823176532161 | STE20-related kinase adaptor alpha |
| *Dpy19l4* | 1.629006103 | 0.1005680937716 | dpy-19-like 4 (C. elegans) |
| *Gadd45g* | 1.62588695 | 0.0958679129955 | growth arrest and DNA-damage-inducible 45 gamma |
| *Asb14* | 1.622721855 | 0.0329622487158 | ankyrin repeat and SOCS box-containing 14 |
| *Zfp599* | 1.618201286 | 0.0429149105110 | zinc finger protein 599 |
| *Wdr92* | 1.615029849 | 0.0764929113184 | WD repeat domain 92 |
| *Scn2b* | 1.609943002 | 0.0014402778368 | sodium channel, voltage-gated, type II, beta |
| *Nudt5* | 1.601883066 | 0.0416989825367 | nudix (nucleoside diphosphate linked moiety X)-type motif 5 |
| *Sds* | 1.601730005 | 0.1799342589413 | serine dehydratase |
| *Serpinb6c* | 1.59788342 | 0.1251540557913 | serine (or cysteine) peptidase inhibitor, clade B, member 6c |
| *Shcbp1* | 1.594375188 | 0.1666853808290 | Shc SH2-domain binding protein 1 |
| *Spred2* | 1.589946326 | 0.1186498769244 | sprouty-related, EVH1 domain containing 2 |
| *Iba57* | 1.588789212 | 0.0122788804113 | IBA57 homolog, iron-sulfur cluster assembly |
| *2610318N02Rik* | 1.585823048 | 0.0128759827163 | RIKEN cDNA 2610318N02 gene |
| *Zfp429* | 1.584400556 | 0.0729969211480 | zinc finger protein 429 |
| *Nkapl* | 1.570063725 | 0.0206306538357 | NFKB activating protein-like |
| *Gpr19* | 1.570063725 | 0.0206306538357 | G protein-coupled receptor 19 |
| *Mcm10* | 1.564856236 | 0.0924706351378 | minichromosome maintenance 10 replication initiation factor |
| *Gm6182* | 1.557677184 | 0.0232487281649 | . |
| *Rint1* | 1.55576568 | 0.1296282698399 | RAD50 interactor 1 |
| *Mcm8* | 1.555653864 | 0.0993390430184 | minichromosome maintenance 8 homologous recombination repair factor |
| *Med6* | 1.554512389 | 0.1442795851672 | mediator complex subunit 6 |
| *Lsm1* | 1.553418717 | 0.1772316353083 | LSM1 homolog, mRNA degradation associated |
| *Adat2* | 1.552502946 | 0.0885205531588 | adenosine deaminase, tRNA-specific 2 |
| *Adamtsl4* | 1.548524728 | 0.0675501001983 | ADAMTS-like 4 |
| *Cox11* | 1.545215413 | 0.0445799959147 | cytochrome c oxidase assembly protein 11 |
| *Tmem263* | 1.543442572 | 0.1550583851187 | transmembrane protein 263 |
| *Tspan8* | 1.543428622 | 0.1688190050552 | tetraspanin 8 |
| *Rcn1* | 1.541697013 | 0.0128756255021 | reticulocalbin 1 |
| *Pex2* | 1.535674044 | 0.1651074716004 | peroxisomal biogenesis factor 2 |
| *Scamp4* | 1.534574759 | 0.0469321992711 | secretory carrier membrane protein 4 |
| *Mrps33* | 1.533105691 | 0.0552217855450 | mitochondrial ribosomal protein S33 |
| *Itprip* | 1.519988026 | 0.0752132609789 | inositol 1,4,5-triphosphate receptor interacting protein |
| *Iffo2* | 1.51706517 | 0.0190238933277 | intermediate filament family orphan 2 |
| *Tcta* | 1.516302706 | 0.0321595798369 | T cell leukemia translocation altered gene |
| *Vwa8* | 1.512378582 | 0.1481776038267 | von Willebrand factor A domain containing 8 |
| *Gnl3l* | 1.506710079 | 0.0759806935392 | guanine nucleotide binding protein-like 3 (nucleolar)-like |
| *Tmem254a* | 1.505496086 | 0.1877526587237 | transmembrane protein 254a |
| *Tmem254b* | 1.505496086 | 0.1877526587237 | transmembrane protein 254b |
| *Tmem254c* | 1.505496086 | 0.1877526587237 | transmembrane protein 254c |
| *2700097O09Rik* | 1.49620849 | 0.1272753982187 | RIKEN cDNA 2700097O09 gene |
| *Inpp4b* | 1.493312847 | 0.1068691615680 | inositol polyphosphate-4-phosphatase, type II |
| *Zfp59* | 1.492743545 | 0.0700680387675 | zinc finger protein 59 |
| *Gm8801* | 1.48828856 | 0.1896818633370 | predicted gene 8801 |
| *Dcp1a* | 1.488051851 | 0.1443932096064 | decapping mRNA 1A |
| *Snapin* | 1.487575209 | 0.0108493033964 | SNAP-associated protein |
| *Ak2* | 1.486521363 | 0.0633483729645 | adenylate kinase 2 |
| *Ubtd2* | 1.484237212 | 0.1105544926152 | ubiquitin domain containing 2 |
| *Pfdn6* | 1.482584436 | 0.0361863313104 | prefoldin subunit 6 |
| *Gtf2f1* | 1.480409363 | 0.0708282058344 | general transcription factor IIF, polypeptide 1 |
| *Cenpj* | 1.474499029 | 0.0905671417902 | centromere protein J |
| *Faim* | 1.472617327 | 0.1607003235579 | Fas apoptotic inhibitory molecule |
| *Zfp523* | 1.472369221 | 0.0987580927199 | zinc finger protein 523 |
| *Slc2a12* | 1.468689016 | 0.1163857429267 | solute carrier family 2 (facilitated glucose transporter), member 12 |
| *Fkbp5* | 1.466869465 | 0.0215859233353 | FK506 binding protein 5 |
| *Tubb2b* | 1.465526272 | 0.1173621054611 | tubulin, beta 2B class IIB |
| *Ints1* | 1.463771376 | 0.0195110576643 | integrator complex subunit 1 |
| *Zfp398* | 1.462682861 | 0.1317625001262 | zinc finger protein 398 |
| *Ctbs* | 1.460751154 | 0.0495806229293 | chitobiase, di-N-acetyl- |
| *Snrnp25* | 1.458243594 | 0.1041320287937 | small nuclear ribonucleoprotein 25 (U11 /U12) |
| *Arhgap6* | 1.452365475 | 0.1852351933744 | Rho GTPase activating protein 6 |
| *Arl15* | 1.451213471 | 0.1541275135922 | ADP-ribosylation factor-like 15 |
| *Nck1* | 1.446474185 | 0.1320418307459 | non-catalytic region of tyrosine kinase adaptor protein 1 |
| *Uxt* | 1.4450357 | 0.1265112489024 | ubiquitously expressed transcript |
| *Stt3b* | 1.444706451 | 0.1442457152435 | STT3, subunit of the oligosaccharyltransferase complex, homolog B (S. cerevisiae) |
| *Dars2* | 1.444500001 | 0.0478551729092 | aspartyl-tRNA synthetase 2 (mitochondrial) |
| *Suv39h2* | 1.440124141 | 0.0719271016860 | suppressor of variegation 3-9 homolog 2 (Drosophila) |
| *Nlrx1* | 1.438726203 | 0.1071488522689 | NLR family member X1 |
| *Actr8* | 1.437268191 | 0.0027190122723 | ARP8 actin-related protein 8 |
| *Rnf19b* | 1.435049462 | 0.0031481378477 | ring finger protein 19B |
| *Gab2* | 1.432528793 | 0.1129341252014 | growth factor receptor bound protein 2-associated protein 2 |
| *Ndufa12* | 1.432003476 | 0.0274824232561 | NADH dehydrogenase (ubiquinone) 1 alpha subcomplex, 12 |
| *Ptprcap* | 1.42899247 | 0.1662679450913 | protein tyrosine phosphatase, receptor type, C polypeptide-associated protein |
| *Wrnip1* | 1.428252245 | 0.0058859542114 | Werner helicase interacting protein 1 |
| *Nek2* | 1.427914494 | 0.1651373009085 | NIMA (never in mitosis gene a)-related expressed kinase 2 |
| *Arhgap10* | 1.425120672 | 0.0941037864199 | Rho GTPase activating protein 10 |
| *Sfn* | 1.422704076 | 0.0354001541008 | stratifin |
| *Ppil1* | 1.420595978 | 0.1573442141999 | peptidylprolyl isomerase (cyclophilin)-like 1 |
| *Mras* | 1.420377843 | 0.0094621664825 | muscle and microspikes RAS |
| *Napb* | 1.418279117 | 0.0052206797937 | N-ethylmaleimide sensitive fusion protein attachment protein beta |
| *Bora* | 1.414764653 | 0.0375561936581 | bora, aurora kinase A activator |
| *Thap6* | 1.410876152 | 0.0459042767044 | THAP domain containing 6 |
| *Pgk1* | 1.409172287 | 0.1740035582576 | phosphoglycerate kinase 1 |
| *Pde12* | 1.408370612 | 0.1904230781370 | phosphodiesterase 12 |
| *Adap2* | 1.406746869 | 0.1632611228896 | ArfGAP with dual PH domains 2 |
| *2410016O06Rik* | 1.399553907 | 0.0300848888605 | RIKEN cDNA 2410016O06 gene |
| *H2-DMb2* | 1.399504993 | 0.0040400481342 | histocompatibility 2, class II, locus Mb2 |
| *Ccdc92* | 1.397817115 | 0.1140829595780 | coiled-coil domain containing 92 |
| *Metrn* | 1.393403462 | 0.1985377878098 | meteorin, glial cell differentiation regulator |
| *Bbs5* | 1.391354754 | 0.1051037337099 | Bardet-Biedl syndrome 5 (human) |
| *Plekhh1* | 1.390355552 | 0.0549519204125 | pleckstrin homology domain containing, family H (with MyTH4 domain) member 1 |
| *Pigh* | 1.390355552 | 0.0549519204125 | phosphatidylinositol glycan anchor biosynthesis, class H |
| *Mapk11* | 1.389052421 | 0.1063850739698 | mitogen-activated protein kinase 11 |
| *Bahcc1* | 1.388765982 | 0.1928205675158 | BAH domain and coiled-coil containing 1 |
| *Atat1* | 1.388652098 | 0.1241215534884 | alpha tubulin acetyltransferase 1 |
| *Dpm1* | 1.383809703 | 0.0933722152848 | dolichol-phosphate (beta-D) mannosyltransferase 1 |
| *Bst1* | 1.381698282 | 0.0329401539959 | bone marrow stromal cell antigen 1 |
| *Tmem214* | 1.379119537 | 0.0195055414732 | transmembrane protein 214 |
| *Clp1* | 1.37830202 | 0.0446953495713 | CLP1, cleavage and polyadenylation factor I subunit |
| *Hist1h3c* | 1.372662641 | 0.1184846960077 | histone cluster 1, H3c |
| *Paip2b* | 1.371179271 | 0.0442079034835 | poly(A) binding protein interacting protein 2B |
| *Cdk10* | 1.361332142 | 0.0560293503075 | cyclin-dependent kinase 10 |
| *Batf* | 1.360360225 | 0.0105162893794 | basic leucine zipper transcription factor, ATF-like |
| *Taf6* | 1.359208728 | 0.1303117762953 | TATA-box binding protein associated factor 6 |
| *Golt1b* | 1.35649489 | 0.0798921467942 | golgi transport 1B |
| *Lrrc26* | 1.356448272 | 0.0000348137800 | leucine rich repeat containing 26 |
| *Mfsd8* | 1.356233092 | 0.1879621217900 | major facilitator superfamily domain containing 8 |
| *Gm14296* | 1.354091126 | 0.0060440658671 | predicted gene 14296 |
| *Abcb8* | 1.351773193 | 0.1036621780984 | ATP-binding cassette, sub-family B (MDR /TAP), member 8 |
| *Pcyox1l* | 1.344443373 | 0.0213543059477 | prenylcysteine oxidase 1 like |
| *Rpp30* | 1.339753987 | 0.1212527831327 | ribonuclease P /MRP 30 subunit |
| *Cdh23* | 1.338821404 | 0.1799209479065 | cadherin 23 (otocadherin) |
| *Cdyl* | 1.338550088 | 0.0865427681871 | chromodomain protein, Y chromosome-like |
| *Txnrd3* | 1.337028374 | 0.1249270402740 | thioredoxin reductase 3 |
| *Plp1* | 1.334071011 | 0.1356326725418 | proteolipid protein (myelin) 1 |
| *Fam196a* | 1.333812289 | 0.1747232195811 | family with sequence similarity 196, member A |
| *Itga6* | 1.333082367 | 0.0660817149132 | integrin alpha 6 |
| *Slc35b3* | 1.326811155 | 0.0249275095097 | solute carrier family 35, member B3 |
| *Toporsos* | 1.32658203 | 0.1491699648439 | topoisomerase I binding, arginine /serine-rich, opposite strand |
| *Chac2* | 1.323502443 | 0.1002932180530 | ChaC, cation transport regulator 2 |
| *Pusl1* | 1.319906083 | 0.1967539139904 | pseudouridylate synthase-like 1 |
| *Nmd3* | 1.318590629 | 0.0378707262927 | NMD3 ribosome export adaptor |
| *E2f1* | 1.316727445 | 0.1877479284527 | E2F transcription factor 1 |
| *Trim65* | 1.314978918 | 0.1988775200981 | tripartite motif-containing 65 |
| *Crls1* | 1.314842231 | 0.0029503430696 | cardiolipin synthase 1 |
| *Psmg1* | 1.314602939 | 0.1028320197953 | proteasome (prosome, macropain) assembly chaperone 1 |
| *Gm14391* | 1.314429915 | 0.0902927387437 | predicted gene 14391 |
| *Ppa1* | 1.313901613 | 0.0249466889279 | pyrophosphatase (inorganic) 1 |
| *Tex2* | 1.309970922 | 0.0715034778984 | testis expressed gene 2 |
| *Timm10* | 1.307530267 | 0.0944364605185 | translocase of inner mitochondrial membrane 10 |
| *Ppp2r3a* | 1.307410957 | 0.0875837162523 | protein phosphatase 2, regulatory subunit B'', alpha |
| *St3gal2* | 1.300270042 | 0.1887367830131 | ST3 beta-galactoside alpha-2,3-sialyltransferase 2 |
| *1700094D03Rik* | 1.299938369 | 0.0098495037661 | RIKEN cDNA 1700094D03 gene |
| *Ddx20* | 1.296484942 | 0.1621442183171 | DEAD (Asp-Glu-Ala-Asp) box polypeptide 20 |
| *Ccdc47* | 1.296438136 | 0.0548871615478 | coiled-coil domain containing 47 |
| *Pfdn1* | 1.289736209 | 0.1522955455297 | prefoldin 1 |
| *Ptgdr2* | 1.285115325 | 0.1221189474596 | prostaglandin D2 receptor 2 |
| *Kptn* | 1.284027993 | 0.1125184738148 | kaptin |
| *Cad* | 1.282836196 | 0.1436210425218 | carbamoyl-phosphate synthetase 2, aspartate transcarbamylase, and dihydroorotase |
| *Gchfr* | 1.275757019 | 0.0747778573981 | GTP cyclohydrolase I feedback regulator |
| *Erlin2* | 1.275326895 | 0.1399583893416 | ER lipid raft associated 2 |
| *Snx25* | 1.274996255 | 0.0186635578913 | sorting nexin 25 |
| *Tfrc* | 1.274781945 | 0.0022804518818 | transferrin receptor |
| *Bin2* | 1.274436982 | 0.0835863581702 | bridging integrator 2 |
| *Ankrd26* | 1.274109914 | 0.1370640547186 | ankyrin repeat domain 26 |
| *Slc7a7* | 1.271097656 | 0.1601036073944 | solute carrier family 7 (cationic amino acid transporter, y+ system), member 7 |
| *Cpne2* | 1.270664443 | 0.1824577329871 | copine II |
| *Tdg* | 1.270264023 | 0.0402971744357 | thymine DNA glycosylase |
| *Taf9b* | 1.267871734 | 0.0730201367795 | TATA-box binding protein associated factor 9B |
| *Ubtd1* | 1.267752569 | 0.0668023607175 | ubiquitin domain containing 1 |
| *Ulk1* | 1.263795906 | 0.1767028538966 | unc-51 like kinase 1 |
| *Ube2z* | 1.261580349 | 0.1358226220205 | ubiquitin-conjugating enzyme E2Z |
| *Mrpl11* | 1.260948603 | 0.0660000852801 | mitochondrial ribosomal protein L11 |
| *Eepd1* | 1.259795602 | 0.1958898122665 | endonuclease /exonuclease /phosphatase family domain containing 1 |
| *Aars* | 1.258623684 | 0.1790690327004 | alanyl-tRNA synthetase |
| *Slc12a9* | 1.257369397 | 0.0067332688962 | solute carrier family 12 (potassium /chloride transporters), member 9 |
| *Vps53* | 1.257177015 | 0.1353959042230 | vacuolar protein sorting 53 (yeast) |
| *Spryd3* | 1.2563018 | 0.1933588039882 | SPRY domain containing 3 |
| *Ppp6c* | 1.25355225 | 0.0628706516926 | protein phosphatase 6, catalytic subunit |
| *Mrto4* | 1.253327645 | 0.0307375908290 | mRNA turnover 4, ribosome maturation factor |
| *Tbc1d7* | 1.246847016 | 0.1414059270573 | TBC1 domain family, member 7 |
| *Smco4* | 1.246602959 | 0.1320386687470 | single-pass membrane protein with coiled-coil domains 4 |
| *Ahr* | 1.244411835 | 0.1442094312439 | aryl-hydrocarbon receptor |
| *Tmem170b* | 1.244113463 | 0.1838397282694 | transmembrane protein 170B |
| *Foxo4* | 1.239679004 | 0.1293539734280 | forkhead box O4 |
| *Nipsnap3b* | 1.237878115 | 0.0194588849332 | nipsnap homolog 3B (C. elegans) |
| *Ubb* | 1.234689285 | 0.1946656664980 | ubiquitin B |
| *Pard6b* | 1.232721858 | 0.1947132939267 | par-6 family cell polarity regulator beta |
| *1700092M07Rik* | 1.232043634 | 0.0257491445782 | RIKEN cDNA 1700092M07 gene |
| *Uchl4* | 1.228681796 | 0.1691913055399 | ubiquitin carboxyl-terminal esterase L4 |
| *Gm28042* | 1.227653878 | 0.1585671225096 | predicted gene, 28042 |
| *Dpp9* | 1.227398654 | 0.1332260326694 | dipeptidylpeptidase 9 |
| *Rnf103* | 1.227032734 | 0.1497350442919 | ring finger protein 103 |
| *Mlh1* | 1.223990379 | 0.1298913812286 | mutL homolog 1 |
| *Gm6710* | 1.223742757 | 0.0047908148318 | predicted gene 6710 |
| *Sept11* | 1.223033341 | 0.1000324508168 | septin 11 |
| *Rnpepl1* | 1.217458206 | 0.0649103402925 | arginyl aminopeptidase (aminopeptidase B)-like 1 |
| *Pprc1* | 1.216767449 | 0.0575428841788 | peroxisome proliferative activated receptor, gamma, coactivator-related 1 |
| *Mrpl35* | 1.214452207 | 0.0182482499914 | mitochondrial ribosomal protein L35 |
| *Orc6* | 1.21304167 | 0.0105687779130 | origin recognition complex, subunit 6 |
| *Plekhg2* | 1.212822542 | 0.1073595438745 | pleckstrin homology domain containing, family G (with RhoGef domain) member 2 |
| *Ubap2* | 1.204209775 | 0.0083130147365 | ubiquitin-associated protein 2 |
| *Memo1* | 1.202323926 | 0.1196159482982 | mediator of cell motility 1 |
| *Tmem161a* | 1.201634983 | 0.1805903253615 | transmembrane protein 161A |
| *Tspan5* | 1.200747995 | 0.1000108614305 | tetraspanin 5 |
| *Hprt* | 1.200651813 | 0.0527325042232 | hypoxanthine guanine phosphoribosyl transferase |
| *Cd24a* | 1.198937549 | 0.1200989079469 | CD24a antigen |
| *Rad51* | 1.196678841 | 0.0124441295973 | RAD51 recombinase |
| *Arl8a* | 1.192756608 | 0.0413308702867 | ADP-ribosylation factor-like 8A |
| *Glrx* | 1.192552433 | 0.0059353973420 | glutaredoxin |
| *Coq10b* | 1.188772432 | 0.0368182204421 | coenzyme Q10B |
| *Hist1h2bg* | 1.18807163 | 0.0481832894130 | histone cluster 1, H2bg |
| *Pbdc1* | 1.186144622 | 0.0500009132705 | polysaccharide biosynthesis domain containing 1 |
| *Mrpl28* | 1.185918035 | 0.1345578965972 | mitochondrial ribosomal protein L28 |
| *Olfm1* | 1.184137766 | 0.0050106271928 | olfactomedin 1 |
| *Pole2* | 1.182270578 | 0.0428043470408 | polymerase (DNA directed), epsilon 2 (p59 subunit) |
| *Cdc34* | 1.180061961 | 0.0192462216217 | cell division cycle 34 |
| *Zfp110* | 1.178048232 | 0.0062587405033 | zinc finger protein 110 |
| *Ndrg1* | 1.177294459 | 0.0989032728631 | N-myc downstream regulated gene 1 |
| *Ms4a6b* | 1.175016329 | 0.0060551398580 | membrane-spanning 4-domains, subfamily A, member 6B |
| *Mon1a* | 1.172272158 | 0.1624444407277 | MON1 homolog A, secretory traffciking associated |
| *Golph3* | 1.171513458 | 0.0849793302943 | golgi phosphoprotein 3 |
| *Ndufb7* | 1.17101973 | 0.0978827151244 | NADH dehydrogenase (ubiquinone) 1 beta subcomplex, 7 |
| *Elmod2* | 1.169899081 | 0.0488513524783 | ELMO /CED-12 domain containing 2 |
| *Gm15694* | 1.169652169 | 0.1486343961122 | predicted gene 15694 |
| *Setdb2* | 1.165677466 | 0.1388317596636 | SET domain, bifurcated 2 |
| *Tmsb15l* | 1.165017567 | 0.1292396209199 | thymosin beta 15b like |
| *Bcas2* | 1.164559076 | 0.1438529485307 | breast carcinoma amplified sequence 2 |
| *Hist1h2af* | 1.163926949 | 0.1367125591691 | histone cluster 1, H2af |
| *Tk2* | 1.160809218 | 0.1887657611502 | thymidine kinase 2, mitochondrial |
| *Zdhhc7* | 1.160390019 | 0.0553283180023 | zinc finger, DHHC domain containing 7 |
| *Sod2* | 1.156737697 | 0.0240121399722 | superoxide dismutase 2, mitochondrial |
| *Higd1a* | 1.155404737 | 0.0289155404500 | HIG1 domain family, member 1A |
| *0610010K14Rik* | 1.153653262 | 0.1389114269125 | RIKEN cDNA 0610010K14 gene |
| *Pfkfb2* | 1.147830787 | 0.0302400600159 | 6-phosphofructo-2-kinase /fructose-2,6-biphosphatase 2 |
| *Zfp51* | 1.147628738 | 0.1959171292817 | zinc finger protein 51 |
| *Emilin1* | 1.142717153 | 0.0791906775294 | elastin microfibril interfacer 1 |
| *0610010F05Rik* | 1.14036911 | 0.1465760258857 | RIKEN cDNA 0610010F05 gene |
| *Ggta1* | 1.139120482 | 0.0177855407934 | glycoprotein galactosyltransferase alpha 1, 3 |
| *Zdhhc18* | 1.139061362 | 0.1148999156810 | zinc finger, DHHC domain containing 18 |
| *S100pbp* | 1.138105261 | 0.0591278814998 | S100P binding protein |
| *Rqcd1* | 1.13659858 | 0.0379692156131 | . |
| *Tceb1* | 1.133992341 | 0.1134766220737 | transcription elongation factor B (SIII), polypeptide 1 |
| *Gas2l1* | 1.132461854 | 0.0192716664566 | growth arrest-specific 2 like 1 |
| *Psmb3* | 1.131592796 | 0.0179749719017 | proteasome (prosome, macropain) subunit, beta type 3 |
| *Kif18b* | 1.131342834 | 0.0493706608272 | kinesin family member 18B |
| *Chst14* | 1.131051334 | 0.1925847194065 | carbohydrate (N-acetylgalactosamine 4-0) sulfotransferase 14 |
| *Gmeb2* | 1.130372165 | 0.0798533901477 | glucocorticoid modulatory element binding protein 2 |
| *Pex13* | 1.127857685 | 0.1440618887576 | peroxisomal biogenesis factor 13 |
| *Zbed5* | 1.127348285 | 0.0643085859673 | zinc finger, BED type containing 5 |
| *Tmem135* | 1.12705919 | 0.0186055548557 | transmembrane protein 135 |
| *Rnf113a2* | 1.126584099 | 0.0025334624259 | ring finger protein 113A2 |
| *Utp23* | 1.126058051 | 0.1593741025636 | UTP23 small subunit processome component |
| *Zfp414* | 1.125030428 | 0.1901014145340 | zinc finger protein 414 |
| *Cdc6* | 1.124319416 | 0.0727231460032 | cell division cycle 6 |
| *Uba3* | 1.123069875 | 0.1912113794653 | ubiquitin-like modifier activating enzyme 3 |
| *Sgsm2* | 1.121592199 | 0.1956394835952 | small G protein signaling modulator 2 |
| *Cdt1* | 1.120574215 | 0.0288376382393 | chromatin licensing and DNA replication factor 1 |
| *Kctd20* | 1.118517641 | 0.1441335880425 | potassium channel tetramerisation domain containing 20 |
| *Asnsd1* | 1.118198939 | 0.1083667785761 | asparagine synthetase domain containing 1 |
| *Haus5* | 1.117812131 | 0.0164027620117 | HAUS augmin-like complex, subunit 5 |
| *Mrpl39* | 1.11710709 | 0.0975263262360 | mitochondrial ribosomal protein L39 |
| *Scfd1* | 1.112686898 | 0.0504300467064 | Sec1 family domain containing 1 |
| *Mrps12* | 1.110597025 | 0.1260256553493 | mitochondrial ribosomal protein S12 |
| *Pnp* | 1.109806519 | 0.1551434641522 | purine-nucleoside phosphorylase |
| *B3gnt5* | 1.106663508 | 0.1583040150094 | UDP-GlcNAc:betaGal beta-1,3-N-acetylglucosaminyltransferase 5 |
| *Ndufaf3* | 1.104361071 | 0.0640505618930 | NADH dehydrogenase (ubiquinone) 1 alpha subcomplex, assembly factor 3 |
| *Foxred1* | 1.098675999 | 0.1721036991544 | FAD-dependent oxidoreductase domain containing 1 |
| *Ap1s1* | 1.097632603 | 0.0084421471816 | adaptor protein complex AP-1, sigma 1 |
| *Rfc4* | 1.096618137 | 0.0187341349390 | replication factor C (activator 1) 4 |
| *Gfra2* | 1.095890444 | 0.1191702161940 | glial cell line derived neurotrophic factor family receptor alpha 2 |
| *Rrp15* | 1.093753994 | 0.0593923059478 | ribosomal RNA processing 15 homolog (S. cerevisiae) |
| *Tmem185b* | 1.092551363 | 0.1321392438871 | transmembrane protein 185B |
| *Tax1bp1* | 1.091220008 | 0.0454822043228 | Tax1 (human T cell leukemia virus type I) binding protein 1 |
| *Tsen54* | 1.087689705 | 0.1061624012081 | tRNA splicing endonuclease subunit 54 |
| *Sec14l1* | 1.086429613 | 0.0837111106906 | SEC14-like lipid binding 1 |
| *Nme1* | 1.085273352 | 0.0449082236850 | NME /NM23 nucleoside diphosphate kinase 1 |
| *Vwa1* | 1.084980866 | 0.1510956118096 | von Willebrand factor A domain containing 1 |
| *Pisd* | 1.082041796 | 0.1087710832799 | phosphatidylserine decarboxylase |
| *S1pr4* | 1.080941837 | 0.1899066743377 | sphingosine-1-phosphate receptor 4 |
| *Auh* | 1.074415125 | 0.0176877230380 | AU RNA binding protein /enoyl-coenzyme A hydratase |
| *Ska1* | 1.074116627 | 0.1974103350329 | spindle and kinetochore associated complex subunit 1 |
| *Zfp771* | 1.073157815 | 0.1675158219820 | zinc finger protein 771 |
| *2310039H08Rik* | 1.071890436 | 0.1270868938123 | RIKEN cDNA 2310039H08 gene |
| *Dda1* | 1.070026092 | 0.1645926198684 | DET1 and DDB1 associated 1 |
| *Mcoln2* | 1.069035733 | 0.1632042592606 | mucolipin 2 |
| *Eif4ebp1* | 1.067959866 | 0.0332388770959 | eukaryotic translation initiation factor 4E binding protein 1 |
| *Mrpl42* | 1.067130506 | 0.0975718379968 | mitochondrial ribosomal protein L42 |
| *Ndc1* | 1.065885074 | 0.1175539519203 | NDC1 transmembrane nucleoporin |
| *Polr3h* | 1.063316981 | 0.0038406232537 | polymerase (RNA) III (DNA directed) polypeptide H |
| *Gart* | 1.060639409 | 0.1276648628756 | phosphoribosylglycinamide formyltransferase |
| *Ube2n* | 1.060145188 | 0.1372745784228 | ubiquitin-conjugating enzyme E2N |
| *Mettl5* | 1.059623045 | 0.1159218829651 | methyltransferase like 5 |
| *Mthfsl* | 1.057629879 | 0.1257603655691 | 5, 10-methenyltetrahydrofolate synthetase-like |
| *Hexim1* | 1.055199879 | 0.0618581179910 | hexamethylene bis-acetamide inducible 1 |
| *Slc25a32* | 1.053716968 | 0.0229159403213 | solute carrier family 25, member 32 |
| *Cep78* | 1.052032474 | 0.1691684391591 | centrosomal protein 78 |
| *Scd1* | 1.05065847 | 0.1453957534669 | stearoyl-Coenzyme A desaturase 1 |
| *Chaf1a* | 1.048756985 | 0.0615562877143 | chromatin assembly factor 1, subunit A (p150) |
| *Cyb561d2* | 1.045484172 | 0.0733058768202 | cytochrome b-561 domain containing 2 |
| *Htt* | 1.044607869 | 0.1984873399558 | huntingtin |
| *H6pd* | 1.042181571 | 0.0682521470568 | hexose-6-phosphate dehydrogenase (glucose 1-dehydrogenase) |
| *Mrpl27* | 1.037858365 | 0.1470880195921 | mitochondrial ribosomal protein L27 |
| *Marveld2* | 1.037304057 | 0.1751348218199 | MARVEL (membrane-associating) domain containing 2 |
| *Bpnt1* | 1.034744489 | 0.0854801106930 | bisphosphate 3'-nucleotidase 1 |
| *Sec13* | 1.034096494 | 0.1522872485646 | SEC13 homolog, nuclear pore and COPII coat complex component |
| *Tomm40* | 1.033270306 | 0.1778579611351 | translocase of outer mitochondrial membrane 40 homolog (yeast) |
| *Cenpa* | 1.031983893 | 0.0619944738683 | centromere protein A |
| *Nup85* | 1.030659861 | 0.1405625830602 | nucleoporin 85 |
| *Zfp160* | 1.030474217 | 0.0241296063912 | zinc finger protein 160 |
| *Lin54* | 1.029436845 | 0.0057417597125 | lin-54 homolog (C. elegans) |
| *Zdhhc2* | 1.029387395 | 0.0002660607727 | zinc finger, DHHC domain containing 2 |
| *Oat* | 1.027005905 | 0.0444579732093 | ornithine aminotransferase |
| *Cep57l1* | 1.026236712 | 0.0432065343842 | centrosomal protein 57-like 1 |
| *Pim2* | 1.02468383 | 0.1900197455143 | proviral integration site 2 |
| *Rnmtl1* | 1.022021825 | 0.0256623654195 | RNA methyltransferase like 1 |
| *Camk2g* | 1.02198219 | 0.1374561937999 | calcium /calmodulin-dependent protein kinase II gamma |
| *Pim1* | 1.021481044 | 0.0674121173775 | proviral integration site 1 |
| *Rhpn2* | 1.01939807 | 0.0513619638028 | rhophilin, Rho GTPase binding protein 2 |
| *E2f3* | 1.014916214 | 0.0829301625953 | E2F transcription factor 3 |
| *Cdca7* | 1.013215463 | 0.0485845702186 | cell division cycle associated 7 |
| *Cdk5* | 1.012667392 | 0.1741320079198 | cyclin-dependent kinase 5 |
| *Zc3h6* | 1.009427505 | 0.0937681960157 | zinc finger CCCH type containing 6 |
| *Cdc73* | 1.009086922 | 0.0052916692085 | cell division cycle 73, Paf1 /RNA polymerase II complex component |
| *Abhd8* | 1.008940494 | 0.1773226087609 | abhydrolase domain containing 8 |
| *Tmem88* | 1.007766855 | 0.0835693921902 | transmembrane protein 88 |
| *Slc41a3* | 1.003764405 | 0.1524804936635 | solute carrier family 41, member 3 |
| *Psmd6* | 1.003440478 | 0.0141685038922 | proteasome (prosome, macropain) 26S subunit, non-ATPase, 6 |
| *Mcm4* | 1.00270855 | 0.0418936494373 | minichromosome maintenance complex component 4 |
| *Rab24* | 1.002014999 | 0.1893407222605 | RAB24, member RAS oncogene family |
| *Pou2f2* | 1.000981647 | 0.1263012856614 | POU domain, class 2, transcription factor 2 |
| *Gbas* | -1.000233477 | 0.1022451504597 | glioblastoma amplified sequence |
| *Rasl11b* | -1.000399861 | 0.1839087228903 | RAS-like, family 11, member B |
| *Tnfrsf13b* | -1.003049787 | 0.1865251585545 | tumor necrosis factor receptor superfamily, member 13b |
| *Cadm1* | -1.004890405 | 0.0897985268061 | cell adhesion molecule 1 |
| *Mink1* | -1.006586301 | 0.0425622728829 | misshapen-like kinase 1 (zebrafish) |
| *Edrf1* | -1.00668634 | 0.0481345856535 | erythroid differentiation regulatory factor 1 |
| *Aste1* | -1.009934943 | 0.0801822090846 | asteroid homolog 1 (Drosophila) |
| *Surf6* | -1.010450562 | 0.0408514010515 | surfeit gene 6 |
| *Bmpr2* | -1.01052288 | 0.0668278370991 | bone morphogenetic protein receptor, type II (serine /threonine kinase) |
| *Mast4* | -1.012469875 | 0.0963689799395 | microtubule associated serine /threonine kinase family member 4 |
| *Zfp296* | -1.014536891 | 0.0423398841196 | zinc finger protein 296 |
| *Cks2* | -1.014628447 | 0.0446482629521 | CDC28 protein kinase regulatory subunit 2 |
| *Fzd5* | -1.01619911 | 0.0428574088118 | frizzled class receptor 5 |
| *Mynn* | -1.016560043 | 0.0000695313664 | myoneurin |
| *Ccbl2* | -1.019120546 | 0.1328121038070 | . |
| *Ust* | -1.022021237 | 0.0552605974377 | uronyl-2-sulfotransferase |
| *Atraid* | -1.024531877 | 0.0455580285995 | all-trans retinoic acid induced differentiation factor |
| *Rybp* | -1.02465932 | 0.1104498846311 | RING1 and YY1 binding protein |
| *Stx3* | -1.024983892 | 0.1443282096122 | syntaxin 3 |
| *Tcirg1* | -1.025262456 | 0.1947035089414 | T cell, immune regulator 1, ATPase, H+ transporting, lysosomal V0 protein A3 |
| *Sh3kbp1* | -1.026471942 | 0.1351793986306 | SH3-domain kinase binding protein 1 |
| *Nfxl1* | -1.027159222 | 0.1294768584591 | nuclear transcription factor, X-box binding-like 1 |
| *Uqcc3* | -1.027241004 | 0.0886479026851 | ubiquinol-cytochrome c reductase complex assembly factor 3 |
| *Gt(ROSA)26Sor* | -1.030201998 | 0.1699563553213 | gene trap ROSA 26, Philippe Soriano |
| *Ptprs* | -1.030253858 | 0.1617377437121 | protein tyrosine phosphatase, receptor type, S |
| *Nedd4l* | -1.031136367 | 0.0246430473063 | neural precursor cell expressed, developmentally down-regulated gene 4-like |
| *Dnajc18* | -1.032061186 | 0.0214996995996 | DnaJ heat shock protein family (Hsp40) member C18 |
| *Dapk1* | -1.032259404 | 0.1873896448922 | death associated protein kinase 1 |
| *Dis3l2* | -1.032685505 | 0.1940199517228 | DIS3 like 3'-5' exoribonuclease 2 |
| *Dock11* | -1.033208137 | 0.0387490990609 | dedicator of cytokinesis 11 |
| *Tdrd7* | -1.033390468 | 0.1663917563649 | tudor domain containing 7 |
| *Zdhhc14* | -1.034843079 | 0.0871756756912 | zinc finger, DHHC domain containing 14 |
| *Rmnd5b* | -1.035321128 | 0.0220598541717 | required for meiotic nuclear division 5 homolog B |
| *Gpr87* | -1.039133472 | 0.0944509793322 | G protein-coupled receptor 87 |
| *Mterf2* | -1.041655663 | 0.0598147244685 | mitochondrial transcription termination factor 2 |
| *Calhm2* | -1.042557223 | 0.0053919060337 | calcium homeostasis modulator 2 |
| *6330416G13Rik* | -1.04287132 | 0.1010561330400 | RIKEN cDNA 6330416G13 gene |
| *Tmem19* | -1.043414257 | 0.0335055437468 | transmembrane protein 19 |
| *St6galnac2* | -1.043781011 | 0.1955145181969 | ST6 (alpha-N-acetyl-neuraminyl-2,3-beta-galactosyl-1, 3)-N-acetylgalactosaminide alpha-2,6-sialyltransferase 2 |
| *4930413G21Rik* | -1.043905516 | 0.1858365394285 | RIKEN cDNA 4930413G21 gene |
| *Gpx2-ps1* | -1.043985023 | 0.1076621338660 | glutathione peroxidase 2, pseudogene 1 |
| *Msra* | -1.044237451 | 0.0938102958053 | methionine sulfoxide reductase A |
| *Fig4* | -1.044517697 | 0.0251132894349 | FIG4 phosphoinositide 5-phosphatase |
| *Rinl* | -1.045416874 | 0.0139472877809 | Ras and Rab interactor-like |
| *Il3ra* | -1.046226437 | 0.0652084072730 | interleukin 3 receptor, alpha chain |
| *Stard5* | -1.046606002 | 0.1851867179812 | StAR-related lipid transfer (START) domain containing 5 |
| *Cerk* | -1.051229406 | 0.1860984639328 | ceramide kinase |
| *Amz2* | -1.052730544 | 0.1173771920827 | archaelysin family metallopeptidase 2 |
| *Atg14* | -1.055467536 | 0.0876067484016 | autophagy related 14 |
| *Havcr2* | -1.055869689 | 0.1756294087549 | hepatitis A virus cellular receptor 2 |
| *Slc22a5* | -1.05717965 | 0.0481885373808 | solute carrier family 22 (organic cation transporter), member 5 |
| *Dok2* | -1.057345265 | 0.0653014827820 | docking protein 2 |
| *Ager* | -1.058674531 | 0.1167689967146 | advanced glycosylation end product-specific receptor |
| *Crebrf* | -1.060585011 | 0.0254329095694 | CREB3 regulatory factor |
| *Naa40* | -1.060946557 | 0.1655804208797 | N(alpha)-acetyltransferase 40, NatD catalytic subunit |
| *Trp53i13* | -1.061137325 | 0.0869999200817 | transformation related protein 53 inducible protein 13 |
| *Adgre5* | -1.062055971 | 0.1470417118327 | adhesion G protein-coupled receptor E5 |
| *Ankle1* | -1.062423354 | 0.1078764836708 | ankyrin repeat and LEM domain containing 1 |
| *Lrrc61* | -1.065822319 | 0.1223542924804 | leucine rich repeat containing 61 |
| *Pink1* | -1.066712751 | 0.1210350543149 | PTEN induced putative kinase 1 |
| *Dnah11* | -1.067870343 | 0.1449817630612 | dynein, axonemal, heavy chain 11 |
| *Aim1* | -1.072993426 | 0.0931103042187 | absent in melanoma 1 |
| *Rogdi* | -1.073347691 | 0.1321346498530 | rogdi homolog |
| *Ric8b* | -1.078089906 | 0.0436998709547 | RIC8 guanine nucleotide exchange factor B |
| *Gpn3* | -1.080062683 | 0.0114130610928 | GPN-loop GTPase 3 |
| *Card10* | -1.080240066 | 0.0052324259690 | caspase recruitment domain family, member 10 |
| *1810026B05Rik* | -1.080493153 | 0.1233981948601 | RIKEN cDNA 1810026B05 gene |
| *D5Ertd579e* | -1.089776706 | 0.1016202439466 | DNA segment, Chr 5, ERATO Doi 579, expressed |
| *Fam210b* | -1.090726324 | 0.0366430560705 | family with sequence similarity 210, member B |
| *Snx30* | -1.091881946 | 0.0043673641598 | sorting nexin family member 30 |
| *Lyz2* | -1.094316554 | 0.1592829788839 | lysozyme 2 |
| *Adk* | -1.095865786 | 0.0810448269578 | adenosine kinase |
| *Mis18bp1* | -1.09668338 | 0.0735591423849 | MIS18 binding protein 1 |
| *Blmh* | -1.099746888 | 0.0885522536606 | bleomycin hydrolase |
| *Zbtb18* | -1.102835743 | 0.0586557950869 | zinc finger and BTB domain containing 18 |
| *Tigd2* | -1.103383417 | 0.0057689644988 | tigger transposable element derived 2 |
| *Sgk1* | -1.107422794 | 0.0123498706918 | serum /glucocorticoid regulated kinase 1 |
| *Zfp950* | -1.107954696 | 0.0340852969009 | zinc finger protein 950 |
| *Kit* | -1.108479967 | 0.0677580120890 | kit oncogene |
| *Hiat1* | -1.11124158 | 0.0358442763490 | . |
| *Abcb1a* | -1.111664878 | 0.0451499534010 | ATP-binding cassette, sub-family B (MDR /TAP), member 1A |
| *Zmym5* | -1.115247411 | 0.0398104384835 | zinc finger, MYM-type 5 |
| *Vps37b* | -1.11596176 | 0.0065904119788 | vacuolar protein sorting 37B |
| *Trit1* | -1.116447715 | 0.1321425922756 | tRNA isopentenyltransferase 1 |
| *5730508B09Rik* | -1.117280358 | 0.1073356362721 | RIKEN cDNA 5730508B09 gene |
| *Phlpp1* | -1.117523814 | 0.0517241287292 | PH domain and leucine rich repeat protein phosphatase 1 |
| *Map4k2* | -1.119488542 | 0.1572302984779 | mitogen-activated protein kinase kinase kinase kinase 2 |
| *Otud7b* | -1.121610463 | 0.1307362151763 | OTU domain containing 7B |
| *Plec* | -1.132089749 | 0.0843293818913 | plectin |
| *Arsb* | -1.136787985 | 0.1642589045682 | arylsulfatase B |
| *Nprl2* | -1.138397233 | 0.0425127969489 | nitrogen permease regulator-like 2 |
| *BC029722* | -1.139130439 | 0.0931883073414 | cDNA sequence BC029722 |
| *Ppap2a* | -1.145346962 | 0.0496795826795 | . |
| *Zfp111* | -1.146740996 | 0.0959412712853 | zinc finger protein 111 |
| *Lztr1* | -1.147133314 | 0.0734410969294 | leucine-zipper-like transcriptional regulator, 1 |
| *Tank* | -1.147763823 | 0.1850705447408 | TRAF family member-associated Nf-kappa B activator |
| *Enpp4* | -1.14903062 | 0.0164044182090 | ectonucleotide pyrophosphatase /phosphodiesterase 4 |
| *Obfc1* | -1.153605018 | 0.1509043971588 | oligonucleotide /oligosaccharide-binding fold containing 1 |
| *BC017158* | -1.153669965 | 0.1573449201138 | cDNA sequence BC017158 |
| *Prkab2* | -1.155554714 | 0.0068362391668 | protein kinase, AMP-activated, beta 2 non-catalytic subunit |
| *Zfp568* | -1.156994671 | 0.0804794102125 | zinc finger protein 568 |
| *Gm8615* | -1.157768963 | 0.0503527232928 | predicted pseudogene 8615 |
| *Plk4* | -1.162086715 | 0.0002290100412 | polo-like kinase 4 |
| *Ocrl* | -1.163228311 | 0.0830663410570 | oculocerebrorenal syndrome of Lowe |
| *Stat5a* | -1.163262803 | 0.0585444091670 | signal transducer and activator of transcription 5A |
| *Sufu* | -1.164959348 | 0.0854196191576 | suppressor of fused homolog (Drosophila) |
| *Raph1* | -1.165210168 | 0.0495261554225 | Ras association (RalGDS /AF-6) and pleckstrin homology domains 1 |
| *Cask* | -1.165309438 | 0.0772130177873 | calcium /calmodulin-dependent serine protein kinase (MAGUK family) |
| *Prrg2* | -1.171355263 | 0.0155883366577 | proline-rich Gla (G-carboxyglutamic acid) polypeptide 2 |
| *Pi4ka* | -1.172031206 | 0.1587897112341 | phosphatidylinositol 4-kinase, catalytic, alpha polypeptide |
| *Prr3* | -1.172487132 | 0.1458634157402 | proline-rich polypeptide 3 |
| *Tbck* | -1.174006967 | 0.0469196076855 | TBC1 domain containing kinase |
| *Zfp263* | -1.17820841 | 0.1575682428176 | zinc finger protein 263 |
| *Acot2* | -1.181686899 | 0.1931313599971 | acyl-CoA thioesterase 2 |
| *Gcat* | -1.181706996 | 0.1230945511466 | glycine C-acetyltransferase (2-amino-3-ketobutyrate-coenzyme A ligase) |
| *Nhlrc3* | -1.185264296 | 0.0934503105531 | NHL repeat containing 3 |
| *Prkd2* | -1.187035689 | 0.0686577901919 | protein kinase D2 |
| *Il17ra* | -1.190549804 | 0.0951238735013 | interleukin 17 receptor A |
| *Adam22* | -1.19055452 | 0.1570577139365 | a disintegrin and metallopeptidase domain 22 |
| *Slc52a2* | -1.192310824 | 0.1761259010005 | solute carrier protein 52, member 2 |
| *Haus8* | -1.192824597 | 0.0804568620580 | 4HAUS augmin-like complex, subunit 8 |
| *Acss1* | -1.20061111 | 0.0653390870880 | acyl-CoA synthetase short-chain family member 1 |
| *Cryzl1* | -1.201810791 | 0.1692338112116 | crystallin, zeta (quinone reductase)-like 1 |
| *Oxr1* | -1.202841728 | 0.0401393399856 | oxidation resistance 1 |
| *Gmppa* | -1.20347964 | 0.0423590886216 | GDP-mannose pyrophosphorylase A |
| *Upf3a* | -1.203588971 | 0.0608750250052 | UPF3 regulator of nonsense transcripts homolog A (yeast) |
| *Gm867* | -1.208761162 | 0.1318821384331 | predicted gene 867 |
| *Gm13157* | -1.209595306 | 0.0209752560122 | predicted gene 13157 |
| *Lztfl1* | -1.210181533 | 0.1632897962862 | leucine zipper transcription factor-like 1 |
| *Msi2* | -1.217679064 | 0.0299628763303 | musashi RNA-binding protein 2 |
| *Cfl2* | -1.217859505 | 0.0545486483015 | cofilin 2, muscle |
| *Dennd1a* | -1.218783002 | 0.1470625502895 | DENN /MADD domain containing 1A |
| *Crim1* | -1.219628167 | 0.0776692554331 | cysteine rich transmembrane BMP regulator 1 (chordin like) |
| *2410006H16Rik* | -1.225610918 | 0.0864159078288 | RIKEN cDNA 2410006H16 gene |
| *Mmp24* | -1.226187433 | 0.0650842548332 | matrix metallopeptidase 24 |
| *Scai* | -1.227144853 | 0.1426231571158 | suppressor of cancer cell invasion |
| *Atp6v0a1* | -1.230923682 | 0.1507621354658 | ATPase, H+ transporting, lysosomal V0 subunit A1 |
| *Mcoln1* | -1.231507875 | 0.1659484464374 | mucolipin 1 |
| *Plekha8* | -1.23506599 | 0.1123757479192 | pleckstrin homology domain containing, family A (phosphoinositide binding specific) member 8 |
| *Tmem164* | -1.237815891 | 0.1600078773239 | transmembrane protein 164 |
| *Rrad* | -1.23871906 | 0.1794322581024 | Ras-related associated with diabetes |
| *Wdr7* | -1.238788078 | 0.1230335807972 | WD repeat domain 7 |
| *Psd* | -1.238846185 | 0.0128588982286 | pleckstrin and Sec7 domain containing |
| *Mdk* | -1.241607843 | 0.1410514039592 | midkine |
| *Spice1* | -1.243332354 | 0.0762195782709 | spindle and centriole associated protein 1 |
| *Lmna* | -1.245627398 | 0.0903012917317 | lamin A |
| *Slc16a4* | -1.247075501 | 0.1352425373467 | solute carrier family 16 (monocarboxylic acid transporters), member 4 |
| *Nrde2* | -1.250642649 | 0.1992852170251 | nrde-2 necessary for RNA interference, domain containing |
| *Cpd* | -1.250731667 | 0.1496729575749 | carboxypeptidase D |
| *Traf1* | -1.25437655 | 0.1844467693691 | TNF receptor-associated factor 1 |
| *Ndufaf2* | -1.260855192 | 0.1574936349401 | NADH dehydrogenase (ubiquinone) 1 alpha subcomplex, assembly factor 2 |
| *Lss* | -1.26104326 | 0.1651593850513 | lanosterol synthase |
| *Ybey* | -1.261986409 | 0.1848648767921 | ybeY metallopeptidase |
| *Rad52* | -1.262956279 | 0.1817012943480 | RAD52 homolog, DNA repair protein |
| *Lrrc14* | -1.263384013 | 0.1636303315014 | leucine rich repeat containing 14 |
| *Gtf2e1* | -1.266668918 | 0.1509204403896 | general transcription factor II E, polypeptide 1 (alpha subunit) |
| *Rgp1* | -1.26973567 | 0.1752651674576 | RAB6A GEF compex partner 1 |
| *Bend6* | -1.271269151 | 0.1785981388044 | BEN domain containing 6 |
| *Zfp563* | -1.272879752 | 0.0426425859295 | zinc finger protein 563 |
| *Myo10* | -1.277897811 | 0.1128230410263 | myosin X |
| *Wfikkn1* | -1.278525284 | 0.1797058398484 | WAP, FS, Ig, KU, and NTR-containing protein 1 |
| *0610009O20Rik* | -1.279853569 | 0.0806264867343 | RIKEN cDNA 0610009O20 gene |
| *Gm11974* | -1.28300974 | 0.1161880322290 | . |
| *Arhgap39* | -1.283333496 | 0.1961992560025 | Rho GTPase activating protein 39 |
| *2510002D24Rik* | -1.287964108 | 0.1845818368233 | RIKEN cDNA 2510002D24 gene |
| *Akap14* | -1.288443376 | 0.1974493864460 | A kinase (PRKA) anchor protein 14 |
| *Pvr* | -1.289465829 | 0.1282143884334 | poliovirus receptor |
| *Slc2a4rg-ps* | -1.295594775 | 0.1989244707050 | Slc2a4 regulator, pseudogene |
| *Traf3ip1* | -1.300283189 | 0.0485199188238 | TRAF3 interacting protein 1 |
| *Stom* | -1.307121318 | 0.1165595281375 | stomatin |
| *Pccb* | -1.308645808 | 0.1385345626727 | propionyl Coenzyme A carboxylase, beta polypeptide |
| *Wdr91* | -1.308802672 | 0.0820626826584 | WD repeat domain 91 |
| *Zbtb12* | -1.309746513 | 0.1854299579279 | zinc finger and BTB domain containing 12 |
| *Adamts10* | -1.312089451 | 0.1156899499073 | a disintegrin-like and metallopeptidase (reprolysin type) with thrombospondin type 1 motif, 10 |
| *Cluap1* | -1.313657316 | 0.1149999366068 | clusterin associated protein 1 |
| *Zfp324* | -1.319261499 | 0.1287643612674 | zinc finger protein 324 |
| *Zfp260* | -1.31991526 | 0.0405248175510 | zinc finger protein 260 |
| *Eps8l1* | -1.323120627 | 0.0015187928981 | EPS8-like 1 |
| *Slc38a7* | -1.327688565 | 0.1653558668962 | solute carrier family 38, member 7 |
| *Ccdc94* | -1.337498818 | 0.0810178819854 | coiled-coil domain containing 94 |
| *Ncf2* | -1.337848748 | 0.1025360928066 | neutrophil cytosolic factor 2 |
| *Txndc16* | -1.33800209 | 0.0958744217604 | thioredoxin domain containing 16 |
| *1600014C10Rik* | -1.34121497 | 0.1264540272896 | RIKEN cDNA 1600014C10 gene |
| *6530402F18Rik* | -1.349542735 | 0.1324016801117 | RIKEN cDNA 6530402F18 gene |
| *Bcorl1* | -1.351800474 | 0.1020087962388 | BCL6 co-repressor-like 1 |
| *Tbc1d12* | -1.354947294 | 0.1475625825489 | TBC1D12: TBC1 domain family, member 12 |
| *5031434O11Rik* | -1.356808523 | 0.1521818791085 | RIKEN cDNA 5031434O11 gene |
| *Lace1* | -1.357819593 | 0.1723516762126 | lactation elevated 1 |
| *Olfr920* | -1.359152075 | 0.0935081835643 | olfactory receptor 920 |
| *Gstt2* | -1.361776761 | 0.0974285287811 | glutathione S-transferase, theta 2 |
| *Dst* | -1.361985665 | 0.1463143216715 | dystonin |
| *Fam171a2* | -1.362235981 | 0.0564801142883 | family with sequence similarity 171, member A2 |
| *Dtx3* | -1.363359295 | 0.0656271898014 | deltex 3, E3 ubiquitin ligase |
| *Mettl7a1* | -1.364655331 | 0.1276200674786 | methyltransferase like 7A1 |
| *Smpdl3b* | -1.369987654 | 0.1860714459613 | sphingomyelin phosphodiesterase, acid-like 3B |
| *Sult1a1* | -1.372022575 | 0.0630225673208 | sulfotransferase family 1A, phenol-preferring, member 1 |
| *Ubqln4* | -1.372629078 | 0.0698331201220 | ubiquilin 4 |
| *Bet1* | -1.374719438 | 0.1614287185264 | Bet1 golgi vesicular membrane trafficking protein |
| *Fbrsl1* | -1.375899676 | 0.0025478988938 | fibrosin-like 1 |
| *Jup* | -1.379695257 | 0.0789909304055 | junction plakoglobin |
| *Mlst8* | -1.380564486 | 0.1636180573357 | MTOR associated protein, LST8 homolog (S. cerevisiae) |
| *Maml1* | -1.381343934 | 0.1466646031797 | mastermind like 1 (Drosophila) |
| *Ankrd6* | -1.381371346 | 0.0263587872043 | ankyrin repeat domain 6 |
| *Svil* | -1.385536204 | 0.0614821045743 | supervillin |
| *Pwp2* | -1.385697296 | 0.1916848527696 | PWP2 periodic tryptophan protein homolog (yeast) |
| *Tmem230* | -1.387273919 | 0.1001839070301 | transmembrane protein 230 |
| *Tmem39b* | -1.39675541 | 0.0103268789033 | transmembrane protein 39b |
| *Pde1b* | -1.397045329 | 0.0007851866978 | phosphodiesterase 1B, Ca2+-calmodulin dependent |
| *Zfp764* | -1.399639016 | 0.0123393824707 | zinc finger protein 764 |
| *Zscan22* | -1.403937985 | 0.1522138289958 | zinc finger and SCAN domain containing 22 |
| *Csrnp1* | -1.409282027 | 0.1471269897138 | cysteine-serine-rich nuclear protein 1 |
| *C920021L13Rik* | -1.409749027 | 0.1047444246528 | RIKEN cDNA C920021L13 gene |
| *Syne3* | -1.41423075 | 0.0623165473666 | spectrin repeat containing, nuclear envelope family member 3 |
| *Zfp942* | -1.415377044 | 0.0470606592009 | zinc finger protein 942 |
| *Pde4a* | -1.415635885 | 0.0796225313994 | phosphodiesterase 4A, cAMP specific |
| *Gm15417* | -1.418255919 | 0.1281637870321 | predicted gene 15417 |
| *Mylk* | -1.422766865 | 0.0797202610005 | myosin, light polypeptide kinase |
| *N4bp2l1* | -1.425620199 | 0.0788976784883 | NEDD4 binding protein 2-like 1 |
| *Tmem64* | -1.426314991 | 0.0011792203332 | transmembrane protein 64 |
| *Chchd5* | -1.426582323 | 0.1686220554365 | coiled-coil-helix-coiled-coil-helix domain containing 5 |
| *Gm5512* | -1.431374327 | 0.0949584325433 | predicted gene 5512 |
| *BC027231* | -1.431580669 | 0.1447145277527 | . |
| *Fam83h* | -1.431748802 | 0.0052120710249 | family with sequence similarity 83, member H |
| *Mettl22* | -1.433501224 | 0.0836766455924 | methyltransferase like 22 |
| *9330020H09Rik* | -1.434767269 | 0.0786049820229 | RIKEN cDNA 9330020H09 gene |
| *Arhgef39* | -1.437158412 | 0.1458142368051 | Rho guanine nucleotide exchange factor (GEF) 39 |
| *Sec22c* | -1.437787593 | 0.0066009544696 | SEC22 homolog C, vesicle trafficking protein |
| *Ikbkg* | -1.443010767 | 0.1656372628665 | inhibitor of kappaB kinase gamma |
| *Mob1a* | -1.444253283 | 0.1449315986949 | MOB kinase activator 1A |
| *Trp53rka* | -1.445763239 | 0.0049057379329 | transformation related protein 53 regulating kinase A |
| *Ly96* | -1.447566313 | 0.1425463745815 | lymphocyte antigen 96 |
| *Serac1* | -1.45273802 | 0.1163021281803 | serine active site containing 1 |
| *Swsap1* | -1.457807092 | 0.0425173494251 | SWIM type zinc finger 7 associated protein 1 |
| *Chrm3* | -1.458618089 | 0.1568331582268 | cholinergic receptor, muscarinic 3, cardiac |
| *Sorl1* | -1.461756986 | 0.0872591852723 | sortilin-related receptor, LDLR class A repeats-containing |
| *Tnfrsf13c* | -1.462238025 | 0.0525226685249 | tumor necrosis factor receptor superfamily, member 13c |
| *Leng8* | -1.464717647 | 0.1695624008387 | leukocyte receptor cluster (LRC) member 8 |
| *Slfn3* | -1.466049409 | 0.0930982919786 | schlafen 3 |
| *Mrgbp* | -1.46641686 | 0.0010592976427 | MRG /MORF4L binding protein |
| *Asah2* | -1.468549265 | 0.1752898589281 | N-acylsphingosine amidohydrolase 2 |
| *Trim3* | -1.472023003 | 0.1647093059045 | tripartite motif-containing 3 |
| *Heg1* | -1.474750598 | 0.0691110585778 | heart development protein with EGF-like domains 1 |
| *Dnmt3a* | -1.484244596 | 0.0473639989454 | DNA methyltransferase 3A |
| *Mybpc3* | -1.488737007 | 0.1731839800581 | myosin binding protein C, cardiac |
| *Foxp1* | -1.489709701 | 0.1340933630803 | forkhead box P1 |
| *Ubqln2* | -1.493595382 | 0.0534897480762 | ubiquilin 2 |
| *Slc5a2* | -1.499973538 | 0.0044563885997 | solute carrier family 5 (sodium /glucose cotransporter), member 2 |
| *Mx1* | -1.500890889 | 0.0213457303660 | MX dynamin-like GTPase 1 |
| *Fbxo32* | -1.508404814 | 0.0130516282876 | F-box protein 32 |
| *4931406H21Rik* | -1.510963228 | 0.0592385255873 | RIKEN cDNA 4931406H21 gene |
| *Ankrd23* | -1.526333802 | 0.1112309957019 | ankyrin repeat domain 23 |
| *Cog7* | -1.526735064 | 0.1941422635902 | component of oligomeric golgi complex 7 |
| *Cd226* | -1.527560903 | 0.0584673521864 | CD226 antigen |
| *Zc3h12a* | -1.537914802 | 0.0739041824320 | zinc finger CCCH type containing 12A |
| *Prdm9* | -1.540357302 | 0.1845249225445 | PR domain containing 9 |
| *Pde4b* | -1.543992536 | 0.0131292780521 | phosphodiesterase 4B, cAMP specific |
| *Pigp* | -1.545066966 | 0.1024089738851 | phosphatidylinositol glycan anchor biosynthesis, class P |
| *Plekha1* | -1.550154511 | 0.0965243110084 | pleckstrin homology domain containing, family A (phosphoinositide binding specific) member 1 |
| *Trf* | -1.550360889 | 0.0682770008749 | transferrin |
| *Ppp1r13b* | -1.562406366 | 0.1817573593958 | protein phosphatase 1, regulatory (inhibitor) subunit 13B |
| *Cpeb2* | -1.563275463 | 0.1809923362545 | cytoplasmic polyadenylation element binding protein 2 |
| *Zfp534* | -1.565401769 | 0.0000199775510 | zinc finger protein 534 |
| *Rmnd1* | -1.566674662 | 0.1450983567239 | required for meiotic nuclear division 1 homolog |
| *Phf21a* | -1.572714212 | 0.1087695549849 | PHD finger protein 21A |
| *Gm29766* | -1.574528016 | 0.1163810278145 | . |
| *Snhg20* | -1.57655984 | 0.0052400367678 | small nucleolar RNA host gene 20 |
| *Ap5s1* | -1.579473639 | 0.0740753459689 | adaptor-related protein 5 complex, sigma 1 subunit |
| *Rnft1* | -1.588359185 | 0.0506557752000 | ring finger protein, transmembrane 1 |
| *Frat1* | -1.593823424 | 0.1116880343979 | frequently rearranged in advanced T cell lymphomas |
| *Bcl2l2* | -1.595044662 | 0.1749602786130 | BCL2-like 2 |
| *Ccrl2* | -1.595921493 | 0.0457467886069 | chemokine (C-C motif) receptor-like 2 |
| *Ube2e2* | -1.597592613 | 0.0263569131504 | ubiquitin-conjugating enzyme E2E 2 |
| *Sowahc* | -1.598941026 | 0.1827681784922 | sosondowah ankyrin repeat domain family member C |
| *Vamp4* | -1.599637355 | 0.0163473145545 | vesicle-associated membrane protein 4 |
| *Lamtor2* | -1.600672301 | 0.0128828455264 | late endosomal /lysosomal adaptor, MAPK and MTOR activator 2 |
| *Zfp932* | -1.606790387 | 0.1005050857552 | zinc finger protein 932 |
| *Pex6* | -1.607869128 | 0.0955605104866 | peroxisomal biogenesis factor 6 |
| *Ggact* | -1.61027067 | 0.0158568269163 | gamma-glutamylamine cyclotransferase |
| *Gm4432* | -1.614148294 | 0.0570578733213 | predicted gene 4432 |
| *Kctd14* | -1.615612693 | 0.0001154693322 | potassium channel tetramerisation domain containing 14 |
| *Haus2* | -1.618326161 | 0.0093787644555 | HAUS augmin-like complex, subunit 2 |
| *Slc25a14* | -1.624466467 | 0.0000806290567 | solute carrier family 25 (mitochondrial carrier, brain), member 14 |
| *Aars2* | -1.625660059 | 0.0887103643658 | alanyl-tRNA synthetase 2, mitochondrial |
| *5430416N02Rik* | -1.625759764 | 0.0924367738725 | RIKEN cDNA 5430416N02 gene |
| *Igsf9* | -1.627613242 | 0.0434045361023 | immunoglobulin superfamily, member 9 |
| *Dstyk* | -1.636272669 | 0.1272688072384 | dual serine /threonine and tyrosine protein kinase |
| *Tpk1* | -1.640625896 | 0.0672574565986 | thiamine pyrophosphokinase |
| *Prkab1* | -1.640825731 | 0.1460977082132 | protein kinase, AMP-activated, beta 1 non-catalytic subunit |
| *Zfp808* | -1.645944404 | 0.1577680107515 | zinc finger protein 80 |
| *Cldn1* | -1.645967158 | 0.0724844775968 | claudin 1 |
| *Slc44a1* | -1.649474542 | 0.1223617373770 | solute carrier family 44, member 1 |
| *Cry2* | -1.653894605 | 0.0341843312808 | cryptochrome 2 (photolyase-like) |
| *Satb1* | -1.654170028 | 0.1560507482125 | special AT-rich sequence binding protein 1 |
| *F630028O10Rik* | -1.656090435 | 0.1179574713866 | RIKEN cDNA F630028O10 gene |
| *Tmem150cos* | -1.65669377 | 0.0651656862313 | transmembrane protein 150C, opposite strand |
| *Cerkl* | -1.656960931 | 0.0405889676244 | ceramide kinase-like |
| *Rhbdd2* | -1.658295996 | 0.0089676373837 | rhomboid domain containing 2 |
| *Gm6377* | -1.658908002 | 0.1534463939497 | predicted gene 6377 |
| *Apc2* | -1.663007802 | 0.1379090698930 | adenomatosis polyposis coli 2 |
| *Zswim6* | -1.664916059 | 0.0094509731873 | zinc finger SWIM-type containing 6 |
| *Tspyl2* | -1.666059305 | 0.0006983604075 | TSPY-like 2 |
| *Ky* | -1.666514657 | 0.0486071885493 | kyphoscoliosis peptidase |
| *Bckdha* | -1.675094067 | 0.0550953339302 | branched chain ketoacid dehydrogenase E1, alpha polypeptide |
| *Cd101* | -1.67762406 | 0.1628890540680 | CD101 antigen |
| *Taf1a* | -1.686073761 | 0.1383983871809 | TATA-box binding protein associated factor, RNA polymerase I, A |
| *Mpst* | -1.690107341 | 0.0017475731280 | mercaptopyruvate sulfurtransferase |
| *Ankrd52* | -1.696623251 | 0.0620349386021 | ankyrin repeat domain 52 |
| *Cdc16* | -1.700568695 | 0.1524451490366 | CDC16 cell division cycle 16 |
| *Atxn1* | -1.703611345 | 0.1933071754156 | ataxin 1 |
| *Ninl* | -1.708951465 | 0.1986378819554 | ninein-like |
| *Rptor* | -1.71088528 | 0.1959318405650 | regulatory associated protein of MTOR, complex 1 |
| *Gla* | -1.71226233 | 0.1141203049419 | galactosidase, alpha |
| *Procr* | -1.713866019 | 0.0961396444477 | protein C receptor, endothelial |
| *Pyhin1* | -1.714230083 | 0.1078722129431 | pyrin and HIN domain family, member 1 |
| *D930048N14Rik* | -1.717402195 | 0.1106233300316 | RIKEN cDNA D930048N14 gene |
| *Gca* | -1.718085146 | 0.1166901489038 | grancalcin |
| *Stam* | -1.728120197 | 0.0508508202604 | signal transducing adaptor molecule (SH3 domain and ITAM motif) 1 |
| *Gadd45b* | -1.740535604 | 0.0936150526606 | growth arrest and DNA-damage-inducible 45 beta |
| *Zfp82* | -1.740962545 | 0.0236032340273 | zinc finger protein 82 |
| *Chchd10* | -1.748281992 | 0.1840072441632 | coiled-coil-helix-coiled-coil-helix domain containing 10 |
| *Grpel2* | -1.749791874 | 0.0632864341253 | GrpE-like 2, mitochondrial |
| *Klhl25* | -1.752855186 | 0.0558423804152 | kelch-like 25 |
| *Tnni2* | -1.753667671 | 0.1308042260983 | troponin I, skeletal, fast 2 |
| *Pdcd4* | -1.758923499 | 0.1863771039790 | programmed cell death 4 |
| *Mrpl50* | -1.763930748 | 0.0174462764884 | mitochondrial ribosomal protein L50 |
| *Sccpdh* | -1.769240772 | 0.0674225619310 | saccharopine dehydrogenase (putative) |
| *6330418K02Rik* | -1.769465448 | 0.1922905641018 | RIKEN cDNA 6330418K02 gene |
| *Rin1* | -1.772045723 | 0.0166679145097 | Ras and Rab interactor 1 |
| *Akr1b10* | -1.776671707 | 0.0017756046391 | aldo-keto reductase family 1, member B10 (aldose reductase) |
| *Rpap2* | -1.783695017 | 0.1130252797275 | RNA polymerase II associated protein 2 |
| *1700056E22Rik* | -1.784112795 | 0.1440703341524 | RIKEN cDNA 1700056E22 gene |
| *Insl6* | -1.786557539 | 0.0455833371027 | insulin-like 6 |
| *Mapk7* | -1.791234764 | 0.1661333445517 | mitogen-activated protein kinase 7 |
| *Cry1* | -1.79834291 | 0.0321379465574 | cryptochrome 1 (photolyase-like) |
| *Rangrf* | -1.799551145 | 0.1307103729815 | RAN guanine nucleotide release factor |
| *Ccdc125* | -1.800707616 | 0.0253291285206 | coiled-coil domain containing 125 |
| *Ttc28* | -1.802571929 | 0.0991116390483 | tetratricopeptide repeat domain 28 |
| *Cxcl9* | -1.803032697 | 0.0704440767985 | chemokine (C-X-C motif) ligand 9 |
| *Zbtb11os1* | -1.805562588 | 0.0137290243768 | zinc finger and BTB domain containing 11, opposite strand 1 |
| *Itgax* | -1.809332366 | 0.1395811280048 | integrin alpha X |
| *Klhl24* | -1.809338863 | 0.0040082460519 | kelch-like 24 |
| *Igsf6* | -1.812535648 | 0.0220736733645 | immunoglobulin superfamily, member 6 |
| *Tmem180* | -1.814165548 | 0.1571438512355 | transmembrane protein 180 |
| *Gm16523* | -1.815165054 | 0.0342463807122 | predicted gene, 16523 |
| *Prcc* | -1.821815508 | 0.1212570208544 | papillary renal cell carcinoma (translocation-associated) |
| *AW209491* | -1.827262369 | 0.0219947414347 | expressed sequence AW209491 |
| *Slc25a36* | -1.830316992 | 0.0861689247589 | solute carrier family 25, member 36 |
| *Fam195b* | -1.841961876 | 0.0432794423201 | family with sequence similarity 195, member B |
| *BC094916* | -1.852029411 | 0.1591084608972 | cDNA sequence BC094916 |
| *Clnk* | -1.852585563 | 0.1917639790632 | cytokine-dependent hematopoietic cell linker |
| *Ramp1* | -1.852912316 | 0.0621478061078 | receptor (calcitonin) activity modifying protein 1 |
| *Baiap2l1* | -1.852952193 | 0.1451785315925 | BAI1-associated protein 2-like 1 |
| *Ccdc163* | -1.855662055 | 0.0047098514906 | coiled-coil domain containing 163 |
| *C030034I22Rik* | -1.863536563 | 0.0871101702846 | RIKEN cDNA C030034I22 gene |
| *Lpar3* | -1.864723193 | 0.0714099189304 | lysophosphatidic acid receptor 3 |
| *Lyz1* | -1.865531744 | 0.1072663295074 | lysozyme 1 |
| *Man2b2* | -1.868252595 | 0.0600100675945 | mannosidase 2, alpha B2 |
| *Slc25a26* | -1.877937043 | 0.0433439417489 | solute carrier family 25 (mitochondrial carrier, phosphate carrier), member 26 |
| *Speg* | -1.879082584 | 0.0008316756720 | SPEG complex locus |
| *Gbp4* | -1.879998361 | 0.0465977210456 | guanylate binding protein 4 |
| *Trim68* | -1.883005448 | 0.1160137765301 | tripartite motif-containing 68 |
| *Kbtbd3* | -1.88837266 | 0.0444342499686 | kelch repeat and BTB (POZ) domain containing 3 |
| *Grap* | -1.888402498 | 0.0901749221259 | GRB2-related adaptor protein |
| *Specc1* | -1.893664669 | 0.0188117657422 | sperm antigen with calponin homology and coiled-coil domains 1 |
| *Klc2* | -1.893761767 | 0.1246755170900 | kinesin light chain 2 |
| *Nid2* | -1.893843408 | 0.0325740477609 | nidogen 2 |
| *Ppp1r3d* | -1.896210857 | 0.1988425267808 | protein phosphatase 1, regulatory subunit 3D |
| *Fam120c* | -1.906036342 | 0.0766984524788 | family with sequence similarity 120, member C |
| *Cyp4v3* | -1.907446646 | 0.0469932439686 | cytochrome P450, family 4, subfamily v, polypeptide 3 |
| *Cbr1* | -1.908294609 | 0.0672659700629 | carbonyl reductase 1 |
| *Ercc2* | -1.90878843 | 0.1827140279742 | excision repair cross-complementing rodent repair deficiency, complementation group 2 |
| *Pld2* | -1.920245766 | 0.0570405406955 | phospholipase D2 |
| *Lmntd2* | -1.924687622 | 0.1083005325044 | lamin tail domain containing 2 |
| *Fam53a* | -1.927282385 | 0.1137865398889 | family with sequence similarity 53, member A |
| *Carns1* | -1.928025266 | 0.0190091993287 | carnosine synthase 1 |
| *Clmp* | -1.928103898 | 0.0578762847521 | CXADR-like membrane protein |
| *Mx2* | -1.930666688 | 0.0651133050607 | MX dynamin-like GTPase 2 |
| *Zhx3* | -1.931199699 | 0.1291441003904 | zinc fingers and homeoboxes 3 |
| *Nup62-il4i1* | -1.933901268 | 0.0268343518377 | . |
| *Kifc3* | -1.938771882 | 0.0800911582839 | kinesin family member C3 |
| *Ccdc149* | -1.947187887 | 0.0734655279602 | coiled-coil domain containing 149 |
| *Asgr2* | -1.9549401 | 0.0496388257270 | asialoglycoprotein receptor 2 |
| *Spn* | -1.955039797 | 0.1644875327642 | sialophorin |
| *Cdan1* | -1.963328659 | 0.1321064592486 | congenital dyserythropoietic anemia, type I (human) |
| *Cd209a* | -1.96820985 | 0.1733047994150 | CD209a antigen |
| *Hist1h2be* | -1.974756722 | 0.0250450876034 | histone cluster 1, H2be |
| *Ctc1* | -1.975128046 | 0.1258724904728 | CTS telomere maintenance complex component 1 |
| *Sirt3* | -1.982719368 | 0.1774843756185 | sirtuin 3 |
| *Mmp19* | -1.983816943 | 0.1116559390682 | matrix metallopeptidase 19 |
| *Mmaa* | -1.990700133 | 0.0497404933875 | methylmalonic aciduria (cobalamin deficiency) type A |
| *Irak2* | -1.992229858 | 0.0836856920477 | interleukin-1 receptor-associated kinase 2 |
| *Grasp* | -1.995056747 | 0.0707651292334 | GRP1 (general receptor for phosphoinositides 1)-associated scaffold protein |
| *Man1c1* | -2.022795489 | 0.0191305072637 | mannosidase, alpha, class 1C, member 1 |
| *Pitpnm2* | -2.023903635 | 0.0489673404030 | phosphatidylinositol transfer protein, membrane-associated 2 |
| *Marcksl1* | -2.02521449 | 0.0047834693145 | MARCKS-like 1 |
| *Pigb* | -2.025533243 | 0.0781381511268 | phosphatidylinositol glycan anchor biosynthesis, class B |
| *Cd209e* | -2.025863882 | 0.0647565576528 | CD209e antigen |
| *Tnni3* | -2.027214138 | 0.1565556145575 | troponin I, cardiac 3 |
| *Nhsl2* | -2.027335943 | 0.1168385376428 | NHS-like 2 |
| *Ptpro* | -2.028503301 | 0.0184833872036 | protein tyrosine phosphatase, receptor type, O |
| *Nedd4* | -2.035127164 | 0.0983451573185 | neural precursor cell expressed, developmentally down-regulated 4 |
| *Miip* | -2.036452416 | 0.0135866485822 | migration and invasion inhibitory protein |
| *Notch4* | -2.037479115 | 0.0690435674990 | notch 4 |
| *Tcp11l2* | -2.043671813 | 0.0202195319413 | t-complex 11 (mouse) like 2 |
| *B130006D01Rik* | -2.043712312 | 0.0776626564850 | RIKEN cDNA B130006D01 gene |
| *Fam210a* | -2.046958137 | 0.0044712708100 | family with sequence similarity 210, member A |
| *Cd247* | -2.047774564 | 0.1014538726932 | CD247 antigen |
| *Lcor* | -2.048820625 | 0.0066123179574 | ligand dependent nuclear receptor corepressor |
| *Zfp874a* | -2.048827044 | 0.0280207283170 | zinc finger protein 874a |
| *Rhov* | -2.049641199 | 0.0974285287811 | ras homolog family member V |
| *1110032A03Rik* | -2.051225365 | 0.0161708624163 | RIKEN cDNA 1110032A03 gene |
| *Lmo1* | -2.052067349 | 0.0482268878314 | LIM domain only 1 |
| *Dnase1l2* | -2.052195113 | 0.0214715471027 | deoxyribonuclease 1-like 2 |
| *Marveld1* | -2.079944877 | 0.0141388216124 | MARVEL (membrane-associating) domain containing 1 |
| *Ift43* | -2.080572371 | 0.0402910840135 | intraflagellar transport 43 |
| *Gprc5a* | -2.088041505 | 0.0140159578227 | G protein-coupled receptor, family C, group 5, member A |
| *Snora33* | -2.09150049 | 0.0683918017697 | small nucleolar RNA, H /ACA box 33 |
| *Il12rb2* | -2.099812678 | 0.0520982697031 | interleukin 12 receptor, beta 2 |
| *Ankrd63* | -2.104814253 | 0.0180000368376 | ankyrin repeat domain 63 |
| *A330040F15Rik* | -2.11373614 | 0.0419087408528 | RIKEN cDNA A330040F15 gene |
| *Slc25a15* | -2.114500268 | 0.0111972482922 | solute carrier family 25 (mitochondrial carrier ornithine transporter), member 15 |
| *Prdm16* | -2.115875583 | 0.0451260468163 | PR domain containing 16 |
| *Cblb* | -2.119251246 | 0.1092462315755 | Casitas B-lineage lymphoma b |
| *Fam160a2* | -2.131313966 | 0.1206307096783 | family with sequence similarity 160, member A2 |
| *B4galt7* | -2.136209283 | 0.0011538428663 | xylosylprotein beta1,4-galactosyltransferase, polypeptide 7 (galactosyltransferase I) |
| *Ptgfrn* | -2.137932929 | 0.0849744209940 | prostaglandin F2 receptor negative regulator |
| *Arhgap42* | -2.141636309 | 0.0439378970348 | Rho GTPase activating protein 42 |
| *Homez* | -2.153609206 | 0.0922846904584 | homeodomain leucine zipper-encoding gene |
| *Gpm6b* | -2.155315135 | 0.1662182902016 | glycoprotein m6b |
| *Clstn1* | -2.157550413 | 0.1987338236764 | calsyntenin 1 |
| *Tmem27* | -2.16231554 | 0.0227219996151 | transmembrane protein 27 |
| *Arrdc2* | -2.166506174 | 0.1923308741030 | arrestin domain containing 2 |
| *Dyrk3* | -2.168449359 | 0.0635677544013 | dual-specificity tyrosine-(Y)-phosphorylation regulated kinase 3 |
| *Spsb1* | -2.171605494 | 0.1559014993715 | splA /ryanodine receptor domain and SOCS box containing 1 |
| *Gbp8* | -2.173145889 | 0.0895589302276 | guanylate-binding protein 8 |
| *Unc13a* | -2.178085687 | 0.0111929295958 | unc-13 homolog A (C. elegans) |
| *Tceanc* | -2.183091439 | 0.1924800569611 | transcription elongation factor A (SII) N-terminal and central domain containing |
| *Gcsh* | -2.192816474 | 0.0695481109595 | glycine cleavage system protein H (aminomethyl carrier) |
| *Brsk1* | -2.194848529 | 0.0515176652657 | BR serine /threonine kinase 1 |
| *Nicn1* | -2.198301405 | 0.0362318021076 | nicolin 1 |
| *Tmem86b* | -2.219866191 | 0.0016103385302 | transmembrane protein 86B |
| *Ift74* | -2.237194103 | 0.0320002490211 | intraflagellar transport 74 |
| *Blcap* | -2.238823103 | 0.0358959686133 | bladder cancer associated protein |
| *Polr3c* | -2.240756952 | 0.0367860151281 | polymerase (RNA) III (DNA directed) polypeptide C |
| *Nr4a3* | -2.250589702 | 0.0179166729943 | nuclear receptor subfamily 4, group A, member 3 |
| *Tmem198b* | -2.258201899 | 0.1005725947777 | transmembrane protein 198b |
| *D430042O09Rik* | -2.266571605 | 0.0296674769019 | RIKEN cDNA D430042O09 gene |
| *Slc15a2* | -2.280959107 | 0.1087593412595 | solute carrier family 15 (H+ /peptide transporter), member 2 |
| *Cyb5d2* | -2.288787804 | 0.0641185333037 | cytochrome b5 domain containing 2 |
| *Pex10* | -2.292535498 | 0.0230157631665 | peroxisomal biogenesis factor 10 |
| *Ptprv* | -2.293332884 | 0.0074274250164 | protein tyrosine phosphatase, receptor type, V |
| *Clec4a4* | -2.293587923 | 0.0520818154562 | C-type lectin domain family 4, member a4 |
| *3830403N18Rik* | -2.298080645 | 0.0038484398125 | RIKEN cDNA 3830403N18 gene |
| *Arl5c* | -2.303939845 | 0.1999381355723 | ADP-ribosylation factor-like 5C |
| *Sbk1* | -2.31017801 | 0.0161732515368 | SH3-binding kinase 1 |
| *Adgrl1* | -2.312720122 | 0.0004339060691 | adhesion G protein-coupled receptor L1 |
| *Lacc1* | -2.319036674 | 0.0710960028136 | laccase (multicopper oxidoreductase) domain containing 1 |
| *Sema4b* | -2.323051919 | 0.1839147150846 | sema domain, immunoglobulin domain (Ig), transmembrane domain (TM) and short cytoplasmic domain, (semaphorin) 4B |
| *Slc22a23* | -2.325665132 | 0.1357878351301 | solute carrier family 22, member 23 |
| *Sik2* | -2.334384571 | 0.0591231001214 | salt inducible kinase 2 |
| *Ilvbl* | -2.342739574 | 0.0881987343279 | ilvB (bacterial acetolactate synthase)-like |
| *Tmem159* | -2.35635352 | 0.0398169134784 | transmembrane protein 159 |
| *Morn1* | -2.356836005 | 0.1299201507829 | MORN repeat containing 1 |
| *Ntn3* | -2.357000452 | 0.1467677630927 | netrin 3 |
| *Kdm4c* | -2.361415155 | 0.1644793474204 | lysine (K)-specific demethylase 4C |
| *Ccr7* | -2.365278757 | 0.1166969833104 | chemokine (C-C motif) receptor 7 |
| *Arl11* | -2.367017353 | 0.0799557788058 | ADP-ribosylation factor-like 11 |
| *Tchh* | -2.374223098 | 0.0463030651147 | trichohyalin |
| *Evi5l* | -2.392045244 | 0.0288298076982 | ecotropic viral integration site 5 like |
| *H2-Q4* | -2.419510476 | 0.0566830636906 | histocompatibility 2, Q region locus 4 |
| *Gm11837* | -2.424376921 | 0.1451490134834 | predicted gene 11837 |
| *Necab1* | -2.424376921 | 0.1451490134834 | N-terminal EF-hand calcium binding protein 1 |
| *Acad9* | -2.424913596 | 0.1147475221094 | acyl-Coenzyme A dehydrogenase family, member 9 |
| *Fstl1* | -2.42563185 | 0.0341616488659 | follistatin-like 1 |
| *Leng9* | -2.428451454 | 0.1023802813785 | leukocyte receptor cluster (LRC) member 9 |
| *Katnb1* | -2.429210726 | 0.1062049395750 | katanin p80 (WD40-containing) subunit B 1 |
| *Fra10ac1* | -2.431842487 | 0.0230408945226 | FRA10AC1 homolog (human) |
| *Chpt1* | -2.440995196 | 0.1891719861482 | choline phosphotransferase 1 |
| *Ctsl* | -2.458245762 | 0.0208624112033 | cathepsin L |
| *Arl6ip5* | -2.4587934 | 0.0084732198678 | ADP-ribosylation factor-like 6 interacting protein 5 |
| *Cahm* | -2.459679946 | 0.0795595832586 | colon adenocarcinoma hypermethylated RNA |
| *Ell3* | -2.493391869 | 0.0889189702267 | elongation factor RNA polymerase II-like 3 |
| *Il13ra1* | -2.499494541 | 0.0205673869981 | interleukin 13 receptor, alpha 1 |
| *H2-Eb2* | -2.500257413 | 0.1823185354204 | histocompatibility 2, class II antigen E beta2 |
| *Vamp1* | -2.504411458 | 0.0937922317339 | vesicle-associated membrane protein 1 |
| *Myof* | -2.505815945 | 0.0980228149226 | myoferlin |
| *Cmc4* | -2.510078438 | 0.0054288283104 | C-x(9)-C motif containing 4 |
| *BC051226* | -2.512898338 | 0.0167339907089 | cDNA sequence BC051226 |
| *Inpp5a* | -2.519019203 | 0.0332084697439 | inositol polyphosphate-5-phosphatase A |
| *Ift81* | -2.533151042 | 0.0065255647481 | intraflagellar transport 81 |
| *Mbd5* | -2.559462262 | 0.0308840209724 | methyl-CpG binding domain protein 5 |
| *Celsr1* | -2.567913918 | 0.1327863144427 | cadherin, EGF LAG seven-pass G-type receptor 1 |
| *Mfge8* | -2.570012569 | 0.1405285727103 | milk fat globule-EGF factor 8 protein |
| *Grhpr* | -2.573989354 | 0.0360880218504 | glyoxylate reductase /hydroxypyruvate reductase |
| *Cyp4f13* | -2.577614298 | 0.0161580812316 | cytochrome P450, family 4, subfamily f, polypeptide 13 |
| *Pop7* | -2.578785449 | 0.0292485493212 | processing of precursor 7, ribonuclease P family, (S. cerevisiae) |
| *Lrrc28* | -2.580215374 | 0.0216236513093 | leucine rich repeat containing 28 |
| *Mitf* | -2.594095738 | 0.1193744496734 | microphthalmia-associated transcription factor |
| *Hpgd* | -2.612009514 | 0.1229769863473 | hydroxyprostaglandin dehydrogenase 15 (NAD) |
| *Ints12* | -2.618427565 | 0.0213168420067 | integrator complex subunit 12 |
| *Galc* | -2.624567146 | 0.0771998893472 | galactosylceramidase |
| *Dyrk2* | -2.636587635 | 0.0775259910669 | dual-specificity tyrosine-(Y)-phosphorylation regulated kinase 2 |
| *Zfp945* | -2.639252541 | 0.1454274551629 | zinc finger protein 945 |
| *Pcnx* | -2.655368929 | 0.0013521184500 | pecanex homolog (Drosophila) |
| *2310034G01Rik* | -2.680757066 | 0.1263590365969 | RIKEN cDNA 2310034G01 gene |
| *Gm13807* | -2.681304469 | 0.1133771208472 | predicted gene 13807 |
| *Tbc1d8b* | -2.6820703 | 0.0224078293975 | TBC1 domain family, member 8B |
| *2610035D17Rik* | -2.684693187 | 0.0934776471294 | RIKEN cDNA 2610035D17 gene |
| *St6galnac6* | -2.71282845 | 0.0275677566716 | ST6 (alpha-N-acetyl-neuraminyl-2,3-beta-galactosyl-1, 3)-N-acetylgalactosaminide alpha-2,6-sialyltransferase 6 |
| *4932438H23Rik* | -2.719245158 | 0.0142302345349 | RIKEN cDNA 4932438H23 gene |
| *Fam132b* | -2.727562544 | 0.0517518656831 | family with sequence similarity 132, member B |
| *Flrt3* | -2.728325477 | 0.1170328078792 | fibronectin leucine rich transmembrane protein 3 |
| *Iqsec2* | -2.745962408 | 0.0422198205288 | IQ motif and Sec7 domain 2 |
| *Zfp467* | -2.748313584 | 0.0044239993621 | zinc finger protein 467 |
| *Fscn1* | -2.780781322 | 0.0115664815351 | fascin actin-bundling protein 1 |
| *Fktn* | -2.789697403 | 0.1049095308607 | fukutin |
| *Gm9776* | -2.806206365 | 0.1756908537863 | predicted gene 9776 |
| *Il15ra* | -2.806763761 | 0.0065570928207 | interleukin 15 receptor, alpha chain |
| *Kbtbd6* | -2.837335715 | 0.0323526333642 | kelch repeat and BTB (POZ) domain containing 6 |
| *Zfp963* | -2.850091264 | 0.0795363506382 | zinc finger protein 963 |
| *Maml2* | -2.876550351 | 0.0713540638632 | mastermind like 2 (Drosophila) |
| *Hist2h2be* | -2.886765082 | 0.0306935140468 | histone cluster 2, H2be |
| *Clec4a2* | -2.889835436 | 0.0082778775531 | C-type lectin domain family 4, member a2 |
| *Abtb1* | -2.936252069 | 0.1894431868330 | ankyrin repeat and BTB (POZ) domain containing 1 |
| *Akap2* | -2.957839013 | 0.1579611976102 | A kinase (PRKA) anchor protein 2 |
| *Myb* | -2.975289761 | 0.0326537978505 | myeloblastosis oncogene |
| *Nkx6-2* | -2.978091201 | 0.0281592550248 | NK6 homeobox 2 |
| *Dnal1* | -2.998359684 | 0.0236386964106 | dynein, axonemal, light chain 1 |
| *Frat2* | -2.999434866 | 0.1747565705248 | frequently rearranged in advanced T cell lymphomas 2 |
| *Gpr162* | -3.012417566 | 0.1652695435685 | G protein-coupled receptor 162 |
| *Cntnap1* | -3.02047189 | 0.0276967106934 | contactin associated protein-like 1 |
| *Pcgf3* | -3.021738298 | 0.0904694238333 | polycomb group ring finger 3 |
| *Tmcc3* | -3.044331307 | 0.0005085680815 | transmembrane and coiled coil domains 3 |
| *Hnmt* | -3.053631034 | 0.1238688162641 | histamine N-methyltransferase |
| *Slc27a3* | -3.141113391 | 0.1634771670029 | solute carrier family 27 (fatty acid transporter), member 3 |
| *Siglech* | -3.233238676 | 0.1543530279677 | sialic acid binding Ig-like lectin H |
| *Omg* | -3.276350132 | 0.0017640795085 | oligodendrocyte myelin glycoprotein |
| *Gm10677* | -3.286086822 | 0.0024154000438 | predicted gene 10677 |
| *Egfr* | -3.295071805 | 0.0038484398125 | epidermal growth factor receptor |
| *Spata13* | -3.313392437 | 0.0241649789396 | spermatogenesis associated 13 |
| *Ccl5* | -3.352489392 | 0.0047993603966 | chemokine (C-C motif) ligand 5 |
| *H2-M2* | -3.527281077 | 0.1493707529745 | histocompatibility 2, M region locus 2 |
| *Itga4* | -3.55761245 | 0.0348920363905 | integrin alpha 4 |
| *Tmem55a* | -3.584487856 | 0.0010017776699 | transmembrane protein 55A |
| *Ccl22* | -3.584534529 | 0.0110549266195 | chemokine (C-C motif) ligand 22 |
| *F11r* | -3.665525156 | 0.1080059561142 | F11 receptor |
| *Gpc2* | -3.739382929 | 0.0340841924497 | glypican 2 (cerebroglycan) |
| *Sfxn5* | -3.764250424 | 0.1383817868875 | sideroflexin 5 |
| *Il12b* | -3.934794361 | 0.1395999300457 | interleukin 12b |
| *Zfp608* | -3.95848503 | 0.0297144005907 | zinc finger protein 608 |
| *Cd207* | -4.255954723 | 0.0021338527180 | CD207 antigen |
| *Cd209c* | -4.343500431 | 0.1909000152683 | CD209c antigen |
